# Proceedings of the 8th Annual Conference on the Science of Dissemination and Implementation: Washington, DC, USA. 14-15 December 2015

**DOI:** 10.1186/s13012-016-0452-0

**Published:** 2016-08-01

**Authors:** David Chambers, Lisa Simpson, Felicia Hill-Briggs, Gila Neta, Cynthia Vinson, David Chambers, Rinad Beidas, Steven Marcus, Gregory Aarons, Kimberly Hoagwood, Sonja Schoenwald, Arthur Evans, Matthew Hurford, Ronnie Rubin, Trevor Hadley, Frances Barg, Lucia Walsh, Danielle Adams, David Mandell, Lindsey Martin, Joseph Mignogna, Juliette Mott, Natalie Hundt, Michael Kauth, Mark Kunik, Aanand Naik, Jeffrey Cully, Alan McGuire, Dominique White, Tom Bartholomew, John McGrew, Lauren Luther, Angie Rollins, Michelle Salyers, Brittany Cooper, Angie Funaiole, Julie Richards, Amy Lee, Gwen Lapham, Ryan Caldeiro, Paula Lozano, Tory Gildred, Carol Achtmeyer, Evette Ludman, Megan Addis, Larry Marx, Katharine Bradley, Tonya VanDeinse, Amy Blank Wilson, Burgin Stacey, Byron Powell, Alicia Bunger, Gary Cuddeback, Miya Barnett, Nicole Stadnick, Lauren Brookman-Frazee, Anna Lau, Shannon Dorsey, Michael Pullmann, Shannon Mitchell, Robert Schwartz, Arethusa Kirk, Kristi Dusek, Marla Oros, Colleen Hosler, Jan Gryczynski, Carolina Barbosa, Laura Dunlap, David Lounsbury, Kevin O’Grady, Barry Brown, Laura Damschroder, Thomas Waltz, Byron Powell, Mona Ritchie, Thomas Waltz, David Atkins, Zac E. Imel, Bo Xiao, Doğan Can, Panayiotis Georgiou, Shrikanth Narayanan, Cady Berkel, Carlos Gallo, Irwin Sandler, C. Hendricks Brown, Sharlene Wolchik, Anne Marie Mauricio, Carlos Gallo, C. Hendricks Brown, Sanjay Mehrotra, Dharmendra Chandurkar, Siddhartha Bora, Arup Das, Anand Tripathi, Niranjan Saggurti, Anita Raj, Eric Hughes, Brian Jacobs, Eric Kirkendall, Danielle Loeb, Katy Trinkley, Michael Yang, Andrew Sprowell, Donald Nease, Aaron Lyon, Cara Lewis, Meredith Boyd, Abigail Melvin, Semret Nicodimos, Freda Liu, Nathanial Jungbluth, Aaron Lyon, Cara Lewis, Meredith Boyd, Abigail Melvin, Semret Nicodimos, Freda Liu, Nathanial Jungbluth, Allen Flynn, Zach Landis-Lewis, Anne Sales, Jure Baloh, Marcia Ward, Xi Zhu, Ian Bennett, Jurgen Unutzer, Johnny Mao, Enola Proctor, Mindy Vredevoogd, Ya-Fen Chan, Nathaniel Williams, Phillip Green, Steven Bernstein, June-Marie Rosner, Michelle DeWitt, Jeanette Tetrault, James Dziura, Allen Hsiao, Scott Sussman, Patrick O’Connor, Benjamin Toll, Michael Jones, Julie Gassaway, Jonathan Tobin, Douglas Zatzick, Angela R. Bradbury, Linda Patrick-Miller, Brian Egleston, Olufunmilayo I. Olopade, Michael J. Hall, Mary B. Daly, Linda Fleisher, Generosa Grana, Pamela Ganschow, Dominique Fetzer, Amanda Brandt, Dana Farengo-Clark, Andrea Forman, Rikki S. Gaber, Cassandra Gulden, Janice Horte, Jessica Long, Rachelle Lorenz Chambers, Terra Lucas, Shreshtha Madaan, Kristin Mattie, Danielle McKenna, Susan Montgomery, Sarah Nielsen, Jacquelyn Powers, Kim Rainey, Christina Rybak, Michelle Savage, Christina Seelaus, Jessica Stoll, Jill Stopfer, Shirley Yao, Susan Domchek, Erin Hahn, Corrine Munoz-Plaza, Jianjin Wang, Jazmine Garcia Delgadillo, Brian Mittman, Michael Gould, Shuting (Lily) Liang, Michelle C. Kegler, Megan Cotter, Emily Phillips, April Hermstad, Rentonia Morton, Derrick Beasley, Jeremy Martinez, Kara Riehman, David Gustafson, Lisa Marsch, Louise Mares, Andrew Quanbeck, Fiona McTavish, Helene McDowell, Randall Brown, Chantelle Thomas, Joseph Glass, Joseph Isham, Dhavan Shah, Jane Liebschutz, Karen Lasser, Katherine Watkins, Allison Ober, Sarah Hunter, Karen Lamp, Brett Ewing, Juliet Iwelunmor, Joyce Gyamfi, Sarah Blackstone, Nana Kofi Quakyi, Jacob Plange-Rhule, Gbenga Ogedegbe, Pritika Kumar, Nancy Van Devanter, Nam Nguyen, Linh Nguyen, Trang Nguyen, Nguyet Phuong, Donna Shelley, Sian Rudge, Etienne Langlois, Andrea Tricco, Sherry Ball, Anne Lambert-Kerzner, Christine Sulc, Carol Simmons, Jeneen Shell-Boyd, Taryn Oestreich, Ashley O’Connor, Emily Neely, Marina McCreight, Amy Labebue, Doreen DiFiore, Diana Brostow, P. Michael Ho, David Aron, Jillian Harvey, Megan McHugh, Dennis Scanlon, Rebecca Lee, Erica Soltero, Nathan Parker, Lorna McNeill, Tracey Ledoux, Jessie-Lee McIsaac, Kate MacLeod, Nicole Ata, Sherry Jarvis, Sara Kirk, Jonathan Purtle, Elizabeth Dodson, Ross Brownson, Brian Mittman, Geoffrey Curran, Geoffrey Curran, Jeffrey Pyne, Gregory Aarons, Mark Ehrhart, Elisa Torres, Edward Miech, Edward Miech, Kathleen Stevens, I.S.R.N. Steering Council, Alison Hamilton, Deborah Cohen, Deborah Padgett, Alexandra Morshed, Rupa Patel, Beth Prusaczyk, David C. Aron, Divya Gupta, Sherry Ball, Rosa Hand, Jenica Abram, Taylor Wolfram, Molly Hastings, Sarah Moreland-Russell, Rachel Tabak, Alex Ramsey, Ana Baumann, Emily Kryzer, Katherine Montgomery, Ericka Lewis, Margaret Padek, Byron Powell, Ross Brownson, Cezar Brian Mamaril, Glen Mays, Keith Branham, Lava Timsina, Glen Mays, Rachel Hogg, Abigail Fagan, Valerie Shapiro, Eric Brown, Kevin Haggerty, David Hawkins, Sabrina Oesterle, David Hawkins, Richard Catalano, Virginia McKay, M. Margaret Dolcini, Lee Hoffer, Tannaz Moin, Jinnan Li, O. Kenrik Duru, Susan Ettner, Norman Turk, Charles Chan, Abigail Keckhafer, Robert Luchs, Sam Ho, Carol Mangione, Peter Selby, Laurie Zawertailo, Nadia Minian, Dolly Balliunas, Rosa Dragonetti, Sarwar Hussain, Julia Lecce, Matthew Chinman, Joie Acosta, Patricia Ebener, Patrick S. Malone, Mary Slaughter, Darcy Freedman, Susan Flocke, Eunlye Lee, Kristen Matlack, Erika Trapl, Punam Ohri-Vachaspati, Morgan Taggart, Elaine Borawski, Amanda Parrish, Jeffrey Harris, Marlana Kohn, Kristen Hammerback, Becca McMillan, Peggy Hannon, Taren Swindle, Geoffrey Curran, Leanne Whiteside-Mansell, Wendy Ward, Cheryl Holt, Sheri Lou Santos, Erin Tagai, Mary Ann Scheirer, Roxanne Carter, Janice Bowie, Muhiuddin Haider, Jimmie Slade, Min Qi Wang, Andrew Masica, Gerald Ogola, Candice Berryman, Kathleen Richter, Rachel Shelton, Lina Jandorf, Deborah Erwin, Khoa Truong, Joyce R. Javier, Dean Coffey, Sheree M. Schrager, Lawrence Palinkas, Jeanne Miranda, Veda Johnson, Valerie Hutcherson, Ruth Ellis, Anna Kharmats, Sandra Marshall-King, Monica LaPradd, Fannie Fonseca-Becker, Deanna Kepka, Julia Bodson, Echo Warner, Brynn Fowler, Elizabeth Shenkman, William Hogan, Folakami Odedina, Jessica De Leon, Monica Hooper, Olveen Carrasquillo, Renee Reams, Myra Hurt, Steven Smith, Jose Szapocznik, David Nelson, Prabir Mandal, James Teufel

**Affiliations:** 1 Division of Cancer Control and Population Sciences, National Cancer Institute, National Institutes of Health, Rockville, MD 20850 USA; 2 AcademyHealth, Washington, DC 20036 USA; 3 Department of Medicine and Welch Center for Prevention, Epidemiology and Clinical Research, Johns Hopkins Medical Institutions, Baltimore, MD 21287 USA; 4 Division of Cancer Control and Population Sciences, National Cancer Institute, National Institutes of Health, Rockville, MD 20850 USA; 5 Division of Cancer Control and Population Sciences, National Cancer Institute, National Institutes of Health, Rockville, MD 20850 USA; 6 Psychiatry, University of Pennsylvania, Philadelphia, PA 19104 USA; 7 School of Social Policy and Practice, University of Pennsylvania, Philadelphia, PA 19103 USA; 8 Psychiatry, UC San Diego, La Jolla, CA 92083-0812 USA; 9 Child & Adolescent Psychiatry, The Child Study Center at NYU Langone Medical Center, New York, NY, New York, NY 10016 USA; 10 Psychiatry and Behavioral Sciences, MUSC, Charleston, SC 29425 USA; 11 DBHIDS, City of Philadelphia, Philadelphia, PA 19104 USA; 12 CBH, City of Philadelphia, Philadelphia, PA 19104 USA; 13 Family Medicine and Community Health, University of Pennsylvania, Philadelphia, PA 19104 USA; 14 Psychiatry, University of Pennsylvania Perelman School of Medicine, Philadelphia, PA 19107 USA; 15 Health Services Research & Development, Department of Veterans Affairs & Baylor College of Medicine, Houston, TX 77030 USA; 16 Treatment Core, Department of Veterans Affairs, Waco, TX 76711 USA; 17 National Center for PTSD, Executive Division, Department of Veterans Affairs, White River Junction, VT 05009 USA; 18 HSR&D, HSR&D Center for Health Information and Communication, Roudebush VA Medical Center, Indianapolis, IN 46202 USA; 19 Psychology, Indiana University Purdue University Indianapolis, Indianapolis, IN 46202 USA; 20 Department of Psychiatric Rehabilitation and Counseling, Rutgers University, Newark, NJ 07107 USA; 21 Health Services Research & Development, Richard L. Roudebush VAMC, Indianapolis, IN 46202 USA; 22 IUPUI Department of Psychology, ACT Center of Indiana, Indianapolis, IN 46202 USA; 23 Human Development, Washington State University, Pullman, WA 99164 USA; 24 Prevention Science, Washington State University, Pullman, WA 99164 USA; 25 Group Health Research Institute, Group Health, Seattle, WA 98101 USA; 26 Behavioral Health Services, Group Health, Seattle, WA 98101 USA; 27 Preventive Care, Group Health, Seattle, WA 98101 USA; 28 Health Services Research & Development, Primary and Specialty Medical Care Service, VA Puget Sound, Seattle, WA 98108 USA; 29 School of Social Work, University of North Carolina at Chapel Hill, Chapel Hill, NC 27599 USA; 30 Health Policy & Management, The University of North Carolina at Chapel Hill, Chapel Hill, NC 27566-7411 USA; 31 College of Social Work, Ohio State University, Columbus, OH 43210 USA; 32 Psychology, University of California, Los Angeles, Los Angeles, CA 90095 USA; 33 Psychiatry, University of California, San Diego, La Jolla, CA 92093 USA; 34 Psychology, University of Washington, Seattle, WA 98195 USA; 35 Department of Psychiatry and Behavioral Sciences, Division of Public Behavioral Health, University of Washington, Seattle, WA 98102 USA; 36 Social Research Center, Friends Research Institute, Baltimore, MD 21201 USA; 37 Pediatrics, Total Health Care, Baltimore, MD 21217 USA; 38 The Mosaic Group, The Mosaic Group, Baltimore, MD 21210 USA; 39 Behavioral Health Economics Program, RTI International, Chicago, IL 60606-4901 USA; 40 Center for Interdisciplinary Substance Abuse Research, Research Triangle Institute, Rockville, MD 20852 USA; 41 Epidemiology and Population Health, Albert Einstein College of Medicine, Bronx, NY 10467 USA; 42 Division of Community Collaboration and Implementation Science, Albert Einstein College of Medicine of Yeshiva University, Bronx, NY 10467 USA; 43 Department of Psychology, University of Maryland, College Park, College Park, MD 20742 USA; 44 Psychology, University of North Carolina at Wilmington, Wilmington, NC 28403 USA; 45 Department of Veterans Affairs, VA Center for Clinical Management Research, Ann Arbor, MI 48105 USA; 46 Psychology, Eastern Michigan University, Ypsilanti, MI 48197 USA; 47 Health Policy & Management, The University of North Carolina at Chapel Hill, Chapel Hill, NC 27566 USA; 48 VA QUERI Program for Team-Based Behavioral Health, Department of Veterans Affairs, North Little Rock, AR 72114 USA; 49 Psychology, Eastern Michigan University, Ypsilanti, MI 48197 USA; 50 Health Services Research & Development, VA Center for Clinical Management Research, Ann Arbor, MI 48105 USA; 51 Department of Psychiatry and Behavioral Sciences, University of Washington, Seattle, WA 98105 USA; 52 Educational Psychology, University of Utah, Salt Lake City, UT 84112 USA; 53 Electrical Engineering, University of Southern California, Los Angeles, CA 90089 USA; 54 Computer Science, University of Southern California, Los Angeles, CA 90089 USA; 55 Electrical Engineering & Computer Science, University of Southern California, Los Angeles, CA 90089 USA; 56 REACH Institute, Arizona State University, Tempe, AZ 85284 USA; 57 Center for Prevention Implementation Methodology, Northwestern University, Chicago, IL 60611 USA; 58 Psychology, Arizona State University, Tempe, AZ 85287 USA; 59 Center for Prevention Implementation Methodology, Northwestern University, Chicago, IL 60611 USA; 60 Industrial Engineering, Northwestern University, Chicago, IL 60611 USA; 61 Research, Sambodhi Research and Communications Private Limited, Noida, 201301 India; 62 Monitoring and Evaluation, India Health Action Trust, Lucknow, 226001 India; 63 Monitoring, Learning and Evaluation, Bill and Melinda Gates Foundation, New Delhi, 110057 India; 64 Division of Global Public Health, Department of Medicine, University of California, San Diego, San Diego, CA 92093 USA; 65 Information Technology, MITRE, Bedford, MA 01730 USA; 66 VP, CMIO, CIO, Children’s National Health System, Washington, DC, 20010 USA; 67 Association CMIO, Cincinnati Children’s Hospital and Medical Center, Cincinnati, OH 45229 USA; 68 Department of Medicine/Division of General Internal Medicine, University of Colorado School of Medicine, Aurora, CO 80045 USA; 69 Department of Clinical Pharmacy, University of Colorado School of Pharmacy, Aurora, CO 80045 USA; 70 University of Colorado School of Medicine, Aurora, CO 80045 USA; 71 Department of Family Medicine, University of Colorado School of Medicine, Aurora, CO 80238 USA; 72 Psychiatry and Behavioral Sciences, University of Washington, Seattle, WA 98115 USA; 73 Psychological and Brain Sciences, Indiana University, Bloomington, IN 47405 USA; 74 Psychology, University of Washington, Seattle, WA 98115 USA; 75 Psychiatry and Behavioral Sciences, University of Washington, Seattle, WA 98115 USA; 76 Psychological and Brain Sciences, Indiana University, Bloomington, IN 47405 USA; 77 Psychology, University of Washington, Seattle, WA 98115 USA; 78 Learning Health Sciences, Medical School, University of Michigan, Ann Arbor, MI 48109 USA; 79 Learning Health Sciences, University of Michigan Medical School, Ann Arbor, MI 48109 USA; 80 Learning Health Sciences, University of Michigan, Ann Arbor, MI 48109 USA; 81 Center for Clinical Management Research, VA Ann Arbor Health Care System, Ann Arbor, MI 48109 USA; 82 Department of Health Management and Policy, University of Iowa, Iowa City, IA 52242 USA; 83 Family Medicine, University of Washington, Seattle, WA 98105-6099 USA; 84 Psychiatry & Behavioral Sciences, University of Washington, Seattle, WA 98195 USA; 85 Center for Clinical and Epidemiological Research, University of Washington, Seattle, WA 98101 USA; 86 George Warren Brown School of Social Work, Washington University in St. Louis, Saint Louis, MO 63130 USA; 87 Social Work, Boise State University, Boise, ID 83725 USA; 88 School of Social Work, University of Tennessee, Knoxville, TN 37996 USA; 89 Emergency Medicine, Yale University School of Medicine, New Haven, CT 06519 USA; 90 Cancer Prevention and Control, Yale Cancer Center, New Haven, CT 06519 USA; 91 Information Technology Services, Yale-New Haven Hospital, New Haven, CT 06510 USA; 92 Medicine, Yale School of Medicine, New Haven, CT 06519 USA; 93 Pediatrics and Emergency Medicine, Yale-New Haven Hospital, New Haven, CT 06510 USA; 94 Medicine, Yale-New Haven Hospital, New Haven, CT 06510 USA; 95 Internal Medicine, Yale University School of Medicine, New Haven, CT 06520 USA; 96 Public Health Sciences, Medical University of South Carolina, Charleston, SC 29425 USA; 97 Research, Shepherd Center, Atlanta, GA 30312 USA; 98 Virginia C. Crawford Research Institute, Atlanta, GA 30309 USA; 99 Community Engaged Research, Clinical Directors Network, Inc. & The Rockefeller University, New York, NY 10018 USA; 100 Department of Psychiatry and Behavioral Sciences, University of Washington, Seattle, WA 98104 USA; 101 Division of Hematology-Oncology, Department of Medicine, The University of Pennsylvania, Philadelphia, PA 19104 USA; 102 Department of Medical Ethics and Health Policy, The University of Pennsylvania, Philadelphia, PA 19104 USA; 103 Center for Clinical Cancer Genetics Department of Medicine, Hematology/Oncology, The University of Chicago, Chicago, IL 60637 USA; 104 Biostatistics and Bioinformatics Facility, The Fox Chase Cancer Center, Philadelphia, PA 19111 USA; 105 Center for Clinical Cancer Genetics, The University of Chicago, Chicago, IL 60637 USA; 106 Clinical Genetics/Gastrointestinal Risk Assessment, The Fox Chase Cancer Center, Philadelphia, PA 19111 USA; 107 Department of Clinical Genetics, The Fox Chase Cancer Center, Philadelphia, PA 19111 USA; 108 Center for Injury Research and Prevention, The Children’s Hospital of Philadelphia, Philadelphia, PA 19104 USA; 109 Hematology and Medical Oncology, MD Anderson Cancer Center at Cooper, Camden, NJ 08043 USA; 110 Internal Medicine, John H. Stroger, Jr. Hospital, Chicago, IL 60612 USA; 111 Mariann and Robert MacDonald Women’s Cancer Risk Evaluation Program, The University of Pennsylvania, Philadelphia, PA 19104 USA; 112 Cancer Genetics Program, MD Anderson Cancer Center at Cooper, Camden, NJ 08043 USA; 113 Breast and Cervical Cancer Screening Program, John H. Stroger, Jr. Hospital, Chicago, IL 60612 USA; 114 Cancer Screening Program, John H. Stroger, Jr. Hospital, Chicago, IL 60612 USA; 115 Comprehensive Cancer Risk and Prevention Clinic, The University of Chicago, Chicago, IL 60637 USA; 116 Research and Evaluation, Kaiser Permanente Southern California, Pasadena, CA 91101 USA; 117 Emory Prevention Research Center, Emory University Rollins School of Public Health, Atlanta, GA 30322 USA; 118 Research Institute, Palo Alto Medical Foundation, Palo Alto, CA 94301 USA; 119 Department of Behavioral Sciences and Health Education, Emory University Rollins School of Public Health, Atlanta, GA 30322 USA; 120 Behavioral Sciences and Health Education, Rollins School of Public Health, Emory Prevention Research Center, Atlanta, GA 30322 USA; 121 Statistics and Evaluation Center, American Cancer Society, Atlanta, GA 30303 USA; 122 Center for Health Enhancement Systems Studies, University of Wisconsin – Madison, Madison, WI 53706 USA; 123 Center for Technology and Behavioral Health, Dartmouth College, Dartmouth, NH 03755 USA; 124 Communication Arts, University of Wisconsin – Madison, Madison, WI 53706 USA; 125 Industrial and Systems Engineering, University of Wisconsin – Madison, Madison, WI 53706 USA; 126 Family Medicine, Population Health Sciences, University of Wisconsin School of Medicine & Public Health, Madison, WI 53715 USA; 127 Access Community Health Center, Access Community Health Center, Madison, WI 53706 USA; 128 School of Social Work, University of Wisconsin – Madison, Madison, WI 54706 USA; 129 Communication Sciences, University of Wisconsin – Madison, Madison, WI 53706 USA; 130 Clinical Addiction Research and Education (CARE) Unit, Boston Medical Center/Boston University School of Medicine, Boston, MA 02118 USA; 131 Boston University School of Public Health, Boston, MA 02118 USA; 132 RAND Corporation, RAND Corporation, Santa Monica, CA 90407 USA; 133 Venice Family Clinic, Venice Family Clinic, Venice, CA 90291 USA; 134 Kinesiology and Community Health, University of Illinois at Urbana Champaign, Champaign, IL 61822 USA; 135 Center for Healthful Behavior Change, New York University, New York, NY 10016 USA; 136 Global Institute of Public Health, New York University, New York, NY 10003 USA; 137 Physiology, Kwame Nkrumah University of Science and Technology, Kumasi, 00000 Ghana; 138 Population Health and Medicine, New York University, New York, NY 10016 USA; 139 Department of Population Health, New York University School of Medicine, New York, NY 10016 USA; 140 College of Nursing, New York University, New York, NY 10075 USA; 141 Institute of Social and Medical Sciences, Institute of Social and Medical Sciences, Hanoi, Vietnam; 142 Knowledge Exchange Division, Sax Institute, Ultimo, NSW 2007 Australia; 143 Alliance for Health Policy and Systems Research, World Health Organization, Geneva, 1211 Switzerland; 144 Dalla Lana School of Public Health/St Michael’s Hospital, University of Toronto, Toronto, ON M5B 1W8 Canada; 145 Research, Department of Veterans Affairs, Cleveland, OH 44106 USA; 146 Research, Eastern Colorado Health Care System, Department of Veterans Affairs, Denver, CO 80220 USA; 147 University of Colorado, Denver, CO 80204 USA; 148 Center of Innovation for Veteran-Centered and Value-Driven Care, VA Puget Sound Health Care System, Seattle, WA 98108 USA; 149 Research Service, Cleveland VA Medical Center, Cleveland, OH 44106 USA; 150 Denver-Seattle Center of Innovation, Eastern Colorado Health Care System, Aurora, CO 80045 USA; 151 Research, Cleveland VA Medical Center, Cleveland, OH 44106 USA; 152 Denver-Seattle Center of Innovation, VA Eastern Colorado Health Care System, Denver, CO 80220 USA; 153 Education Department, Louis Stokes Cleveland VA Medical Center, Cleveland, OH 44106 USA; 154 Medicine, Case Western Reserve University School of Medicine, Cleveland, OH USA; 155 Health Professions, Medical University of South Carolina, Charleston, SC 29425 USA; 156 Healthcare Leadership and Management, Medical University of South Carolina, Charleston, SC 29425 USA; 157 Center for Healthcare Studies, Northwestern University, Chicago, IL 60611 USA; 158 Department of Health Policy and Administration, Pennsylvania State University, University Park, PA 16802 USA; 159 College of Nursing and Health Innovation, Arizona State University, Phoenix, AZ 85004 USA; 160 Health & Human Performance, University of Houston, Houston, TX 77204 USA; 161 Health Disparities Research, The University of Texas MD Anderson Cancer Center, Houston, TX 77030 USA; 162 School of Health and Human Performance, Dalhousie University, Halifax, NS B3H4R2 Canada; 163 Health Management &Policy, Drexel University School of Public Health, Philadelphia, PA 19130 USA; 164 Institute for Public Health, Washington University, St. Louis, MO 63112 USA; 165 Brown School and Prevention Research Center in St. Louis, Washington University in St. Louis, Saint Louis, MO 63130 USA; 166 Research and Evaluation, Kaiser Permanente Southern Calif/US Dept of Veterans Affairs, Pasadena, CA 91101 USA; 167 Department of Pharmacy Practice, University of Arkansas for Medical Sciences, Little Rock, AR 72205 USA; 168 Department of Pharmacy Practice, University of Arkansas for Medical Sciences, Little Rock, AR 72205 USA; 169 Psychiatry and Behavioral Sciences, University of Arkansas for Medical Sciences, Little Rock, AR 72205 USA; 170 Psychiatry, UC San Diego, La Jolla, CA 92083-0812 USA; 171 Department of Psychology, San Diego State University, San Diego, CA 92182-4611 USA; 172 HSR&D, Richard L. Roudebush VA Medical Center, Indianapolis, IN 46202 USA; 173 HSR&D, Richard L. Roudebush VA Medical Center, Indianapolis, IN 46202 USA; 174 Improvement Science Research Network, University of Texas Health Science Center San Antonio, San Antonio, TX 78229 USA; 175 HSR&D Center for the Study of Healthcare Innovation, Implementation and Policy, Department of Veterans Affairs, Los Angeles, CA 90073 USA; 176 UCLA Department of Psychiatry and Biobehavioral Sciences, UCLA, Los Angeles, CA 90024 USA; 177 Family Medicine, Oregon Health & Science University, Portland, OR 97239 USA; 178 Silver School of Social Work, New York University, New York, NY 10003 USA; 179 Brown School, Washington University in St. Louis, Saint Louis, MO 63130 USA; 180 Division of Infectious Diseases, Washington University School of Medicine, St. Louis, MO 63110 USA; 181 Brown School, Washington University in St. Louis, Saint Louis, MO 63110 USA; 182 Case Western Reserve University, Cleveland, OH USA; 183 Department of Veterans Affairs, Medicine, Louis Stokes Cleveland Medical Center, Cleveland, OH 44106 USA; 184 Research, Cleveland VA Medical Center, Cleveland, OH 44106 USA; 185 Research, Department of Veterans Affairs, Cleveland, OH 44106 USA; 186 Research, International, and Scientific Affairs, Academy of Nutrition and Dietetics, Chicago, IL 60606 USA; 187 Web Strategy, Academy of Nutrition and Dietetics, Chicago, IL 60606 USA; 188 Center for Public Health Systems Science, Washington University in St. Louis, St. Louis, MO 63112 USA; 189 Brown School and Prevention Research Center in St. Louis, Washington University in St. Louis, Saint Louis, MO 63130 USA; 190 School of Medicine, Washington University in St. Louis, St. Louis, MO 63110 USA; 191 Brown School, Washington University in St. Louis, St. Louis, MO 63110 USA; 192 Health Policy & Management, The University of North Carolina at Chapel Hill, Chapel Hill, NC 27566-7411 USA; 193 Health Management and Policy, University of Kentucky, College of Public Health, Lexington, KY 40536 USA; 194 Department of Health Services Management, University of Kentucky, College of Public Health, Lexington, KY 40536 USA; 195 Systems for Action National Program Office, University of Kentucky, College of Public Health, Lexington, KY 40536 USA; 196 Department of Health Services Management, University of Kentucky, College of Public Health, Lexington, KY 40536 USA; 197 College of Health Sciences, University of Kentucky, Lexington, KY 40536 USA; 198 Department of Sociology and Criminology & Law, University of Florida, Gainesville, FL 32611 USA; 199 School of Social Welfare, UC Berkeley, Berkeley, CA 94720 USA; 200 Department of Public Health Sciences, University of Miami, Miami, FL 33136 USA; 201 School of Social Work, University of Washington, Seattle, WA 98115 USA; 202 School of Social Work, University of Washington, Seattle, WA 98115 USA; 203 College of Public Health and Human Sciences, Oregon State University, Corvallis, OR 97331 USA; 204 School of Social and Behavioral Health Sciences, Oregon State University, Corvallis, OR 97331 USA; 205 Department of Anthropology, Case Western Reserve University, Cleveland, OH 44106 USA; 206 Division of General Internal Medicine and Health Services Research, David Geffen School of Medicine at University of California, Los Angeles, CA 90024 USA; 207 HSR&D Center for the Study of Healthcare Innovation, Implementation, and Policy, VA Greater Los Angeles Healthcare System, Los Angeles, CA 90073 USA; 208 Department of Health Policy and Management, Fielding School of Public Health, University of California, Los Angeles, CA 90095 USA; 209 Actuarial Pricing, UnitedHealthcare, Minneapolis, MN 55343 USA; 210 Actuarial Pricing, UnitedHealthcare, Minneapolis, MN 55369 USA; 211 Innovations, UnitedHealthcare, Minneapolis, MN 55343 USA; 212 Addictions Program, Centre for Addiction and Mental Health, Toronto, ON M5T1P7 Canada; 213 Family and Community Medicine, University of Toronto, Toronto, ON M5T1P7 Canada; 214 Psychiatry, Dalla Lana School of Public Health, University of Toronto, Toronto, ON M5T1P7 Canada; 215 Pharmacology and Toxicology, University of Toronto, Toronto, ON M5T1P7 Canada; 216 Addictions Medicine, Centre for Addiction and Mental Health, Toronto, ON M5S 3E3 Canada; 217 Nicotine Dependence Service, Centre for Addiction and Mental Health, Toronto, ON M5T 1P7 Canada; 218 Health, RAND Corporation, Pittsburgh, PA 15213 USA; 219 VISN 4 MIRECC, VA Pittsburgh Healthcare Center, Pittsburgh, PA 15206 USA; 220 Health, RAND Corporation, Arlington, VA 22202-5050 USA; 221 Health, RAND Corporation, Santa Monica, CA 90407 USA; 222 Epidemiology and Biostatistics, Case Western Reserve University, Cleveland, OH 44106 USA; 223 Prevention Research Center for Healthy Neighborhoods, Case Western Reserve University, Cleveland, OH 44106 USA; 224 Family Medicine, Case Western Reserve University, Cleveland, OH 44106 USA; 225 Mandel School of Applied Social Sciences, Case Western Reserve University, Cleveland, OH 44106 USA; 226 School of Nutrition and Health Promotion, Arizona State University, Phoenix, AZ 85004 USA; 227 Agreculture, St. Clair Superior Development Corporation, Cleveland, OH 44103 USA; 228 Department of Health Services, University of Washington, Health Promotion Research Center, Seattle, WA 98105 USA; 229 Health Promotion Research Center, University of Washington, Seattle, WA 98105 USA; 230 Partner Relationships, American Cancer Society, Seattle, WA 98109 USA; 231 Health Services, University of Washington, Seattle, WA 98105 USA; 232 Family and Preventive Medicine, University of Arkansas for Medical Sciences, Little Rock, AR 72205 USA; 233 Department of Pharmacy Practice, University of Arkansas for Medical Sciences, Little Rock, AR 72205 USA; 234 Pediatrics, University of Arkansas for Medical Sciences, Little Rock, AR 72205 USA; 235 Behavioral and Community Health, University of Maryland, College Park, MD 20742 USA; 236 Program Evaluation, Scheirer Consulting, Princeton, NJ 8540 USA; 237 Community Ministry of Prince George’s County, Upper Marlboro, MD 20772 USA; 238 Health Behavior and Society, Johns Hopkins University, Baltimore, MD 21205 USA; 239 Maryland Institute for Applied Environmental Health, University of Maryland, College Park, MD 20742 USA; 240 Center for Clinical Effectiveness, Baylor Scott & White Health, Dallas, TX 75206 USA; 241 Department of Sociomedical Sciences, Columbia University Mailman School of Public Health, New York, NY 10032 USA; 242 Oncological Sciences, Mount Sinai School of Medicine, New York, NY 10029 USA; 243 Division of Cancer Prevention and Population Sciences, Roswell Park Cancer Institute, Buffalo, NY 14263 USA; 244 Public Health Sciences, Clemson University, Clemson, SC 29634 USA; 245 Department of Pediatrics, Division of General Pediatrics, Chidren’s Hospital Los Angeles/USC Keck School of Medicine, Los Angeles, CA 90027 USA; 246 Division of Hospital Medicine, Chidren’s Hospital Los Angeles, Los Angeles, CA 90027 USA; 247 Social Work, University of Southern California, Los Angeles, CA 90089-0411 USA; 248 Semel Institute for Neuroscience and Human Behavior, UCLA Center for Health Services and Society, Los Angeles, CA 90024 USA; 249 Pediatrics, Emory University, Atlanta, GA 30303 USA; 250 Pediatrics, PARTNERS for Equity in Child and Adolescent Health, Atlanta, GA 30303 USA; 251 Johns Creek, GA 30022 USA; 252 Pediatrics, Emory University, Atlanta, GA 30303 USA; 253 International Health, Johns Hopkins Bloomberg School of Public Health, Baltimore, MD 21205 USA; 254 International Health, Global Obesity Prevention Center, Baltimore, MD 21205 USA; 255 Shawnee Christian Health Care Center, Louisville, KY 40212 USA; 256 Health, Behavior, & Society, Johns Hopkins Bloomberg School of Public Health, Baltimore, MD 21202 USA; 257 Huntsman Cancer Institute, University of Utah, College of Nursing and Huntsman Cancer Institute, Salt Lake City, UT 84112 USA; 258 Cancer Control and Population Sciences, Huntsman Cancer Institute & University of Utah, Salt Lake City, UT 84112 USA; 259 Department of Health Outcomes and Policy, University of Florida College of Medicine, Gainesville, FL 32606 USA; 260 Department of Pharmaceutical Outcomes and Policy, University of Florida, Gainesville, FL 32606 USA; 261 Clinical and Translational Science Institute, Florida State University College of Medicine, Tallahassee, FL 32308 USA; 262 Psychology, University of Miami, Miami, FL 33136 USA; 263 Department of Medicine, University of Miami, Miami, FL 33136 USA; 264 Department of Pharmaceutical Sciences, Florida A & M University, Tallahassee, FL 32308 USA; 265 College of Medicine, Florida State University, Tallahassee, FL 32308 USA; 266 Translational Research Institute, Florida Hospital, Orlando, FL 32804 USA; 267 Clinical and Translational Science Institute, University of Miami, Miami, FL 33136 USA; 268 Clinical and Translational Science Institute, University of Florida, Gainesville, FL 32606 USA; 269 Biology, Edward Waters College, Jacksonville, FL 32309 USA; 270 Public Health, Mercyhurst University, Erie, PA 16504 USA

## Abstract

A1 Introduction to the 8^th^ Annual Conference on the Science of Dissemination and Implementation: Optimizing Personal and Population Health

David Chambers, Lisa Simpson

D1 Discussion forum: Population health D&I research

Felicia Hill-Briggs

D2 Discussion forum: Global health D&I research

Gila Neta, Cynthia Vinson

D3 Discussion forum: Precision medicine and D&I research

David Chambers

S1 Predictors of community therapists’ use of therapy techniques in a large public mental health system

Rinad Beidas, Steven Marcus, Gregory Aarons, Kimberly Hoagwood, Sonja Schoenwald, Arthur Evans, Matthew Hurford, Ronnie Rubin, Trevor Hadley, Frances Barg, Lucia Walsh, Danielle Adams, David Mandell

S2 Implementing brief cognitive behavioral therapy (CBT) in primary care: Clinicians' experiences from the field

Lindsey Martin, Joseph Mignogna, Juliette Mott, Natalie Hundt, Michael Kauth, Mark Kunik, Aanand Naik, Jeffrey Cully

S3 Clinician competence: Natural variation, factors affecting, and effect on patient outcomes

Alan McGuire, Dominique White, Tom Bartholomew, John McGrew, Lauren Luther, Angie Rollins, Michelle Salyers

S4 Exploring the multifaceted nature of sustainability in community-based prevention: A mixed-method approach

Brittany Cooper, Angie Funaiole

S5 Theory informed behavioral health integration in primary care: Mixed methods evaluation of the implementation of routine depression and alcohol screening and assessment

Julie Richards, Amy Lee, Gwen Lapham, Ryan Caldeiro, Paula Lozano, Tory Gildred, Carol Achtmeyer, Evette Ludman, Megan Addis, Larry Marx, Katharine Bradley

S6 Enhancing the evidence for specialty mental health probation through a hybrid efficacy and implementation study

Tonya VanDeinse, Amy Blank Wilson, Burgin Stacey, Byron Powell, Alicia Bunger, Gary Cuddeback

S7 Personalizing evidence-based child mental health care within a fiscally mandated policy reform

Miya Barnett, Nicole Stadnick, Lauren Brookman-Frazee, Anna Lau

S8 Leveraging an existing resource for technical assistance: Community-based supervisors in public mental health

Shannon Dorsey, Michael Pullmann

S9 SBIRT implementation for adolescents in urban federally qualified health centers: Implementation outcomes

Shannon Mitchell, Robert Schwartz, Arethusa Kirk, Kristi Dusek, Marla Oros, Colleen Hosler, Jan Gryczynski, Carolina Barbosa, Laura Dunlap, David Lounsbury, Kevin O'Grady, Barry Brown

S10 PANEL: Tailoring Implementation Strategies to Context - Expert recommendations for tailoring strategies to context

Laura Damschroder, Thomas Waltz, Byron Powell

S11 PANEL: Tailoring Implementation Strategies to Context - Extreme facilitation: Helping challenged healthcare settings implement complex programs

Mona Ritchie

S12 PANEL: Tailoring Implementation Strategies to Context - Using menu-based choice tasks to obtain expert recommendations for implementing three high-priority practices in the VA

Thomas Waltz

S13 PANEL: The Use of Technology to Improve Efficient Monitoring of Implementation of Evidence-based Programs - Siri, rate my therapist: Using technology to automate fidelity ratings of motivational interviewing

David Atkins, Zac E. Imel, Bo Xiao, Doğan Can, Panayiotis Georgiou, Shrikanth Narayanan

S14 PANEL: The Use of Technology to Improve Efficient Monitoring of Implementation of Evidence-based Programs - Identifying indicators of implementation quality for computer-based ratings

Cady Berkel, Carlos Gallo, Irwin Sandler, C. Hendricks Brown, Sharlene Wolchik, Anne Marie Mauricio

S15 PANEL: The Use of Technology to Improve Efficient Monitoring of Implementation of Evidence-based Programs - Improving implementation of behavioral interventions by monitoring emotion in spoken speech

Carlos Gallo, C. Hendricks Brown, Sanjay Mehrotra

S16 Scorecards and dashboards to assure data quality of health management information system (HMIS) using R

Dharmendra Chandurkar, Siddhartha Bora, Arup Das, Anand Tripathi, Niranjan Saggurti, Anita Raj

S17 A big data approach for discovering and implementing patient safety insights

Eric Hughes, Brian Jacobs, Eric Kirkendall

S18 Improving the efficacy of a depression registry for use in a collaborative care model

Danielle Loeb, Katy Trinkley, Michael Yang, Andrew Sprowell, Donald Nease

S19 Measurement feedback systems as a strategy to support implementation of measurement-based care in behavioral health

Aaron Lyon, Cara Lewis, Meredith Boyd, Abigail Melvin, Semret Nicodimos, Freda Liu, Nathanial Jungbluth

S20 PANEL: Implementation Science and Learning Health Systems: Intersections and Commonalities - Common loop assay: Methods of supporting learning collaboratives

Allen Flynn

S21 PANEL: Implementation Science and Learning Health Systems: Intersections and Commonalities - Innovating audit and feedback using message tailoring models for learning health systems

Zach Landis-Lewis

S22 PANEL: Implementation Science and Learning Health Systems: Intersections and Commonalities - Implementation science and learning health systems: Connecting the dots

Anne Sales

S23 Facilitation activities of Critical Access Hospitals during TeamSTEPPS implementation

Jure Baloh, Marcia Ward, Xi Zhu

S24 Organizational and social context of federally qualified health centers and variation in maternal depression outcomes

Ian Bennett, Jurgen Unutzer, Johnny Mao, Enola Proctor, Mindy Vredevoogd, Ya-Fen Chan, Nathaniel Williams, Phillip Green

S25 Decision support to enhance treatment of hospitalized smokers: A randomized trial

Steven Bernstein, June-Marie Rosner, Michelle DeWitt, Jeanette Tetrault, James Dziura, Allen Hsiao, Scott Sussman, Patrick O’Connor, Benjamin Toll

S26 PANEL: Developing Sustainable Strategies for the Implementation of Patient-Centered Care across Diverse US Healthcare Systems - A patient-centered approach to successful community transition after catastrophic injury

Michael Jones, Julie Gassaway

S27 PANEL: Developing Sustainable Strategies for the Implementation of Patient-Centered Care across Diverse US Healthcare Systems - Conducting PCOR to integrate mental health and cancer screening services in primary care

Jonathan Tobin

S28 PANEL: Developing Sustainable Strategies for the Implementation of Patient-Centered Care across Diverse US Healthcare Systems - A comparative effectiveness trial of optimal patient-centered care for US trauma care systems

Douglas Zatzick

S29 Preferences for in-person communication among patients in a multi-center randomized study of in-person versus telephone communication of genetic test results for cancer susceptibility

Angela R Bradbury, Linda Patrick-Miller, Brian Egleston, Olufunmilayo I Olopade, Michael J Hall, Mary B Daly, Linda Fleisher, Generosa Grana, Pamela Ganschow, Dominique Fetzer, Amanda Brandt, Dana Farengo-Clark, Andrea Forman, Rikki S Gaber, Cassandra Gulden, Janice Horte, Jessica Long, Rachelle Lorenz Chambers, Terra Lucas, Shreshtha Madaan, Kristin Mattie, Danielle McKenna, Susan Montgomery, Sarah Nielsen, Jacquelyn Powers, Kim Rainey, Christina Rybak, Michelle Savage, Christina Seelaus, Jessica Stoll, Jill Stopfer, Shirley Yao and Susan Domchek

S30 Working towards de-implementation: A mixed methods study in breast cancer surveillance care

Erin Hahn, Corrine Munoz-Plaza, Jianjin Wang, Jazmine Garcia Delgadillo, Brian Mittman Michael Gould

S31Integrating evidence-based practices for increasing cancer screenings in safety-net primary care systems: A multiple case study using the consolidated framework for implementation research

Shuting (Lily) Liang, Michelle C. Kegler, Megan Cotter, Emily Phillips, April Hermstad, Rentonia Morton, Derrick Beasley, Jeremy Martinez, Kara Riehman

S32 Observations from implementing an mHealth intervention in an FQHC

David Gustafson, Lisa Marsch, Louise Mares, Andrew Quanbeck, Fiona McTavish, Helene McDowell, Randall Brown, Chantelle Thomas, Joseph Glass, Joseph Isham, Dhavan Shah

S33 A multicomponent intervention to improve primary care provider adherence to chronic opioid therapy guidelines and reduce opioid misuse: A cluster randomized controlled trial protocol

Jane Liebschutz, Karen Lasser

S34 Implementing collaborative care for substance use disorders in primary care: Preliminary findings from the summit study

Katherine Watkins, Allison Ober, Sarah Hunter, Karen Lamp, Brett Ewing

S35 Sustaining a task-shifting strategy for blood pressure control in Ghana: A stakeholder analysis

Juliet Iwelunmor, Joyce Gyamfi, Sarah Blackstone, Nana Kofi Quakyi, Jacob Plange-Rhule, Gbenga Ogedegbe

S36 Contextual adaptation of the consolidated framework for implementation research (CFIR) in a tobacco cessation study in Vietnam

Pritika Kumar, Nancy Van Devanter, Nam Nguyen, Linh Nguyen, Trang Nguyen, Nguyet Phuong, Donna Shelley

S37 Evidence check: A knowledge brokering approach to systematic reviews for policy

Sian Rudge

S38 Using Evidence Synthesis to Strengthen Complex Health Systems in Low- and Middle-Income Countries

Etienne Langlois

S39 Does it matter: timeliness or accuracy of results? The choice of rapid reviews or systematic reviews to inform decision-making

Andrea Tricco

S40 Evaluation of the veterans choice program using lean six sigma at a VA medical center to identify benefits and overcome obstacles

Sherry Ball, Anne Lambert-Kerzner, Christine Sulc, Carol Simmons, Jeneen Shell-Boyd, Taryn Oestreich, Ashley O'Connor, Emily Neely, Marina McCreight, Amy Labebue, Doreen DiFiore, Diana Brostow, P. Michael Ho, David Aron

S41 The influence of local context on multi-stakeholder alliance quality improvement activities: A multiple case study

Jillian Harvey, Megan McHugh, Dennis Scanlon

S42 Increasing physical activity in early care and education: Sustainability via active garden education (SAGE)

Rebecca Lee, Erica Soltero, Nathan Parker, Lorna McNeill, Tracey Ledoux

S43 Marking a decade of policy implementation: The successes and continuing challenges of a provincial school food and nutrition policy in Canada

Jessie-Lee McIsaac, Kate MacLeod, Nicole Ata, Sherry Jarvis, Sara Kirk

S44 Use of research evidence among state legislators who prioritize mental health and substance abuse issues

Jonathan Purtle, Elizabeth Dodson, Ross Brownson

S45 PANEL: Effectiveness-Implementation Hybrid Designs: Clarifications, Refinements, and Additional Guidance Based on a Systematic Review and Reports from the Field - Hybrid type 1 designs

Brian Mittman, Geoffrey Curran

S46 PANEL: Effectiveness-Implementation Hybrid Designs: Clarifications, Refinements, and Additional Guidance Based on a Systematic Review and Reports from the Field - Hybrid type 2 designs

Geoffrey Curran

S47 PANEL: Effectiveness-Implementation Hybrid Designs: Clarifications, Refinements, and Additional Guidance Based on a Systematic Review and Reports from the Field - Hybrid type 3 designs

Jeffrey Pyne

S48 Linking team level implementation leadership and implementation climate to individual level attitudes, behaviors, and implementation outcomes

Gregory Aarons, Mark Ehrhart, Elisa Torres

S49 Pinpointing the specific elements of local context that matter most to implementation outcomes: Findings from qualitative comparative analysis in the RE-inspire study of VA acute stroke care

Edward Miech

S50 The GO score: A new context-sensitive instrument to measure group organization level for providing and improving care

Edward Miech

S51 A research network approach for boosting implementation and improvement

Kathleen Stevens, I.S.R.N. Steering Council

S52 PANEL: Qualitative methods in D&I Research: Value, rigor and challenge - The value of qualitative methods in implementation research

Alison Hamilton

S53 PANEL: Qualitative methods in D&I Research: Value, rigor and challenge - Learning evaluation: The role of qualitative methods in dissemination and implementation research

Deborah Cohen

S54 PANEL: Qualitative methods in D&I Research: Value, rigor and challenge - Qualitative methods in D&I research

Deborah Padgett

S55 PANEL: Maps & models: The promise of network science for clinical D&I - Hospital network of sharing patients with acute and chronic diseases in California

Alexandra Morshed

S56 PANEL: Maps & models: The promise of network science for clinical D&I - The use of social network analysis to identify dissemination targets and enhance D&I research study recruitment for pre-exposure prophylaxis for HIV (PrEP) among men who have sex with men

Rupa Patel

S57 PANEL: Maps & models: The promise of network science for clinical D&I - Network and organizational factors related to the adoption of patient navigation services among rural breast cancer care providers

Beth Prusaczyk

S58 A theory of de-implementation based on the theory of healthcare professionals’ behavior and intention (THPBI) and the becker model of unlearning

David C. Aron, Divya Gupta, Sherry Ball

S59 Observation of registered dietitian nutritionist-patient encounters by dietetic interns highlights low awareness and implementation of evidence-based nutrition practice guidelines

Rosa Hand, Jenica Abram, Taylor Wolfram

S60 Program sustainability action planning: Building capacity for program sustainability using the program sustainability assessment tool

Molly Hastings, Sarah Moreland-Russell

S61 A review of D&I study designs in published study protocols

Rachel Tabak, Alex Ramsey, Ana Baumann, Emily Kryzer, Katherine Montgomery, Ericka Lewis, Margaret Padek, Byron Powell, Ross Brownson

S62 PANEL: Geographic variation in the implementation of public health services: Economic, organizational, and network determinants - Model simulation techniques to estimate the cost of implementing foundational public health services

Cezar Brian Mamaril, Glen Mays, Keith Branham, Lava Timsina

S63 PANEL: Geographic variation in the implementation of public health services: Economic, organizational, and network determinants - Inter-organizational network effects on the implementation of public health services

Glen Mays, Rachel Hogg

S64 PANEL: Building capacity for implementation and dissemination of the communities that care prevention system at scale to promote evidence-based practices in behavioral health - Implementation fidelity, coalition functioning, and community prevention system transformation using communities that care

Abigail Fagan, Valerie Shapiro, Eric Brown

S65 PANEL: Building capacity for implementation and dissemination of the communities that care prevention system at scale to promote evidence-based practices in behavioral health - Expanding capacity for implementation of communities that care at scale using a web-based, video-assisted training system

Kevin Haggerty, David Hawkins

S66 PANEL: Building capacity for implementation and dissemination of the communities that care prevention system at scale to promote evidence-based practices in behavioral health - Effects of communities that care on reducing youth behavioral health problems

Sabrina Oesterle, David Hawkins, Richard Catalano

S68 When interventions end: the dynamics of intervention de-adoption and replacement

Virginia McKay, M. Margaret Dolcini, Lee Hoffer

S69 Results from next-d: can a disease specific health plan reduce incident diabetes development among a national sample of working-age adults with pre-diabetes?

Tannaz Moin, Jinnan Li, O. Kenrik Duru, Susan Ettner, Norman Turk, Charles Chan, Abigail Keckhafer, Robert Luchs, Sam Ho, Carol Mangione

S70 Implementing smoking cessation interventions in primary care settings (STOP): using the interactive systems framework

Peter Selby, Laurie Zawertailo, Nadia Minian, Dolly Balliunas, Rosa Dragonetti, Sarwar Hussain, Julia Lecce

S71 Testing the Getting To Outcomes implementation support intervention in prevention-oriented, community-based settings

Matthew Chinman, Joie Acosta, Patricia Ebener, Patrick S Malone, Mary Slaughter

S72 Examining the reach of a multi-component farmers’ market implementation approach among low-income consumers in an urban context

Darcy Freedman, Susan Flocke, Eunlye Lee, Kristen Matlack, Erika Trapl, Punam Ohri-Vachaspati, Morgan Taggart, Elaine Borawski

S73 Increasing implementation of evidence-based health promotion practices at large workplaces: The CEOs Challenge

Amanda Parrish, Jeffrey Harris, Marlana Kohn, Kristen Hammerback, Becca McMillan, Peggy Hannon

S74 A qualitative assessment of barriers to nutrition promotion and obesity prevention in childcare

Taren Swindle, Geoffrey Curran, Leanne Whiteside-Mansell, Wendy Ward

S75 Documenting institutionalization of a health communication intervention in African American churches

Cheryl Holt, Sheri Lou Santos, Erin Tagai, Mary Ann Scheirer, Roxanne Carter, Janice Bowie, Muhiuddin Haider, Jimmie Slade, Min Qi Wang

S76 Reduction in hospital utilization by underserved patients through use of a community-medical home

Andrew Masica, Gerald Ogola, Candice Berryman, Kathleen Richter

S77 Sustainability of evidence-based lay health advisor programs in African American communities: A mixed methods investigation of the National Witness Project

Rachel Shelton, Lina Jandorf, Deborah Erwin

S78 Predicting the long-term uninsured population and analyzing their gaps in physical access to healthcare in South Carolina

Khoa Truong

S79 Using an evidence-based parenting intervention in churches to prevent behavioral problems among Filipino youth: A randomized pilot study

Joyce R. Javier, Dean Coffey, Sheree M. Schrager, Lawrence Palinkas, Jeanne Miranda

S80 Sustainability of elementary school-based health centers in three health-disparate southern communities

Veda Johnson, Valerie Hutcherson, Ruth Ellis

S81 Childhood obesity prevention partnership in Louisville: creative opportunities to engage families in a multifaceted approach to obesity prevention

Anna Kharmats, Sandra Marshall-King, Monica LaPradd, Fannie Fonseca-Becker

S82 Improvements in cervical cancer prevention found after implementation of evidence-based Latina prevention care management program

Deanna Kepka, Julia Bodson, Echo Warner, Brynn Fowler

S83 The OneFlorida data trust: Achieving health equity through research & training capacity building

Elizabeth Shenkman, William Hogan, Folakami Odedina, Jessica De Leon, Monica Hooper, Olveen Carrasquillo, Renee Reams, Myra Hurt, Steven Smith, Jose Szapocznik, David Nelson, Prabir Mandal

S84 Disseminating and sustaining medical-legal partnerships: Shared value and social return on investment

James Teufel

## Introduction

### A1 Introduction to the 8^th^ Annual Conference on the Science of Dissemination and Implementation: Optimizing Personal and Population Health

#### David Chambers^1^, Lisa Simpson^2^

##### ^1^Division of Cancer Control and Population Sciences, National Cancer Institute, National Institutes of Health, Rockville, MD, 20850, USA; ^2^AcademyHealth, Washington, DC, 20036, USA

For the second year in a row, we are pleased to be able to share the proceedings of the Annual Conference on the Science of Dissemination and Implementation in Health, a large meeting reflecting the expanding and evolving research field that seeks to optimize the use of evidence, interventions, and tools from health research within the myriad of settings where people receive health care, make health-related decisions, and increase knowledge of influences on the health of the population.

We once again benefitted from a strong partnership, co-led by AcademyHealth and the National Institutes of Health (NIH), with co-sponsorship from the Agency for Healthcare Research and Quality (AHRQ), the Patient Centered Outcomes Research Institute (PCORI), the Robert Wood Johnson Foundation (RWJF), the US Department of Veterans Affairs (VA), and the WT Grant Foundation. In addition, we benefitted from the collaboration of staff from the Centers for Disease Control and Prevention (CDC) and the World Health Organization (WHO). NIH and AcademyHealth again co-led the program planning committee, which focused on the development of the plenary sessions, and convened a scientific advisory panel to suggest speakers and advise on the overall conference development.

The planning committee identified four key areas around which to focus the plenary panels and keynote address. Dr. America Bracho, M.D., M.P.H., Executive Director of Latino Health Access in Orange County, California, spoke about the opportunities for implementation science to inform efforts to improve community health and engage underserved populations. The three plenary panels each focused on a significant future direction for dissemination and implementation (D & I) research: the interface between D&I science and population health, emerging opportunities for global implementation science, and the challenges around implementation of precision medicine. The plenary sessions were complemented by facilitated lunchtime discussions on the same three topics, which offered participants an opportunity to identify key research questions for each and brainstorm next steps. Synopses of the lunchtime discussions are included in this supplement.

Given the overwhelming success of the 2014 conference and the large number of abstracts received in 2014 (660), the program planning committee identified eight program tracks for abstract submitters to respond to, and through which the concurrent sessions of the conference would be organized. These tracks—Behavioral Health, Big Data and Technology for Dissemination and Implementation Research, Clinical Care Settings, Global Dissemination and Implementation, Promoting Health Equity and Eliminating Disparities, Health Policy Dissemination and Implementation, Prevention and Public Health, and Models, Measures and Methods— were designed to enable conference participants to follow a consistent theme across the multiple sessions of the conference and form the structure of this supplement.

The call for abstracts, including individual paper presentations, individual posters and panel presentations, resulted in 515 submissions, spread across the eight thematic tracks. Over one hundred reviewers devoted their time to ensuring a comprehensive and expert review, and reviews were conducted within each track and coordinated by the track leads. For the final program, 64 oral presentations, 12 panels, and 263 posters were presented over the two-day meeting. Slides for the oral presentations and panels (with the agreement of the authors) were posted on the conference website (http://diconference.academyhealth.org/archives/2015archives) and all abstracts were included on the conference webapp (https://academyhealth.confex.com/academyhealth/2015di/meetingapp.cgi).

This supplement has compiled the abstracts for presented papers, panel sessions, and lunchtime discussions from the 8th Annual Meeting on the Science of Dissemination and Implementation in Health: Optimizing Personal and Population Health. We are pleased to have the abstracts from the conference together in one volume once again, and look forward to the 9th Annual meeting, scheduled for December in Washington, D.C.

## Discussion forums

### D1 Discussion forum: Population health D&I research

#### Felicia Hill-Briggs

##### Department of Medicine and Welch Center for Prevention, Epidemiology and Clinical Research, Johns Hopkins Medical Institutions, Baltimore MD 21287, USA

In this lunchtime discussion forum, participants representing researchers in academia and industry, community-based organizations, public health departments, and research funders discussed opportunities and needed directions for dissemination and implementation research within population health. Population health, defined by Kindig and Stoddart as “the health outcomes of a group of individuals, including the distribution of such outcomes within the group,” [1] has emerged as a common goal engaging healthcare delivery systems, public health agencies, and communities in the era of healthcare reform under the Affordable Care Act [2].

Key research design and implementation questions identified during the forum were: Because randomized designs are often not accepted in communities, what research designs maintain scientific rigor and acceptability? What processes are needed and generalizable for translating evidence-based intervention approaches effectively into different communities and settings? With standardized metrics gaining use in research, how do researchers ensure population health outcomes that are relevant to the community stakeholders and vantage point? How can researchers have better awareness of and access to public data sources to answer population health questions or to monitor outcomes, especially economic outcomes (e.g. data repositories/data banks; partnerships with city, state health departments)? What statistical methods are needed to answer questions of population health impact most effectively, particularly detection of differential performance and responsiveness within a population (e.g. stepped wedge designs with within and between cluster comparisons, latent class methods)? What research funding is needed to further knowledge of best analytic methods (e.g. comparative utility studies of analytic methods within datasets)?

Several overarching challenges were also identified as priority for D&I researchers and funders. Key was a shift from researchers setting the research questions and applying research to communities, to researchers getting involved in communities and research emerging from the community-identified priorities. To enable this shift, changes to traditional research funding mechanisms were recommended (e.g. collaboration between funders, researchers and communities for grant restructuring that aligns research milestones and timelines with necessary community processes for D&I research). Ensuring community stakeholders are incentivized to engage in research was discussed (e.g. Co-PI structure for researcher and community partner; adequate funding for community involvement; ease and usability of IRB trainings). Finally, approaches for training the next generation of researchers for population health were deemed priority (e.g. research field placements for relationship-building in communities, training in conducting problem/needs assessments, training in analytic methods, including financial/economic).

References

[1] Kindig DA, Stoddart G. What is population health? American Journal of Public Health, 2003;93(3). p. 381.

[2] Berwick DM, Nolan TW, Whittington J. The triple aim: care, health, and cost. Health Affairs, 2008;27(3): 759-769.

### D2 Discussion forum: Global health D&I research

#### Gila Neta, Cynthia Vinson

##### Division of Cancer Control and Population Sciences, National Cancer Institute, National Institutes of Health, Rockville, MD, 20850, USA

This lunchtime discussion forum brought together researchers, funders, and practitioners to discuss issues in implementation research and delivery science in global settings. Key challenges highlighted included bringing diverse communities together and fostering collaborations among researchers, policymakers, and practitioners. Examples of implementation science projects from the World Health Organization, AcademyHealth, Harvard, University of North Carolina and the University of California, San Francisco provided concrete examples of these efforts. The group also focused on concrete opportunities to foster further discussion, including upcoming call for papers and proposals, as well as ways to embed researchers into implementation projects in real world settings and ongoing efforts.

Major questions raised within the forum included: How do we normalize the process of bringing researchers, policymakers, and practitioners together? How do we address the struggle between the researcher generating knowledge and moving to implementation and dissemination? How do you create incentives and funding opportunities? Whether or how to engage ministries of health? What are models for how policymakers set agendas and how are these implemented? How can we change the way funding mechanisms work to better support research and researchers in global settings.

Participants also raised the importance of capacity building for sustainability, and the challenge in developing capacity, including the need to develop mechanisms to keep capacity in country that is beneficial to country and trainee. Discussion also focused on the value of bidirectional learning between the U.S. and global settings, as well as the challenges of dissemination and lack of mechanisms to share what works. Participants also discussed the importance of developing tools for implementation research, and highlighted the reality that implementers may be interested but don’t have time to engage. And finally, the group discussed ways to change funding mechanisms, including changes to academic incentive structures, engaging implementers on review, and focusing funding opportunities that encourage partnering between researchers, policymakers and practitioners.

### D3 Discussion forum: Precision medicine and D&I research

#### David Chambers

##### Division of Cancer Control and Population Sciences, National Cancer Institute, National Institutes of Health, Rockville, MD, 20850, USA

This lunchtime discussion forum was intended to enable conference participants to discuss possible research directions related to the dissemination and implementation of precision medicine findings and interventions. The Precision Medicine Initiative (PMI), led by NIH and the FDA, seeks to use information on genetic, biological, behavioral and environmental factors to provide optimal health and healthcare decision-making for each person. Following a stimulus presentation by Dr. Josephine Briggs, MD, Director of the National Center for Complementary and Integrative Health, NIH, and the Acting Director of the PMI Cohort Program, participants raised questions about current activities and suggested potential directions for future work.

Major questions raised within the forum included: How do we leverage this huge cohort study to help us not only produce new knowledge, but also learn how to translate new knowledge into improvements in care? How do we avoid the delays inherent in waiting for studies to be completed and published in the peer review literature? How can we leverage broad population use of smart phones and other strategies to engage individuals (particularly those underrepresented) in the study? How do we leverage the dataset to provide context to participants in the cohort, for the relevant findings that we return to them? How do we engage with health systems to ensure relevance of studies and impact of findings to improve healthcare delivery? Can we incorporate questions about health services and health systems alongside the individual-level PMI questions likely to be asked within the cohort studies?

Participants also raised the importance of using qualitative research to better understand how precision medicine is being implemented in varied health and community settings and how findings impact decision-making, as well as whether technological changes over the course of the PMI could be studied. Opportunities to use a range of other methodologies (e.g. N-of-1 trials, observational studies of clinical encounters, adaptive designs) were also highlighted, as well as the need to understand and address study attrition, ethical dimensions of precision medicine research and practice, and approaches to address health literacy (including the more specific genetic/genomic literacy) across multiple populations.

## Behavioral health

### S1 Predictors of community therapists’ use of therapy techniques in a large public mental health system

#### Rinad Beidas^1^, Steven Marcus^2^, Gregory Aarons^3^, Kimberly Hoagwood^4^, Sonja Schoenwald^5^, Arthur Evans^6^, Matthew Hurford^6^, Ronnie Rubin^7^, Trevor Hadley^1^, Frances Barg^8^, Lucia Walsh^1^, Danielle Adams^1^, David Mandell^1^

##### ^1^Department of Psychiatry, University of Pennsylvania Perelman School of Medicine, Philadelphia, PA, 19104, USA; ^2^School of Social Policy and Practice, University of Pennsylvania, Philadelphia, PA, 19103, USA; ^3^Psychiatry, UC San Diego, La Jolla, CA, 92083-0812, USA; ^4^Child & Adolescent Psychiatry, The Child Study Center at NYU Langone Medical Center, New York, NY, New York, NY, 10016, USA; ^5^Psychiatry and Behavioral Sciences, MUSC, Charleston, SC, 29425, USA; ^6^DBHIDS, City of Philadelphia, Philadelphia, PA, 19104, USA; ^7^CBH, City of Philadelphia, Philadelphia, PA, 19104, USA; ^8^Family Medicine and Community Health, University of Pennsylvania, Philadelphia, PA, 19104, USA

###### **Correspondence:** Rinad Beidas – Psychiatry, University Of Pennsylvania, Philadelphia, PA, 19104, USA

**Background:** Few studies have examined the effects of individual and organizational characteristics on the use of evidence-based practices in mental health care. The objective of this study was to estimate the relative contribution of individual and organizational factors on therapist self-reported use of cognitive-behavioral, family, and psychodynamic therapy techniques within the context of a large-scale effort to increase use of evidence-based practices in an urban public mental health system serving youth and families.

**Methods:** In this observational, cross-sectional study of 23 organizations, data were collected from March 1 through July 25, 2013.We used purposive sampling to recruit the 29 largest child-serving agencies, which together serve approximately 80 % of youth receiving publically funded mental health care. The final sample included 19 agencies with 23 sites, 130 therapists, 36 supervisors, and 22 executive administrators. Main outcomes included therapist self-reported use of cognitive-behavioral, family, and psychodynamic therapy techniques, as measured by the Therapist Procedures Checklist–Family Revised.

**Findings:** Individual factors accounted for the following percentages of the overall variation: cognitive-behavioral therapy techniques, 16 %; family therapy techniques, 7 %; and psychodynamic therapy techniques, 20 %. Organizational factors accounted for the following percentages of the overall variation: cognitive-behavioral therapy techniques, 23 %; family therapy techniques, 19 %; and psychodynamic therapy techniques, 7 %. Older therapists and therapists with more open attitudes were more likely to endorse use of cognitive-behavioral therapy techniques, as were those in organizations that had spent fewer years participating in evidence-based practice initiatives, had more resistant cultures, and had more functional climates. Women were more likely to endorse use of family therapy techniques, as were those in organizations employing more fee-for-service staff and with more stressful climates. Therapists with more divergent attitudes and less knowledge about evidence-based practices were more likely to use psychodynamic therapy techniques.

**Implications for D&I research:** This study suggests that individual and organizational factors are important in explaining therapist behavior and use of evidence-based practices, but the relative importance varies by therapeutic technique. Organizational factors are more likely to drive use of evidence-based practices, whereas therapist attributes are more likely to drive use of non-evidence-based therapy techniques. This has important implications for both implementation and exnovation.

Primary Funding Source: National Institutes of Health - K23 MH099179.

### S2 Implementing brief cognitive behavioral therapy (CBT) in primary care: Clinicians' experiences from the field

#### Lindsey Martin^1^, Joseph Mignogna^2^, Juliette Mott^3^, Natalie Hundt^1^, Michael Kauth^1^, Mark Kunik^1^, Aanand Naik^1^, Jeffrey Cully^1^

##### ^1^Health Services Research & Development, Department of Veterans Affairs & Baylor College of Medicine, Houston, TX, 77030, USA; ^2^Treatment Core, Department of Veterans Affairs, Waco, TX, 76711, USA; ^3^National Center for PTSD, Executive Division, Department of Veterans Affairs, White River Junction, VT, 05009, USA

###### **Correspondence:** Lindsey Martin – Health Services Research & Development, Department of Veterans Affairs & Baylor College of Medicine, Houston, TX, 77030, USA

**Background:** Mental health clinicians working in primary care face unique challenges including treating patients with comorbid physical illnesses and managing environmental demands. These challenges make the use of standard length (12-16 sessions) evidence-based cognitive behavioral therapy (CBT) difficult in this setting. The objective of this study is to better understand how clinicians implemented a manualized brief (4-6 sessions) CBT intervention in their routine clinical practice.

**Methods:** Clinicians (n = 18) completed qualitative semi-structured interviews documenting their experiences using brief CBT as part of a VHA multisite hybrid effectiveness-implementation trial examining brief CBT for medically ill Veterans. The domains of the Promoting Action on Research Implementation in Health Services (PARiHS) and Reach, Effectiveness, Adoption, Implementation, Maintenance (RE-AIM) frameworks served as a priori deductive codes, while inductive coding revealed additional new findings. To glean a more in-depth understanding of how clinicians put brief CBT into practice, interview excerpts coded as ‘implementation’ were extracted and manually pile sorted to identify key themes. Coding agreement was reached through a process of negotiated consensus.

**Findings:** Our analysis identified how clinicians: (1) balanced intervention fidelity with the need to modify treatment to better align with patient needs and therapeutic styles; (2) acknowledged patients’ other significant life issues (e.g. finances, trauma histories) that fell outside the scope of the intervention; (3) involved patients in the intervention, including describing processes used to engage patients in making treatment choices; (4) managed scheduling issues and timing of treatment sessions in the primary care environment; and (5) responded to the telephone versus in-person mode of treatment delivery, noting key benefits and drawbacks.

**Implications for D&I research:** Mental health clinicians’ experiences implementing brief CBT in primary care reveal common challenges, potential solutions (e.g. freedom to develop treatment modifications and workarounds), and possible areas for refining implementation efforts (e.g. reducing the amount of session content to fit within allotted time). Qualitative methods provide a novel window into clinicians’ on-the-ground implementation practices, highlighting how their perspectives, needs and experiences are vital to consider when developing and implementing interventions that have the potential to improve the quality of mental health treatment delivered in the primary care environment.

Primary Funding Source: Department of Veterans Affairs - Department of Veterans Affairs (HSR&D grant IIR 09-088); Veterans Health Administration, Office of Research and Development; Center for Innovations in Quality, Effectiveness and Safety (CIN 13-413); SC MIRECC.

### S3 Clinician competence: Natural variation, factors affecting, and effect on patient outcomes

#### Alan McGuire^1,2^, Dominique White^2^, Tom Bartholomew^3^, John McGrew^2^, Lauren Luther^2^, Angie Rollins^2,4^, Michelle Salyers^2,5^

##### ^1^HSR&D, HSR&D Center for Health Information and Communication, Roudebush VA Medical Center, Indianapolis, IN, 46202, USA; ^2^Psychology, Indiana University Purdue University Indianapolis, Indianapolis, IN, 46202, USA; ^3^Department of Psychiatric Rehabilitation and Counseling, Rutgers University, Newark, NJ, 07107, USA; ^4^Health Services Research & Development, Richard L. Roudebush VAMC, Indianapolis, IN, 46202, USA; ^5^IUPUI Department of Psychology, ACT Center of Indiana, Indianapolis, IN, 46202, USA

###### **Correspondence:** Alan McGuire – HSR&D, HSR&D Center for Health Information and Communication, Roudebush VA Medical Center, Indianapolis, IN, 46202, USA

**Background:** Clinician competence is one aspect of program fidelity. Although much research has focused on fidelity as a primary implementation outcome, little attention has focused on competence. Moreover, extant research has been limited by use of non-representative samples of clinicians. The current study examined variation in clinician competence in providing an evidence-based illness self-management program for people with severe mental illness and its relationship with organizational factors, naturally occurring implementation supports, and consumer outcomes.

**Methods:** Program sessions from 63 clinicians, representing 21 agencies, were audio-recorded and scored using a validated measure of program competence. All members of clinicians' clinical teams were invited to complete measures of organizational readiness for chance, attitudes toward evidence-based practices, and recovery-orientation. Consumers (n = 236) reported on self-management, coping, social support, and attitudes toward substance abuse and medications at baseline and follow-up.

**Findings:** Average competence scores were in the “Needs Improvement” range; some program elements were rarely implemented. Neither organizational factors (when controlling for clinician factors) nor self-reported receipt of training or consultation were significantly related to competence. Clinician perception of their teams' training needs (Beta = -.02, S.E. = .007, d.f. = 19, t = -2.35, p = .03) and their influence on their peers (beta = .04, S.E. = .02, d.f. = 19, t = 2.75, p = .01) were associated with competence. Higher competence was associated with greater improvements in self-management (Beta = 1.56, S.E. = .63, d.f. = 37, t = 2.49, p = .02).

**Implications for D&I research:** Within a naturalistic sample, clinician competence varied widely and was associated with clinician perceptions and consumer outcomes. While some implementation outcomes may be effectively targeted via organizational level assessments and implementation supports, clinician competence may be better suited to more targeted interventions such as audit-and-feedback and supervision specific to the practice.

Primary Funding Source: National Institutes of Health - NIMH, 1R21MH096835, 1R03MH101418-01, 4R33MH096835-03, VA RR&D, D0712-W.

### S4 Exploring the multifaceted nature of sustainability in community-based prevention: A mixed-method approach

#### Brittany Cooper^1^, Angie Funaiole^2^

##### ^1^ Human Development, Washington State University, Pullman, WA, 99164, USA; ^2^ Prevention Science, Washington State University, Pullman, WA, 99164, USA

###### **Correspondence:** Brittany Cooper – Human Development, Washington State University, Pullman, WA, 99164, USA

**Background:** For prevention efforts to achieve public health impact, we need a clear understanding of the multifaceted nature of program sustainability. Existing research conceptualizes sustainability as a static, binary endpoint and few studies use psychometrically validated instruments, particularly in diverse community settings.

**Methods:** This mixed-method study explores the community, organizational, and program factors associated with sustainability in a sample of Strengthening Families Programs (an evidence-based, family-focused, youth substance use prevention program) implemented under natural conditions as part of the 10-year dissemination effort in Washington. Sixty facilitators completed an online survey, including the Program Sustainability Index (PSI; Mancini & Marek, 2004) and Program Sustainability Assessment Tool (PSAT; Luke et al., 2014). They also reported sustainability based on Pluye et al.’s (2004) four levels: 1) absence (no activity is continued), 2) precarious (some activities are pursued unofficially), 3) weak (some official activities that are not routinized), and 4) routinized.

**Findings:** Analyses indicate good internal consistency (α = .82-.89 for four of five PSI scales, .88-.97 for all PSAT scales) and predictive validity. Three of five PSI scales were positively related with sustainability: leadership (*r* = .49, *p* < .001), collaboration (*r* = .47, *p* < .01), and funding (*r* = .58, *p* < .0001). All but one PSAT scale were positively related with sustainability: environmental support (*r* = .69, *p* < .0001), funding stability (*r* = .62, *p* < .0001), partnerships (*r* = .61, *p* < .0001), organizational capacity (*r* = .61, *p* < .0001), program evaluation (*r* = .40, *p* < .01), communication (*r* = .62, *p* < .0001), and strategic planning (*r* = .45, *p* < .01). Multiple regression analyses were run to examine factors that were uniquely associated with sustainability. For PSI, only funding, and for PSAT, only strategic planning and environmental support were associated with sustainability, after accounting for the other factors. Additional analyses will assess the validity of these results with themes identified from semi-structured interviews with 14 sites.

**Implications for D&I research:** This study helps unpack the conditions needed to promote long-term sustainability of community-based prevention programs and ultimately improve population health.

Primary Funding Source: Washington State University Faculty Pilot Grant Support Program for Research on Alcohol and Drug Abuse.

### S5 Theory informed behavioral health integration in primary care: Mixed methods evaluation of the implementation of routine depression and alcohol screening and assessment

#### Julie Richards^1^, Amy Lee^1^, Gwen Lapham^1^, Ryan Caldeiro^2^, Paula Lozano^3^, Tory Gildred^2^, Carol Achtmeyer^4^, Evette Ludman^1^, Megan Addis^1^, Larry Marx^2^, Katharine Bradley^1^

##### ^1^Group Health Research Institute, Group Health, Seattle, WA, 98101, USA; ^2^Behavioral Health Services, Group Health, Seattle, WA, 98101, USA; ^3^Preventive Care, Group Health, Seattle, WA, 98101, USA; ^4^Health Services Research & Development; Primary and Specialty Medical Care Service, VA Puget Sound, Seattle, WA, 98108, USA

###### **Correspondence:** Julie Richards – Group Health Research Institute, Group Health, Seattle, WA, 98101, USA

**Background:** The US Preventive Services Task Force recommends routine alcohol and depression screening and follow-up in primary care (PC). However, sustained implementation of routine depression and alcohol care is uncommon in PC. This mixed-methods study evaluated a multi-pronged strategy to implement routine screening and assessment for depression and alcohol misuse in a large PC clinic.

**Methods:** The Greenhalgh Model for diffusion of innovations informed the implementation strategy for screening and assessment of depression and alcohol misuse. The strategy relied on existing electronic health records and quality improvement processes *(Infrastructure* and *Readiness*) influenced by HEDIS measures (*Outer Context*). Components of the implementation strategy were focused on: the *Linkage* between clinical operations (*User System*) and a leadership team (comprised of *Resources*, *Change Agents* and *Experts*), with a practice coach and project manager; and frontline staff (*Adopter*) attitudes and skills, with training and support for local clinical champions, a ‘design event’ for frontline staff to develop process improvement, and mixed-media patient education materials designed to subtly address stigma and shift staff attitudes. Mixed-methods were used to evaluate the active phase of implementation (February-August 2015). Quantitative analyses focused on the reach of screening and diagnostic assessment for major depression and alcohol misuse. Formative evaluation assessed determinants of implementation.

**Findings:** Prior to implementation, the prevalence of depression and alcohol screening among 4,967 adult PC patients with visits in January 2015 were 35 % and 13 %, respectively. During active implementation, 81 % of 12,998 adult PC patients completed depression and alcohol screening. Of those screening positive, 69 % were assessed for depression and suicidal ideation and 58 % for alcohol use disorder (AUD). Key implementation facilitators included: positive stories to spread enthusiasm for the work, ownership of the screening process by Medical Assistants, perceived value of the diagnostic assessments by clinicians, and prior training clinic social workers received for engagement of patients with AUD. Key barriers included other clinic reorganization and initial distrust of performance metrics by frontline staff.

**Implications for D&I research:** Routine screening and assessment for depression and alcohol misuse was successfully implemented by addressing the complex determinants of innovation diffusion in healthcare systems. The strategy is being refined to spread to 24 other clinics.

Primary Funding Source: Agency for Healthcare Research and Quality - This work was supported by a grant from the Agency for Healthcare Research and Quality (1R18HS023173-01).

### S6 Enhancing the evidence for specialty mental health probation through a hybrid efficacy and implementation study

#### Tonya VanDeinse^1^, Amy Blank Wilson^1^, Burgin Stacey^1^, Byron Powell^2^, Alicia Bunger^3^, Gary Cuddeback^1^

##### ^1^School of Social Work, University of North Carolina at Chapel Hill, Chapel Hill, NC, 27599, USA; ^2^Health Policy & Management, The University of North Carolina at Chapel Hill, Chapel Hill, NC, 27566-7411, USA; ^3^College of Social Work, Ohio State University, Columbus, OH, 43210, USA

###### **Correspondence:** Tonya VanDeinse – School of Social Work, University of North Carolina at Chapel Hill, Chapel Hill, NC, 27599, USA

**Background:** Specialty mental health probation (SMHP) has been disseminated widely to improve mental health and criminal justice outcomes among probationers with mental illness; however, the evidence for its efficacy is promising but limited. Moreover, little is known about the factors that facilitate or inhibit the implementation of SMHP and there is limited information about the strategies used to implement SMHP. To address these gaps, concurrent with a multi-site randomized efficacy trial, we are conducting an implementation study to gather information about SMHP implementation barriers, facilitators, and strategies. Here, we report our findings from the initial implementation phase of the study. We believe this is the first study to examine implementation strategies and efficacy of SMHP using a hybrid implementation – RCT design.

**Methods:** Semi-structured interviews about perceived barriers and facilitators experienced during the implementation process were conducted with 26 stakeholders, including representatives from mental health, criminal justice and the implementation and research team from a local university. We used open coding techniques during the initial phase of implementation and inductively analyzed SMHP implementation facilitators and barriers, which were later deductively coded using the Consolidated Framework for Implementation Research (CFIR). Then, we identified and specified implementation strategies in accordance with the Expert Recommendations for Implementing Change (ERIC).

**Findings:** Barriers to implementing SMHP were primarily associated with two CFIR constructs: inner setting (e.g., readiness for implementation, networks and communication) and intervention characteristics (e.g., complexity, cost). Implementation facilitators were largely related to constructs associated with the outer setting (e.g., cosmopolitanism, external change agents) and process (e.g., executing). Several implementation strategies were identified, including: identifying and preparing champions, mandating change, and building a coalition.

**Implications for D&I research:** This study optimizes the reach of efficacy trials within criminal justice settings using a hybrid efficacy and implementation design and advances our understanding of the barriers, facilitators, and strategies associated with the implementation of mental health interventions within probation settings, an area about which little is known. Our findings promise to have broad practice and policy implications for mental health and criminal justice authorities at local, state and national levels.

Primary Funding Source: Other (please specify below) - Grant from the NC Governor's Crime Commission.

### S7 Personalizing evidence-based child mental health care within a fiscally mandated policy reform

#### Miya Barnett^1^, Nicole Stadnick^2^, Lauren Brookman-Frazee^2^, Anna Lau^1^

##### ^1^Psychology, University of California, Los Angeles, Los Angeles, CA, 90095, USA; ^2^Psychiatry, University of California, San Diego, La Jolla, CA, 92093, USA

###### **Correspondence:** Miya Barnett – Psychology, University of California, Los Angeles, Los Angeles, CA, 90095, USA

**Background:** Select evidence-based practices (EBPs) for children’s mental health (MH) have been culturally adapted, but public health impact of these efforts is limited due to challenges disseminating practices for diverse groups (Cabassa & Baumann, 2013). A promising approach to increasing the impact of EBPs on the broader population is increased attention to strategies community clinicians use to personalize practices. In 2009, Los Angeles County launched the Prevention and Early Intervention (PEI) Transformation. Within this MH system reform, multiple EBPs were mandated through reimbursement practices, with initial implementation support provided for six practices. This large-scale endeavor provides a unique opportunity to characterize adaptations clinicians use to improve engagement for ethnically diverse families across practices.

**Methods:** This sequential mixed-methods study examined therapist-reported clinical adaptations to six practices for children receiving MH services within the PEI Transformation. Quantitative data were drawn from 780 clinicians who completed a survey regarding the degree to which they tailored a practice. Follow-up clinician interviews were conducted to complement and expand quantitative survey data to characterize the nature and purpose of adaptations for culturally diverse groups.

**Findings:** Clinician survey data indicated that, on average, 90 % of their caseloads comprised ethnic minorities. Across practices, 96-100 % of clinicians reported adapting a practice. Survey data and interviews were complementary with clinicians frequently reporting that they modified language when discussing clinical components, integrated supplemental clinical strategies or services, and extended duration of treatment. Adaptations infrequently reported were removing clinical components, shortening treatment length, and adjusting the order in which clinical components were delivered. Qualitative themes explained that clinicians changed language and extended treatment length to increase comprehension for cultural groups with low MH literacy. Overall, practices were viewed as flexible to accommodate minor adaptations to promote cultural relevance.

**Implications for D&I research:** Understanding community clinicians’ adaptations to personalize EBPs for clients is important in efforts to implement these practices in routine care settings and maximize practice fit with the client population served. Our findings indicate that clinical adaptations are common and appear to be in line with suggestions from researchers to promote practice-client fit, particularly for culturally diverse groups (Lau, 2006).

Primary Funding Source: National Institutes of Health - NIMH R01 MH100134 (MPI: Lau & Brookman-Frazee).

### S8 Leveraging an existing resource for technical assistance: Community-based supervisors in public mental health

#### Shannon Dorsey^1^, Michael Pullmann^1,2^

##### ^1^Psychology, University of Washington, Seattle, WA, 98195, USA; ^2^Department of Psychiatry and Behavioral Sciences, Division of Public Behavioral Health, University of Washington, Seattle, WA, 98102, USA

###### **Correspondence:** Shannon Dorsey –Psychology, University of Washington, Seattle, WA, 98195, USA

**Background:** Existing community-based supervisors (CBSs) are an underutilized resource for supporting scale up of evidence-based treatment (EBT) in public mental health settings. Most providers have supervision; but very few EBT utilize CBS, who offer an efficient and affordable mechanism.

**Methods:** We present data from a study of CBS involved in a state-supported EBT effort (NIH = funded). This study provides the only examination, to our knowledge, of EBT-trained CBSs use supervision time. Supervisors (N = 56) and clinicians (N = 205) reported on how supervision time was spent, across nine different areas (clinical and non-clinical, EBT and general focus).

**Findings:** Supervisors (N = 56) and clinicians (N = 205) report a high concordance of time spent on a variety of wide-ranging supervision topics. Time spent on two EBT-critical activities,—case conceptualization and treatment intervention—was less than half of the supervision. Variance in how *much* time was spent on these activities, surprisingly clustered at the level (ICC = .318, -2 L-D χ^2^ = 18.3, *p* < .001, AIC-D χ^2^ = 16.3, *p* < .001), but not the organizational (ICC = .183, -2 L-D χ^2^ = 13.4, *p* < .001, AIC-D χ^2^ = 22.3, *p* < .004), with substantial variation by supervisor within organizations. Notably, both supervisors (76.2 %) and clinicians (74.8 %) overwhelmingly reported a desire for more time on case conceptualization and treatment intervention.

**Implications for D&I research:** To achieve population-level improvement, already funded resources are critical. We discuss implications of multiple demands on CBS for EBT implementation and present preliminary findings from a RCT designed to better integrate these two activities into supervision.

Primary Funding Source: National Institutes of Health - R01MH095749.

### S9 SBIRT implementation for adolescents in urban federally qualified health centers: Implementation outcomes

#### Shannon Mitchell^1^, Robert Schwartz^1^, Arethusa Kirk^2^, Kristi Dusek^1^, Marla Oros^3^, Colleen Hosler^3^, Jan Gryczynski^1^, Carolina Barbosa^4^, Laura Dunlap^5^, David Lounsbury^6,7^, Kevin O'Grady^8^, Barry Brown^9^

##### ^1^Social Research Center, Friends Research Institute, Baltimore, MD, 21201, USA; ^2^Pediatrics, Total Health Care, Baltimore, MD, 21217, USA; ^3^The Mosaic Group, The Mosaic Group, Baltimore, MD, 21210, USA; ^4^Behavioral Health Economics Program, RTI International, Chicago, IL, 60606-4901, USA; ^5^Center for Interdisciplinary Substance Abuse Research, Research Triangle Institute, Rockville, MD, 20852, USA; ^6^Epidemiology and Population Health, Albert Einstein College of Medicine, Bronx, NY, 10467, USA; ^7^Division of Community Collaboration and Implementation Science, Albert Einstein College of Medicine of Yeshiva University, Bronx, NY, 10467, USA; ^8^Department of Psychology, University of Maryland, College Park, College Park, MD, 20742, USA; ^9^Psychology, University of North Carolina at Wilmington, Wilmington, NC, 28403, USA

###### **Correspondence:** Shannon Mitchell – Social Research Center, Friends Research Institute, Baltimore, MD, 21201, USA

**Background:** Alcohol, tobacco, and other drug use remains highly prevalent among US adolescents and is a threat to their well-being and to the public health. Despite evidence of the effectiveness of Screening, Brief Intervention and Referral to Treatment (SBIRT), and support by American Academy of Pediatrics, primary care providers have been slow to adopt this evidence-based approach. Thus, research is needed to determine effective ways to implement SBIRT for adolescent substance misuse in primary care settings.

**Methods:** This cluster randomized trial (*N* = 7) compared two principal approaches to SBIRT delivery within adolescent medicine: Generalist *vs.* Specialist. In the Generalist Approach, the primary care provider delivers brief intervention (BI) for substance misuse. In the Specialist Approach, BIs are delivered by co-located behavioral health counselors. Multilevel logistic regression modeling was used to examine differences by Condition in rates of successful delivery and documentation of the following services: (a) screening of adolescent patients, (b) brief advice (for patients reporting low levels of alcohol or drug use), and (c) brief intervention (for patients reporting moderate to high levels of drug or alcohol use).

**Findings:** Multilevel logistic regression analyses taking into account the cluster-randomized design showed no significant differences between Generalist and Specialist conditions in penetration of screening (p = .52) or brief advice (p = .77). The Generalist Condition had significantly higher penetration of brief intervention delivered than the Specialist Condition (p = .005). There were significant time period differences in screening (above and beyond differences by Site and Condition), but not for brief advice or brief intervention. Site-level intraclass correlations were high and there was significant variation by Site in penetration of screening, brief advice, and brief intervention.

**Implications for D&I research:** Despite having complementary, co-located specialized services, such as behavioral health, within primary care settings, the implementation of a Specialist approach to providing adolescent SBIRT services was less effectively implemented than the more straight-forward Generalist approach. However, youth with drug, alcohol, or tobacco misuse may benefit from receiving a hand-off from their primary care provider to a behavioral health counselors to address social and emotional issues tied to their drug or alcohol misuse.

Primary Funding Source: National Institutes of Health - National Institute on Drug Abuse grant 1R01DA034258-04.

### S10 PANEL: Tailoring Implementation Strategies to Context - Expert recommendations for tailoring strategies to context

#### Laura Damschroder^1^, Thomas Waltz^1,2^, Byron Powell^3^

##### ^1^VA Center for Clinical Management Research, Department of Veterans Affairs, Ann Arbor, MI, 48105, USA; ^2^Psychology, Eastern Michigan University, Ypsilanti, MI, 48197, USA; ^3^Health Policy & Management, The University of North Carolina at Chapel Hill, Chapel Hill, NC, 27566, USA

###### **Correspondence:** Laura Damschroder – VA Center for Clinical Management Research, Department of Veterans Affairs, Ann Arbor, MI, 48105, USA

**Background:** Implementation researchers and practitioners need further guidance about which implementation strategy to use under varying circumstances. To address this pressing need, an international roster of implementation experts was asked to select and rank strategies that would best address various contextual barriers. Implementation strategies were presented based on the list developed through the Expert Recommendations for Implementing Change (ERIC) project, where experts contributed to developing a comprehensive taxonomy of implementation strategies. Scenarios describing contextual barriers were developed based on the Consolidated Framework for Implementation Research (CFIR), which provides a set of 39 theoretical constructs (e.g., Leadership Engagement), within five domains (e.g., Inner Setting) that are believed to influence implementation effectiveness. Survey results, thus, provide a mapping of ERIC discrete strategies to CFIR contextual barriers.

**Methods:** Participants were recruited from an international list of over 430 implementation researchers and practitioners. Participants were randomly assigned a contextual barrier based on the CFIR and asked to select and rank up to 7 ERIC strategies they believed to best address that barrier. The barriers were presented in random order and participants could provide responses to as many barriers as they wished.

**Findings:** Over 100 participants engaged in the sorting and rating tasks for an average of 6 barriers each. Summary data will be presented showing which strategies experts thought would best address barriers - the number of recommendations each strategy received for addressing each barrier along with their average rank.

Implications for D&I Research: The map reflects the most frequently endorsed implementation strategies for common implementation barriers. Results will inform development of a tool that can be used to select strategies recommended to address barriers identified through context assessment using the CFIR. This tool will be freely available and posted online (see http://cfirguide.org/techniques.html). This mapping is a first step in providing expert recommendations for tailoring implementation strategies to context. Importantly, these results yield hypotheses researchers can use to empirically validate and improve upon these recommendations.

Primary Funding Source: Department of Veterans Affairs - QLP 92-025; QUERI-DM, DIB 98-001

### S11 PANEL: Tailoring Implementation Strategies to Context - Extreme facilitation: Helping challenged healthcare settings implement complex programs

#### Mona Ritchie

##### VA QUERI Program for Team-Based Behavioral Health, Department of Veterans Affairs, North Little Rock, AR, 72114, USA

**Background:** Facilitation is a widely used and promising strategy for implementing evidence-based approaches in clinical settings. We tested a multi-faceted implementation strategy that included facilitation within the context of a VA initiative to integrate mental health services in primary care. An external facilitator with expertise in implementation science and integrated care worked with and mentored an internal regional facilitator in 2 VA networks. Implementation science theory and empirical evidence informed their efforts. We conducted the study in 8 primary care clinics that would be unlikely to implement integrated care programs without assistance. Clinics receiving implementation facilitation showed higher program reach, adoption, implementation, quality and adherence to evidence than comparison clinics. This presentation will describe challenges facilitators encountered and implementation strategies they applied.

**Methods:** We conducted monthly debriefing interviews with facilitators over a 30-month period to document their activities and organizational contexts of clinics receiving implementation facilitation. We also created site summary notes to enable exploration of implementation determinants such as leadership and programmatic support, staff issues, organizational structure, implementation resources, and facilitator perceived barriers.

**Findings:** Study sites experienced a wide variety of implementation challenges, e.g., limited leadership buy-in and support, limited understanding of integrated care, its value, and the need to implement it, lack of implementation resources, competing demands, and staff turnover. Throughout the process of working with sites, facilitators assessed individuals and context, as well as implementation processes, progress and outcomes. Based on this, they selected and applied over half of the 73 discrete implementation strategies identified by the Expert Recommendations for Implementing Change (ERIC) project, tailoring them to site context, need and existing resources.

**Implications for D&I research**: Implementation facilitators of complex programs in challenged healthcare settings need to know how and when to apply many discrete implementation strategies. Understanding which strategies might address particular challenges would be useful for planning and executing implementation efforts and training new facilitators. The external facilitator in our study successfully transferred evidence-informed implementation facilitation skills that incorporated many other strategies. It is possible that tools, i.e., those developed by the ERIC project, could shorten this process.

Primary Funding Source: Department of Veterans Affairs - SDP 08-316.

### S12 PANEL: Tailoring Implementation Strategies to Context - Using menu-based choice tasks to obtain expert recommendations for implementing three high-priority practices in the VA

#### Thomas Waltz^1,2^

##### ^1^Psychology, Eastern Michigan University, Ypsilanti, MI, 48197, USA; ^2^Health Services Research & Development, VA Center for Clinical Management Research, Ann Arbor, MI, 48105, USA

**Background:** The Expert Recommendations for Implementing Change (ERIC) project had two aims: to establish consensus on a common nomenclature for implementation strategy terms and to develop recommendations specifying which of these strategies are relevant for integrating each of three high priority Veterans Administration (VA) mental health practices: metabolic monitoring for patients taking antipsychotics, measurement-based care for depression, and prolonged exposure therapy for posttraumatic stress disorder. Aim 1 activities produced a compilation of 73 discrete implementation strategies. This presentation focuses on Aim 2 activities where expert panelists were tasked with determining which of the strategies from the ERIC compilation were considered essential for supporting implementation of a particular mental health practice.

**Methods:** Using menu-based choice (MBC) methods, panelists were asked to make recommendations while considering the combination of multiple elements: the mental health practice, site level context variations, and the stage of implementation: pre-implementation, active implementation, and sustainment). Panels of 20 or more experts provided structured recommendations for each of the three practice changes across multiple possible combinations of the above elements, indicating whether each of 73 strategies was absolutely essential, likely essential, likely inessential, or absolutely inessential across multiple assessment points.

**Findings:** Recommendations derived from the MBC data varied across the mental health practices, contextual variations and stages of implementation. No strategies were universally recommended as absolutely essential across target practices and contextual variations, although some (e.g., assess for readiness and identify barriers and facilitators, tailor strategies) were rated as absolutely essential across most. Only one strategy was universally rated as absolutely inessential given these target practices: change liability laws.

**Implications for D&I research:** This project is an essential early step in empirically matching particular implementation strategies to certain practices under varying circumstances. Discussion will focus on interpreting the results and characterizing consensus for strategies given the responses the expert panel provided. Also, this project showed that menu-based choice is a promising tool for structuring complex expert recommendations tasks. Additional data analytic opportunities made possible by MBC, such as characterizing strategies that serve as complements or substitutes for one another will also be discussed.

Primary Funding Source: Department of Veterans Affairs - QLP 55-025.

## Big data & technology for dissemination & implementation research

### S13 PANEL: The Use of Technology to Improve Efficient Monitoring of Implementation of Evidence-based Programs - Siri, rate my therapist: Using technology to automate fidelity ratings of motivational interviewing

#### David Atkins^1^, Zac E. Imel^2^, Bo Xiao^3^, Doğan Can^4^, Panayiotis Georgiou^3^, Shrikanth Narayanan^5^

##### ^1^Department of Psychiatry and Behavioral Sciences, University of Washington, Seattle, WA, 98105, USA; ^2^Educational Psychology, University of Utah, Salt Lake City, UT, 84112, USA; ^3^Electrical Engineering, University of Southern California, Los Angeles, CA, 90089, USA; ^4^Computer Science, University of Southern California, Los Angeles, CA, 90089, USA; ^5^Electrical Engineering & Computer Science, University of Southern California, Los Angeles, CA, 90089, USA

###### **Correspondence:** David Atkins – Department of Psychiatry and Behavioral Sciences, University of Washington, Seattle, WA, 98105, USA

**Background:** Behavioral interventions such as psychotherapy are challenging to assess and quantify. The dyadic interaction of client and therapist includes *semantic information* in the specific words that are used, *paralinguistic information* in the tone and prosody of how words are spoken, *nonverbal information* in the gesture and posture of both individuals, and the dynamic interdependence of these as they unfold over time. Although behavioral coding is considered the gold-standard for assessing treatment fidelity, the reliance on humans as the assessment tool is labor intensive, time consuming, can lead to problematic reliability, and for all these reasons, human coding does not scale up for use in real-world settings. The current presentation will introduce tools and models from behavioral signal processing (BSP) for automating the estimation of treatment fidelity in motivational interviewing (MI). BSP emerged from the signal processing area of electrical engineering and focuses on modeling human behavior from multi-modal signal inputs, such as speech, language, gestures, and physiology.

**Methods:** Key BSP methodologies to take an audio recorded Motivational Interviewing (MI) session as input and produce computational-based fidelity ratings include: voice activity detection (*is* someone speaking?), diarization and role detection (*which* speaker is speaking?), and automated speech recognition (ASR; decoding the acoustic signal into words). The ASR text and speech features are then used as inputs into machine learning models with human-rated MI fidelity scores as the outcome. Recordings come from 356 MI sessions from several randomized trials and an MI training study based in community addiction clinics.

**Findings:** The accuracy of computational-based MI fidelity ratings varied notably by the specific code, where codes tied more closely to common dialogue acts (e.g., questions, reflections) are competitive with human ratings and reliability, whereas more abstract codes (e.g., client ‘change talk’) were notably less accurate relative to humans. As one specific example, the model achieved 86 % classification accuracy with rating therapist empathy.

**Implications for D&I research:** Research has already demonstrated that feedback based on MI fidelity codes can help train and maintain treatment quality. The current research provides a computational pathway for scaling up treatment fidelity ratings to large-scale, real-world delivery.

Primary Funding Source: National Institutes of Health - This work was supported by NIDA (R34 DA034860) and NIAAA (R01 AA018673, K02 AA023814).

### S14 PANEL: The Use of Technology to Improve Efficient Monitoring of Implementation of Evidence-based Programs - Identifying indicators of implementation quality for computer-based ratings

#### Cady Berkel^1^, Carlos Gallo^2^, Irwin Sandler^1^, C. Hendricks Brown^2^, Sharlene Wolchik^1^, Anne Marie Mauricio^3^

##### ^1^REACH Institute, Arizona State University, Tempe, AZ, 85284, USA; ^2^Center for Prevention Implementation Methodology, Northwestern University, Chicago, IL, 60611, USA; ^3^Psychology, Arizona State University, Tempe, AZ, 85287, USA

###### **Correspondence:** Cady Berkel – REACH Institute, Arizona State University, Tempe, AZ, 85284, USA

**Background:** Effect sizes of Evidence-Based Programs (EBPs) often decrease as they are disseminated in community settings, largely due to declines in implementation. Efficient and effective measures for monitoring implementation in community settings are thus a critical precursor to widespread dissemination of high quality programs. Objective coding by independent observers are the current gold standard in assessing implementation. However, this method is expensive, inefficient, and beyond the capacity of most community organizations. New developments in computer-based ratings have made it possible to overcome these limitations. The success of these endeavors will depend on choosing indicators that are suitable for computer ratings. A suitable indicator should meet the following principles: be relevant to the program’s theory; have evidence of human interrater reliability (IRR); account for variance in the overall dimension of implementation; vary across the sample; be operationalizable; have validity in predicting program outcomes; be burdensome for human coding. Data from the New Beginnings Program (NBP) will be used to demonstrate these principles.

**Methods:** The NBP is a group-format EBP for divorcing families that has been adapted for widescale delivery and tested in an effectiveness trial across the state of Arizona. Independent observers rated implementation quality in 500 activities (one for each of the 10 sessions for all 50 groups) using the Hi-Q. Implementation quality has been related to program outcomes in multiple studies and involves judgements about both the content of what is said and the emotional tone. The Hi-Q has 25 items rated on a 1-5 scale. For IRR, 107 activities were coded by multiple raters.

**Findings:** Data will be presented demonstrating how Hi-Q items fit each of the principles for successful computer-based rating of implementation. For example, ICCs indicate most items were reliable across human coders, ranging from .90 to .54 (M = .68). Two items were in the “excellent” range, 21 in the “good” range, and two in the “fair” range. As evidence of item variability, full range of the scale was used for 22 items; the average item mean was 3.3 (SD = .80).

**Implications for D&I Research:** The principles and examples presented will assist attendees in identifying efficient and effective methods of monitoring implementation through the use of innovative computer-based technology.

Primary Funding Source: National Institutes of Health - This work was supported by NIDA (R01DA026874 and R01DA033991).

### S15 PANEL: The Use of Technology to Improve Efficient Monitoring of Implementation of Evidence-based Programs - Improving implementation of behavioral interventions by monitoring emotion in spoken speech

#### Carlos Gallo^1^, C. Hendricks Brown^1^, Sanjay Mehrotra^2^

##### ^1^Center for Prevention Implementation Methodology, Northwestern University, Chicago, IL, 60611, USA; ^2^Industrial Engineering, Northwestern University, Chicago, IL, 60611, USA

###### **Correspondence:** Carlos Gallo – Center for Prevention Implementation Methodology, Northwestern University, Chicago, IL, 60611, USA

**Background:** Traditional methods for monitoring implementation require expensive, labor intensive observations by highly trained professionals. This is often impractical and prohibitive for local agencies who implement evidence based programs (EBPs). There is initial evidence that automatic, computer-based methods can help reduce the burden of monitoring implementation. These methods require linguistic and nonlinguistic processing of the speech signal recorded during the behavioral intervention delivery.

**Methods:** We are reporting on new findings from data consisting of 540 audio clips from the Linguistic Data Consortium speech database. We 1) apply seven principles for identifying para-linguistic constructs operationalizable by machine (e.g., recognition of emotion), and 2) develop computational methods for monitoring implementation constructs such as emotion. These methods rely on machine classification techniques.

**Findings:** Quantitative assessments of reliability against human coding are provided. Machine methods classify neutral from emotional speech with a high success rate (Kappa: .79). Performance is lowered when classifying three or more emotions.

**Implications for D&I Research:** These methods for monitoring fidelity can potentially 1) reduce the cost of implementation monitoring, 2) increase the number of sessions (potentially 100 %) for which implementation can be monitored and 3) facilitate supervision and provision of feedback to maintain fidelity and quality of implementation by local agencies. These methods have the potential to advance the development of systems to maintain high quality implementation of EBP when delivered in community settings.

Primary Funding Source: National Institutes of Health - Support for this research was provided by the Center for Prevention Implementation Methodology P30 DA027828 (NIDA), as well as the Diversity Supplement to Carlos Gallo by the National Institute on Drug Abuse (NIDA) on R01DA033991-02 (Berkel & Mauricio PI).

### S16 Scorecards and dashboards to assure data quality of health management information system (HMIS) using R

#### Dharmendra Chandurkar^1^, Siddhartha Bora^1^, Arup Das^2^, Anand Tripathi^2^, Niranjan Saggurti^3^, Anita Raj^4^

##### ^1^Research, Sambodhi Research and Communications Private Limited, Noida, 201301, India; ^2^Monitoring and Evaluation, India Health Action Trust, Lucknow, 226001, India; ^3^Monitoring, Learning and Evaluation, Bill and Melinda Gates Foundation, New Delhi, 110057, India; ^4^Division of Global Public Health, Department of Medicine, University of California, San Diego, San Diego, CA, 92093, USA

###### **Correspondence:** Dharmendra Chandurkar – Research, Sambodhi Research and Communications Private Limited, Noida, 201301, India

**Background:** Addressing the need for reliable informating in shaping public health policies, countries across Africa and Asia have implemented large scale HMIS for planning, monitoring and evaluation. HMIS was launched in India in 2008, however, data quality has remained largely unreliable; as is the case in other countries. While studies have underlined this problem, they have been of limited use to program managers falling short of insights into the nature, origin of errors or geographic phenomenon. We present a methodology to track data quality using scorecards and dashboards for pinpointing specific issues for corrective actions.

**Methods:** Using R, HMIS data of 7641 facilities across 25 districts of Uttar Pradesh, India for 2014-15 is analysed in four domains. **Completeness** and **uniqueness** are calculated as the percentage of missing entries and duplicate entries. **Accuracy** is percent observations passing specific logical rules. Moving averages measure **consistency** with previously reported data. Composite scorecards are developed depicting quarterly error rates. Analysis is done with respect to reporting units (health facilities) at district and sub-district levels and geo-referenced for developing dashboards.

**Findings:** There is a decrease of 2-5 % in composite error rates across various levels of reporting. Only for the lowest level of reporting there is a decrease of 5 % in proportion of facilities reporting >30 % missing values. At sub-district level, there is reduction of 6-10 % in reporting of invalid values. However, inconsistency remained the same at all the levels with 3-10 % increase in proportion of facilities reporting duplicate entries. Three districts get highlighted as those with persistent quality issues.

Implications for D&I Research: Scorecards and dashboards are effective tools for program managers to address data quality issues and trigger a ‘virtuous cycle’ of improved data quality for improved data use in decision-making. The methodology enables identification of specific units with high error rates, contributing factors and extent of phenomenon in a geography such that specific support can be provided for redressal. In view of the commonality of issues faced in data quality in other states and globally, the method could be easily adopted at scale. The methodology is highly adaptable and can be easily replicated in countries with similar contexts and issues.

Primary Funding Source: Bill and Melinda Gates Foundation.

### S17 A big data approach for discovering and implementing patient safety insights

#### Eric Hughes^1^, Brian Jacobs^2^, Eric Kirkendall^3^

##### ^1^Information Technology, MITRE, Bedford, MA, 01730, USA; ^2^VP, CMIO, CIO, Children’s National Health System, Washington, DC, 20010, USA; ^3^Assoc. CMIO, Cincinnati Children’s Hospital and Medical Center, Cincinnati, OH, 45229, USA

###### **Correspondence:** Eric Hughes – Information Technology, MITRE, Bedford, MA, 01730, USA

**Background:** The National Patient Safety Partnership (NPSP) was formed between Boston Children’s Hospital, Children’s National Medical Center, Cincinnati Children’s Hospital Medical Center, and MITRE, to “take patient safety to the next level”. Big data analytics has been successfully applied to other complex problems, yet most healthcare organizations lack experience with it.

**Methods:** NPSP identifies insights and improves patient safety with big data:

- Conduct studies with data scientists, clinicians, safety officers and IT experts
- Fuse data from disparate EHRs in rapid cycles to identify patterns and trends, and iteratively focus on data-driven insights
- Use descriptive statistics to guide each study, rather than choosing specific hypotheses up front
- Evaluate results to determine how to best operationalize

**Findings:** Multiple new insights have been gained in just 2 years using big data analysis – all have been partially implemented or are in the process of being piloted. The Patient Deterioration study uses many types of data to develop a model that accurately (>80 % precision) predicts whether a patient is at high risk to critically deteriorate. NPSP experimented with thousands of analytic features, with continuous performance evaluation. One hospital plans to pilot the model in Fall 2015. The Alarms study looked at patterns in alarms from physiologic monitors across many different units and patient types for one hospital, with a focused pilot for one unit resulting in fewer spurious alarms and fewer monitored patients. The hospital is currently evaluating wider deployment. The Medication Safety study created a breakthrough common data model across EHR systems. The study examined safety issues with dose changes for high-risk medications, and identified costly potential waste and inefficiency that are being considered for interventions.

**Implications for D&I Research:** There exists a dichotomy between traditional research methods, which examine narrow hypotheses in great depth, and quality improvement methods, which explore broad-ranging phenomena usually within a single setting. NPSP represents a novel, middle ground that explores broad phenomena in greater depth, across multiple settings. Pediatric safety leaders believe the approach can greatly improve the likelihood of implementation and dissemination of results by engaging relevant stakeholders throughout, and testing concepts across different workflows.

Primary Funding Source: MITRE internal funding.

### S18 Improving the efficacy of a depression registry for use in a collaborative care model

#### Danielle Loeb^1^, Katy Trinkley^2^, Michael Yang^3^, Andrew Sprowell^3^, Donald Nease^4^

##### ^1^Department of Medicine/Division of General Internal Medicine, University of Colorado School of Medicine, Aurora, CO, 80045, USA; ^2^Department of Clinical Pharmacy, University of Colorado School of Pharmacy, Aurora, CO, 80045, USA; ^3^Medical Student, University of Colorado School of Medicine, Aurora, CO, 80045, USA; ^4^Department of Family Medicine, University of Colorado School of Medicine, Aurora, CO, 80238, USA

###### **Correspondence:** Danielle Loeb – Department of Medicine/Division of General Internal Medicine, University of Colorado School of Medicine, Aurora, CO, 80045, USA

**Background:** Efficient population-based chronic disease management depends on functioning patient registries. Two internal medicine clinics initiated a universal depression screening protocol in 2013 using the Patient Health Questionnaire (PHQ)-2 for initial screening and the PHQ-9 for tracking of patients diagnosed with depression. To improve the treatment of depression, the clinic decided to implement a Collaborative Care Model (CCM). In preparation for the CCM intervention, we created a registry of patients with depression. Our primary objective was to develop a systematic process to identify and track patients with depression.

**Methods:** We utilized the RE-AIM implementation model to design the registry in two Mountain West academic outpatient internal medicine clinics with an Electronic Health Record (EHR). It was built to measure the reach, effectiveness, and adoption of the planed CCM. The initial database included patients with either a diagnosis of depression or dysthymia or a PHQ-9 score ≥ 10. We completed manual chart reviews to validate the registry. Based on findings from the chart review process, dichotomous branch points were added in succession to the developing workflow.

**Findings:** Table 1 shows the categories of patients in the database. After we completed chart reviews on 150 distinct patients, we found three primary sources of error: 1) EHR generated: PHQ-2 scores auto-populate PHQ-9 scores leading to inaccurate PHQ-9 s; 2) Registry generated: The registry extracted diagnoses only from the Problem list and not from Visit Diagnoses sections of the chart; and 3) Provider diagnosis: Four patients in the registry had concurrent diagnoses of unipolar depression, bipolar depression and personality disorder. Many with depression had no documented PHQ-9.

**Implications for D&I Research**: In developing this registry we uncovered three sources of error. Provider error may have been exacerbated by the EHR. EHR workflows may encourage busy clinicians to prioritize billing documentation rather than clinical accuracy. Our process of creating a registry pointed to the importance of validation of both the electronic medical record and the registry to ensure the efficiency and accuracy of a population health registry.

Primary Funding Source: National Institutes of Health - NIMH K23 1K23MH100162-01A1funded Dr. Loeb's time on this project.

**Table 1 (Abstract S18):** Results of Depression Database of All Patients

Database Categories	
Categories	Number (%)
+PHQ-9 and Depression or Dysthymia	580 (11.8)
+PHQ-9/no Depression or Dysthymia	559 (11.3)
Bipolar or Personality Disorder	131 (2.7)
No PHQ-9*	3369 (68.3)
All PHQ-9’s negative*	296 (6.0)

*Previous 12 months

### S19 Measurement feedback systems as a strategy to support implementation of measurement-based care in behavioral health

#### Aaron Lyon^1^, Cara Lewis^2^, Meredith Boyd^2^, Abigail Melvin^2^, Semret Nicodimos^1^, Freda Liu^1^, Nathanial Jungbluth^3^

##### ^1^Psychiatry and Behavioral Sciences, University of Washington, Seattle, WA, 98115, USA; ^2^Psychological and Brain Sciences, Indiana University, Bloomington, IN, 47405, USA; ^3^Psychology, University of Washington, Seattle, WA, 98115, USA

###### **Correspondence:** Aaron Lyon – Psychiatry and Behavioral Sciences, University of Washington, Seattle, WA, 98115, USA

**Background:** Measurement-based care (MBC) – the systematic, repeated collection of outcome data to evaluate patient progress, provide feedback, and inform intervention decisions – is shown to improve patient outcomes in behavioral health (Bickman et al., 2011; Lambert & Shimkowa, 2011; SAMSHA, 2012). Digital measurement feedback systems (MFS; Bickman, 2008), which collect outcome data and display results to clinicians and patients, represent a rapidly growing implementation strategy with the potential to address workflow issues and streamline MBC integration. A diverse array of MFS have been developed across the academic and commercial sectors yielding a diffuse and siloed knowledge base. System variability and lack of alignment with relevant theories and frameworks may limit the extent to which they can effectively support MBC implementation. This presentation will report findings from a comprehensive review of MFS designed to (a) document their characteristics and capabilities and (b) detail strategies through which they support MBC.

**Methods:** Using a competitive analysis framework (Bergen & Peteraf, 2002), we identified extant MFS and their associated processes for supporting MBC. Data collection involved (1) coding publically available MFS information (websites, scientific literature) and (2) semi-structured interviews with system developers to assess congruence with leading frameworks for feedback (e.g., Feedback Intervention Theory; Kluger & DeNisi, 1996), user centered design (e.g., International Standards Organization, 2010), and implementation science (e.g., Diffusion of Innovations; Rogers, 2003).

**Findings:** MFS capabilities varied widely across 49 identified systems. For example, although most tracked standardized outcomes (94 %), far fewer facilitated the use of more individualized outcomes (29 %). The majority displayed outcomes visually (e.g., graphs) (69 %), but were less likely to provide feedback about outcomes relative to a standard (e.g., assessment tool norms; 42 %) or facilitate data collection via a patient portal (37 %). Although bibliometric data indicated that only 31 systems (63 %) were represented in the literature, 84 % of systems were self-described as “evidence-based.” Additional results from developer interviews will describe implementation supports, costs, and spread.

Implications for D&I Research:

Federal agencies have underscored the importance of technology and outcome evaluation for the modernization and enhancement of healthcare. Identification of MFS components that best support MBC has considerable potential to strengthen client engagement and improve outcomes.

Primary Funding Source: Seattle Children's Research Institute.

### S20 PANEL: Implementation Science and Learning Health Systems: Intersections and Commonalities - Common loop assay: Methods of supporting learning collaboratives

#### Allen Flynn

##### Learning Health Sciences, Medical School, University of Michigan, Ann Arbor, MI, 48109, USA

**Background:** Quality Improvement Collaboratives or Clinical Design Teams are communities with like interests in learning to improve the quality and decrease the cost of health care. Within the context of a Learning Health System, it appears that these types of communities, including their people, processes and technologies, represent a successful vanguard. Their productivity effecting positive change makes them of interest for implementation research. We are developing methods to systematically study collaborative learning communities to describe the work they do to identify problems, disseminate knowledge, and implement change. This research describes the development of an interview-based method to uncover similarities and differences in the policies and practices of these communities. Findings are reported from developing and testing this method.

**Methods:** We iteratively developed a structured interview guide based on a model of ongoing collaborative learning within communities, called a “learning cycle.” The interview guide addresses the formation and prioritization of learning goals within communities and inquires about the means, policies, procedures, roles, technologies in use, and challenges pertaining to the learning cycle model.

**Findings:** Our method of using structured interviews to understand how the policies and practices of communities with like interests in health are similar or different has elicited this type of information in two test cases. Methodologically, we have found it helpful to explicitly relate a known model of structured problem solving, Plan-Do-Check-Act, to the learning cycle model of the interview guide. We have also found that our learning cycle model may need to be adapted to different types of learning goals, e.g., learning to understand the comparative effectiveness of treatment interventions versus learning to understand how to improve the application of best practices.

**Implications for D&I Research:** The interview method under development has the potential to uncover latent contextual factors for success, to elicit the types of expertise needed to facilitate learning cycles, and to describe the current state of technology use within communities with like interests in health. Future work will involve validating this method and using it to understand how to support these communities with an infrastructure made of policy, patient engagement programs, and information technologies.

### S21 PANEL: Implementation Science and Learning Health Systems: Intersections and Commonalities - Innovating audit and feedback using message tailoring models for learning health systems

#### Zach Landis-Lewis

##### Learning Health Sciences, University of Michigan Medical School, Ann Arbor, MI, 48109, USA

**Background:** Feedback loops are a key feature of a learning health system, a cybersocial infrastructure that enables continuous learning from data to improve the delivery of patient-centered care. Audit and feedback interventions are likely to be effective when they address healthcare providers’ barriers to behavior change, but these barriers commonly differ across individual healthcare providers, reducing their effectiveness. The purpose of this research is to innovate feedback interventions by developing message tailoring systems (MTSs) that increase the likelihood that feedback influences an individual’s barriers to behavior change.

**Methods:** We developed models for feedback message tailoring focused on antimicrobial stewardship. We developed a prototype feedback MTS to generate menus of graphical and textual messages for selection by clinical supervisors. Supervisors could use menus to tailor messages based on their awareness of feedback recipients' specific barriers to behavior change. We adapted our prototype MTS to the domain of HIV/AIDS treatment by mapping relationships between causal mechanisms of performance feedback, behavior change barriers in HIV/AIDS treatment, components of feedback messages, and features of clinical performance (e.g. high or low performance). We evaluated the adapted model by analyzing clinical performance data from HIV/AIDS clinics in Malawi where an electronic medical record system is used. We identified performance gaps between healthcare providers for peer benchmarking and described variability in tailored feedback messages for four guideline based performance indicators addressing care documentation, prescribing, and WHO clinical staging.

**Findings:** We retrospectively analyzed 7,448 monthly performance reports from 11 HIV/AIDS clinics. The average number of performance gaps for all 11 sites ranged from 0.32 to 2.45 gaps per month. We found that tailored feedback messages could be routinely generated for all four performance indicators, with 35 % of reports having messages prioritized to optimize feedback effectiveness.

**Implications for D&I Research:** A foundation of health related evidence and knowledge supports the development of clinical feedback MTSs. Creating and evaluating MTSs is a promising approach to improving clinical feedback interventions. This research establishes proof of concept for an innovative approach to improving clinical performance feedback in low-resource settings and suggests possible directions for prospective evaluations comparing alternative feedback message designs.

Primary Funding Source: National Institutes of Health.

### S22 PANEL: Implementation Science and Learning Health Systems: Intersections and Commonalities - Implementation science and learning health systems: Connecting the dots

#### Anne Sales^1,2^

##### ^1^Learning Health Sciences, University of Michigan, Ann Arbor, MI, 48109, USA; ^2^Center for Clinical Management Research, VA Ann Arbor Health Care System, Ann Arbor, MI, 48109, USA

**Background:** Implementation science (IS) has been growing over the last two decades. Over the last decade, increasing interest has developed in applying organizational theory, systems science, data science, information technology, and IS, to the study and organization of learning health systems (LHS). This is critical in an era of rapidly evolving large electronic health data systems. In this presentation, we emphasize linkages between the emerging sciences of LHS and IS, noting where advances in IS can contribute significantly to LHS.

**Methods:** Literature review and concept analysis to describe linkages in the emerging literature in two distinct fields: LHS and IS. We use the learning health cycle as a heuristic device to examine commonalities and differences, and focus on three widely used IS frameworks.

**Findings:** The literature theorizing LHS is growing rapidly. While it draws on long-standing theories of learning organizations, it strongly emphasizes the importance of data, especially electronic health record data, to generate knowledge. The learning cycle of LHS has strong similarities in knowledge generation with the Knowledge to Action (KTA) cycle. The emphasis on knowledge generation through empirical data analysis is quite distinct from the emphasis in KTA on evidence generated through multiple empirical studies using meta-analysis. While the learning cycle includes behavioral change components, there is a primary focus on advice giving through feedback reports and decision support. Individual behavioral change techniques, developing in conjunction with the Theoretical Domains Framework (TDF), and organizational change strategies, associated with the Consolidated Framework for Implementation Research (CFIR), receive less attention. Additionally, there are other data sources that may be useful for both knowledge generation and behavior change, such as social media, which are not part of electronic health records but are receiving attention in IS.

**Implications for D&I Research:** IS, through its rapidly evolving theory-driven strategies and approaches, can provide important, measurable inputs into learning cycles for LHS. It can provide a wider range of behavioral techniques and organizational strategies to promote practice change, based on multiple sources of knowledge, and approaches using social media and other information technologies can inform both data for knowledge generation and provide new change strategies.

## Clinical care settings

### S23 Facilitation activities of Critical Access Hospitals during TeamSTEPPS implementation

#### Jure Baloh, Marcia Ward, Xi Zhu

##### Department of Health Management and Policy, University of Iowa, Iowa City, IA, 52242, USA

###### **Correspondence:** Jure Baloh – Department of Health Management and Policy, University of Iowa, Iowa City, IA, 52242, USA

**Background:** The PARIHS (Promoting Action on Research Implementation in Health Services) model proposes facilitation as one of the key elements for successful implementation of evidence-based practices (EBP). Little is known about the facilitation activities hospitals engage in when implementing EBP. We set out to identify types and temporal patterns of facilitation activities of Critical Access Hospitals (CAHs) implementing TeamSTEPPS (Team Strategies and Tools to Enhance Performance and Patient Safety).

**Methods:** Key informants from 10 CAHs that initiated TeamSTEPPS implementation in 2011 and 2012 were interviewed quarterly for a period of two years and provided information on implementation activities and progress. Based on the data from the first two quarters, four coders inductively developed a coding template of major types of facilitation activities. The template was then deductively applied to the remaining transcripts, with pairs of coders coding each transcript. All four coders reviewed the coded content and reconciled the differences. Finally, temporal patterns of facilitation activities were examined as a frequency of quotes in each quarter for each facilitation type.

**Findings:** Four major types of facilitation activities were identified – leadership (e.g. role modeling and publicly rewarding strong performance), buy-in (e.g. obtaining buy in from clinicians and administrators, dealing with resistance), customization (e.g. tailoring TeamSTEPPS to local context, use of tools), and accountability (e.g. audits and evaluation, reinforcement). All hospitals engaged in all types of facilitation activities at least to a certain extent. Preliminary analyses of temporal patterns show that leadership and buy-in were more prominent during the first six months of the implementation, and then leveled off as the implementation progressed. Customization and accountability had a less pronounced temporal pattern; however, while customization also peaked early in the implementation process, accountability became more prominent after the first nine months.

**Implications for D&I Research:** In our study, we expanded the empirical literature on activities that facilitate implementation of EBP in hospitals. We identified four major types of facilitation activities, and found some evidence supporting the notion that facilitation activities change over time. Additional research is needed to establish the antecedents and consequences of these activities, and to further develop our understanding of how facilitation develops over time.

Primary Funding Source: Agency for Healthcare Research and Quality - Grant number R18HS018396.

### S24 Organizational and social context of federally qualified health centers and variation in maternal depression outcomes

#### Ian Bennett^1^, Jurgen Unutzer^2^, Johnny Mao^3^, Enola Proctor^4^, Mindy Vredevoogd^2^, Ya-Fen Chan^2^, Nathaniel Williams^5^, Phillip Green^6^

##### ^1^Family Medicine, University of Washington, Seattle, WA, 98105-6099, USA; ^2^Psychiatry & Behavioral Sciences, University of Washington, Seattle, WA, 98195, USA; ^3^Center for Clinical and Epidemiological Research, University of Washington, Seattle, WA, 98101, USA; ^4^George Warren Brown School of Social Work, Washington University in St. Louis, Saint Louis, MO, 63130, USA; ^5^Social Work, Boise State University, Boise, ID, 83725, USA; ^6^School of Social Work, University of Tennessee, Knoxville, TN, 37996, USA

###### **Correspondence:** Ian Bennett – Family Medicine, University of Washington, Seattle, WA, 98105-6099, USA

**Background:** Organizational factors have been found to influence the success of implementation efforts for clinical innovations however this has not been assessed in primary care settings implementing collaborative care for depression. We wished to assess the influence of organizational factors on effectiveness of the collaborative care model of integrated depression services in primary care sites serving low income women.

**Methods:** The organizational social context (OSC) of twelve federally qualified health centers participating in a program to implement and sustain collaborative care for depression among women in pregnancy and parenting was assessed. OSC surveys were carried out with members of the collaborative care teams from each site. Women who initiated care in the year before this assessment to nine months after were included in the analysis. Three level hierarchical linear modeling was carried out to assess the association of OSC domains with rates of improvement in PhQ-9 scores over time as determined by the average linear rate of change per month.

**Findings:** Significant variation was seen in rates of depression improvement across health centers (P < 0.001). In HLM modeling proficiency and engagement were significantly associated with greater average linear rates of change (ratio of change for high versus low OSC domains was 1.62 and 1.80 respectively) of depression symptoms while stress was associated with slower average linear rates of change (ratio of change for high versus low OSC domains was 0.51).

Implications for D&I Research: The effectiveness of collaborative care was associated with ratings of organizational factors related to proficiency, engagement, and stress in federally qualified health centers caring for low income women. These measures may direct interventions to improve the effectiveness of care for depression in these settings.

Primary Funding Source: Private foundations.

### S25 Decision support to enhance treatment of hospitalized smokers: A randomized trial

#### Steven Bernstein^1,2^, June-Marie Rosner^1^, Michelle DeWitt^3^, Jeanette Tetrault^4^, James Dziura^1^, Allen Hsiao^5^, Scott Sussman^6^, Patrick O'Connor^7^, Benjamin Toll^8^

##### ^1^Emergency Medicine, Yale University School of Medicine, New Haven, CT, 06519, USA; ^2^Cancer Prevention and Control, Yale Cancer Center, New Haven, CT, 06519, USA; ^3^Information Technology Services, Yale-New Haven Hospital, New Haven, CT, 06510, USA; ^4^Medicine, Yale School of Medicine, New Haven, CT, 06519, USA; ^5^Pediatrics and Emergency Medicine, Yale-New Haven Hospital, New Haven, CT, 06510, USA; ^6^Medicine, Yale-New Haven Hospital, New Haven, CT, 06510, USA; ^7^Internal Medicine, Yale University School of Medicine, New Haven, CT, 06520, USA; ^8^Public Health Sciences, Medical University of South Carolina, Charleston, SC, 29425, USA

###### **Correspondence:** Steven Bernstein – Emergency Medicine, Yale University School of Medicine, New Haven, CT, 06519, USA

**Background:** Tobacco dependence treatment for hospitalized smokers results in long-term cessation if treatment continues at least 30 days post-discharge. **Methods** to leverage inpatient interventions into post-hospitalization care are unclear, and health information technology may facilitate ongoing treatment. The objective of this study is to develop and test an order set and best practice alert (BPA) addressing tobacco dependence treatment for hospitalized smokers embedded in an electronic health record (EHR).

**Methods:** A 2-arm randomized clinical trial of 254 physicians and patients treated by those physicians. The physician is the unit of randomization. A BPA and order set were developed and embedded in the Epic (Madison, WI) EHR used at 2 hospitals in a single city. When an adult patient is admitted to a medical service, a BPA fires if the patient is coded in the EHR as a smoker. For physicians randomized to the intervention, the BPA offers to take the physician to an order set to prescribe tobacco treatment medications and refer the patient to the state smokers’ quitline. Additionally, “tobacco use disorder” is added to the patient’s problem list, and an email is sent to the patient’s primary care provider (PCP). In the control arm, a BPA silently fires with no additional actions offered for the physician.

**Findings:** From August 2013 to July 2015, the BPA fired for 8519 patients (4164 intervention, 4355 control). Compared to control arm physicians, intervention physicians were more likely to order tobacco treatment medication (35 % v. 29 %, P < 0.0001, chi-square test), populate the problem list with tobacco use disorder (41 % v. 2 %, P < 0.0001), and make a referral to the state smokers’ quitline (30 % v. 0 %, P < 0.0001). In addition, intervention physicians sent an email to the patient’s primary care provider 4152 (99 %) times.

**Implications for D&I Research**: Designing and implementing an order set and BPA for tobacco treatment in an EHR is feasible and acceptable, and results in an increase in physician orders for tobacco treatment medication, referrals to the state smokers’ quitline, and email to patients’ PCP. This approach is widely replicable, and may be implemented across inpatient, outpatient, and emergency care settings.

Primary Funding Source: National Institutes of Health - R18HL105208.

### S26 PANEL: Developing Sustainable Strategies for the Implementation of Patient-Centered Care across Diverse US Healthcare Systems - A patient-centered approach to successful community transition after catastrophic injury

#### Michael Jones^1^, Julie Gassaway^2^

##### ^1^Research, Shepherd Center, Atlanta, GA, 30312, USA; ^2^ Virginia C. Crawford Research Institute, Atlanta, GA 30309, USA

###### **Correspondence:** Michael Jones – Research, Shepherd Center, Atlanta, GA, 30312, USA

**Background:** Serial innovative, patient-centered approaches to support successful transition to the community after inpatient rehabilitation for patients with severe, traumatic spinal cord injuries (SCI) were implemented with funding from PCORIs healthcare systems initiative. The study team identified three interventions for development and evaluation: 1) peer-directed patient and family care management training to take advantage of new, interactive learning approaches; 2) one-to-one mentoring to provide guidance and support from peers with similar injuries; and 3) a new patient engagement portal for patients and families to communicate with each other, peer mentors, and clinical staff, and to access information related to their care needs. All approaches tested intended to promote self-efficacy, or greater self-confidence that patients and their family can effectively manage their care needs.

**Methods:** A randomized, controlled trial was completed with 158 participants to evaluate the effectiveness of one-to-one peer mentoring during the inpatient stay, on patient self-efficacy and, ultimately, clinical outcomes. A stepped wedge design was used to evaluate the effects of the peer-directed patient education, compared to our traditional classroom training. The revised education program was implemented step-wise across three classes: bladder management, skin care, and general health concerns.

**Findings:** Significant differences were noted between intervention and control group participants in improved reported self-efficacy. Significant improvements were noted in participant engagement across all three classes with implementation of the revised training approach. The stepped wedge design advanced the implementation strategy of “leaving the intervention going” after demonstrated improvements in patient engagement.

**Implications for D&I Research:** The challenges associated with patient-centered healthcare system research will be discussed particularly with respect to experimental control (e.g., the messiness of real world implementation and need for practical research designs such as the stepped wedge design); the long-term nature of intervention studies addressing prevention efforts (i.e., time required to collect long-term outcome measures to validate the relationship between improved self-efficacy and healthcare outcomes); and sustainability (e.g., the advantage of system changes that require an initial investment and support for implementation but, once implemented, can be readily sustained).

Primary Funding Source: Patient-Centered Outcomes Research Institute.

### S27 PANEL: Developing Sustainable Strategies for the Implementation of Patient-Centered Care across Diverse US Healthcare Systems - Conducting PCOR to integrate mental health and cancer screening services in primary care

#### Jonathan Tobin

##### Community Engaged Research, Clinical Directors Network, Inc. & The Rockefeller University, New York, NY, 10018, USA

**Background:** We describe the results from three NCI-funded studies that informed a current patient-centered outcomes research (PCOR) study comparing two evidence-based care management (CM) strategies, “Cancer Prevention Care Management (PCM) and PCM plus “Depression Collaborative Care Intervention” (PCM + CCI) to improve mental health and cancer screening outcomes for low-income minority women who receive primary care from Bronx, NY Community Health Centers (CHCs).

**Methods:** An ongoing PCORI Health Systems-funded individual level randomized clinical trial (RCT) has enrolled six CHCs and several community-based organizations (CBOs) that serve the same communities and which provide social services (e.g., assistance with housing, immigration, legal, entitlements, English language, job skills). We implemented an enhanced reciprocal referral process among the CHCs and CBOs to better meet the multiple unmet social and clinical needs of the low income minority women they each serve.

**Findings:** An extensive stakeholder engagement process to implement the two interventions created an active and ongoing collaboration among CHC clinical leadership, front line clinicians, care managers, office staff, CBO leadership and patients with direct personal experience (self and/or family) with cancer.

**Implications for D&I Research:** Study implementation requires careful attention to stakeholder engagement, clinical workflow, organizational hiring practices and patient preferences. This presentation considered the importance of these factors as well as their effects on local adaptation, treatment fidelity, effectiveness and sustainability of two evidence-based interventions (PCM vs PCM + CCI) tested in combination. Specifically, we examined the trade-offs between providing CMs funded by the research project that are external (research-funded, higher internal treatment fidelity and validity) vs internal (CHC-funded, higher adaptation, external validity and sustainability), and the impact on local adaptation of the intervention (treatment fidelity), effectiveness (self-efficacy of staff and patients, improvements in rates of depression control and cancer screening tests) and sustainability (post-study funding utilization).

Primary Funding Source: Patient-Centered Outcomes Research Institute - IH-12-11-4522.

### S28 PANEL: Developing Sustainable Strategies for the Implementation of Patient-Centered Care across Diverse US Healthcare Systems - A comparative effectiveness trial of optimal patient-centered care for US trauma care systems

#### Douglas Zatzick

##### Department of Psychiatry and Behavioral Sciences, University of Washington, Seattle, WA, 98104, USA

**Background:** The overarching goal of this PCORI sponsored project is to integrate optimal models of patient-centered care into acute care medical to primary care and community care transitions for US trauma care systems. Our multi-stakeholder team that includes front-line providers, patients, researchers and policy makers has been working for over a decade to integrate optimal care transition models into trauma care.

**Methods:** The team’s staged implementation efforts began with clinical epidemiologic investigations and psychometric studies of the patient-centered construct posttraumatic concerns. Later investigations included clinical trials that tested patient-centered engagement strategies. The team is currently conducting a PCORI supported comparative effectiveness trial that is testing optimal models of patient-centered care transitions and uses patient concerns as the primary outcome measure. In the final year of the contract, the study team will convene an American College of Surgeons policy summit that targets the widespread implementation of patient-centered care practices derived from trial results through policy guidelines.

**Findings:** In initial clinical epidemiologic studies, 100 % of randomly sampled injured trauma survivors demonstrated one or more posttraumatic concerns over the course of the year after injury. Patients’ post-injury concerns spanned physical health, work and finance, friends and family, medical care and other domains. Longitudinal studies of randomly sampled injured trauma survivors (N = 101) which demonstrated that the severity and trajectory of posttraumatic concerns mirrored the trajectories of posttraumatic psychological symptoms and physical functioning; these findings emphasized the potential importance of patient-centered assessments and interventions to acute care medical policy makers who have the capacity to mandate sustainable changes in care. Posttraumatic concern assessment and amelioration was successfully used in clinical trial designs to engage over 90 % of injured patients randomized to stepped care interventions; injured patients were successfully engaged in care that linked the trauma center admission with outpatient services.

**Implications for D&I Research:** Staged research projects that begin with initial clinical epidemiological investigations and lead to later randomized comparative effectiveness trials can further the implementation of patient-centered care models in US healthcare systems. Orchestrated investigative and policy efforts can work synergistically to develop and implement optimal models of patient-centered care transitions across US trauma health care systems.

Primary Funding Source: Patient-Centered Outcomes Research Institute - IH-1304-6319.

### S29 Preferences for in-person communication among patients in a multi-center randomized study of in-person versus telephone communication of genetic test results for cancer susceptibility

#### Angela R Bradbury^1,2^, Linda Patrick-Miller^3^, Brian Egleston^4^, Olufunmilayo I Olopade^5^, Michael J Hall^6^, Mary B Daly^7^, Linda Fleisher^8^, Generosa Grana^9^, Pamela Ganschow^10^, Dominique Fetzer^11^, Amanda Brandt^11^, Dana Farengo-Clark^12^, Andrea Forman^7^, Rikki S Gaber^13^, Cassandra Gulden^5^, Janice Horte^12^, Jessica Long^11^, Rachelle Lorenz Chambers^5^, Terra Lucas^14^, Shreshtha Madaan^15^, Kristin Mattie^12^, Danielle McKenna^11^, Susan Montgomery^7^, Sarah Nielsen^5^, Jacquelyn Powers^11^, Kim Rainey^7^, Christina Rybak^7^, Michelle Savage^7^, Christina Seelaus^13^, Jessica Stoll^5^, Jill Stopfer^11^, Shirley Yao^13^, Susan Domchek^1^

##### ^1^Division of Hematology-Oncology, Department of Medicine, The University of Pennsylvania, Philadelphia, PA, 19104, USA; ^2^Department of Medical Ethics and Health Policy, The University of Pennsylvania, Philadelphia, PA, 19104, USA; ^3^Center for Clinical Cancer Genetics Department of Medicine, Hematology/Oncology, The University of Chicago, Chicago, IL, 60637, USA; ^4^Biostatistics and Bioinformatics Facility, The Fox Chase Cancer Center, Philadelphia, PA, 19111, USA; ^5^Center for Clinical Cancer Genetics, The University of Chicago, Chicago, IL, 60637, USA; ^6^Clinical Genetics/Gastrointestinal Risk Assessment, The Fox Chase Cancer Center, Philadelphia, PA, 19111, USA; ^7^Department of Clinical Genetics, The Fox Chase Cancer Center, Philadelphia, PA, 19111, USA; ^8^Center for Injury Research and Prevention, The Children's Hospital of Philadelphia, Philadelphia, PA, 19104, USA; ^9^Hematology and Medical Oncology, MD Anderson Cancer Center at Cooper, Camden, NJ, 08043, USA; ^10^Internal Medicine, John H. Stroger, Jr. Hospital, Chicago, IL, 60612, USA; ^11^Mariann and Robert MacDonald Women's Cancer Risk Evaluation Program, The University of Pennsylvania, Philadelphia, PA, 19104, USA; ^12^Cancer Genetics Program, MD Anderson Cancer Center at Cooper, Camden, NJ, 08043, USA; ^13^Breast and Cervical Cancer Screening Program, John H. Stroger, Jr. Hospital, Chicago, IL, 60612, USA; ^14^Cancer Screening Program, John H. Stroger, Jr. Hospital, Chicago, IL, 60612, USA; ^15^ Comprehensive Cancer Risk and Prevention Clinic, The University of Chicago, Chicago, IL, 60637, USA

###### **Correspondence:** Angela R Bradbury – Division of Hematology-Oncology, Department of Medicine, The University of Pennsylvania, Philadelphia, PA, 19104, USA

**Background:** Genetic testing for cancer predisposition has become standard-of-care and traditionally includes in-person (IP) pre- and post-test genetic counseling. In 2013, multigene panel testing became available, although the clinical utility remains unclear. Recently, telephone delivery of genetic services has been found to be equivalent to IP delivery in *BRCA1/2*testing, but has not been evaluated in multigene testing where there is greater potential for uncertainty, distress and misunderstanding. Additionally, some patients express preferences for IP communication and may be vulnerable to inferior outcomes with telephone delivery.

**Methods:** 864 participants (64 % of approached) have enrolled in a multi-center randomized trial of telephone (TD) versus in-person disclosure (IPD) of genetic test results for breast, colon and gynecologic cancer susceptibility. In 2013, the study was adapted to include multigene testing. Participants not willing to receive results by phone enrolled in a third arm (self-select IPD). We used a multiple logistic regression to evaluate factors associated with declining randomization and differences in cognitive and affective outcomes between participants agreeing to randomization (n = 719) and those selecting for IPD (n = 145).

**Findings:** In months 1-16, when only *BRCA1/2* testing was offered, 18 % self-selected IPD. With the advent of multigene testing (months 17-33), 9 % of those undergoing targeted testing (e.g. *BRCA1/2* testing only) self-selected IPD v. 21 % undergoing multigene testing (p = 0.001). Selecting IPD was associated with higher baseline general anxiety (p = 0.01) and depression (p = 0.07), older age (p = 0.06) and having multigene testing (p = 0.004). In multivariable analyses, only multigene testing (OR 4.8; p < 0.001), *BRCA1/2*testing in months 1-16 (OR 2.9, p = 0.006) and being older (OR 1.04/year, p = 0.001) were statistically significant. Compared to participants in the randomized arms, there were no differences in change in knowledge, anxiety, depression, cancer worry or satisfaction after receipt of results.

**Implications for D&I Research:** Although telephone communication may provide similar patient reported outcomes for *BRCA1/2* testing, with multigene testing the outcomes remain unknown. More patients of older age, with baseline anxiety and undergoing multigene testing express a preference for in-person communication. Until further data are available, the option for in-person communication (when available) should be provided to patients seeking genetic testing for cancer susceptibility, particularly those undergoing multigene testing.

Primary Funding Source: National Institutes of Health - NIH R01 CA160847.

### S30 Working towards de-implementation: A mixed methods study in breast cancer surveillance care

#### Erin Hahn, Corrine Munoz-Plaza, Jianjin Wang, Jazmine Garcia Delgadillo, Brian Mittman, Michael Gould

##### Research and Evaluation, Kaiser Permanente Southern California, Pasadena, CA, 91101, USA

###### **Correspondence:** Erin Hahn – Research and Evaluation, Kaiser Permanente Southern California, Pasadena, CA, 91101, USA

**Background:** De-implementing commonly used but ineffective clinical practices is an important component of quality. Oncology offers several opportunities to reduce use of ineffective practices based on guidelines from the American Society of Clinical Oncology (ASCO). We studied the use of one such practice, biomarker blood tests for breast cancer surveillance, within an integrated health care system. We documented utilization patterns and explored provider perceptions and attitudes to inform de-implementation efforts.

**Methods:** Using a sequential explanatory mixed methods design, we identified a cohort of early stage breast cancer survivors and calculated the number and frequency of biomarker tests during an 18-month post-treatment period. We identified high and low utilizing medical centers and conducted semi-structured qualitative interviews with oncologists in both types of centers, guided by the Theoretical Domains Framework. Interviews were transcribed, coded, and analyzed.

**Findings:** Among 7,363 patients diagnosed between 2009-2012, 40,114 biomarker tests were ordered for 41 % of patients. We found significant variation by medical center: 5 %-78 % of eligible patients received a test. We interviewed 18 oncologists in high and low utilizing centers. Several themes emerged, including: 1) Awareness of non-adherence: oncologists reported awareness of current ASCO guidelines and agreed that biomarkers are not clinically useful; high-utilizers acknowledge intentional non-adherence (“*We all know we shouldn’t do this but do it anyways*”); 2) Anxiety: despite agreeing that biomarkers aren’t useful, some oncologists are anxious about missing a recurrence and want to do “all possible” to prevent this; high-utilizers perceive that patients are highly anxious and desire a quantitative test for reassurance (“*They need a number*”); 3) Perceived patient expectations: oncologists perceive competition from other systems and are concerned about perception of withholding care (“*If [competitor] does it…patients expect it*”) and implications for patient satisfaction.

**Implications for D&I Research:** Barriers to de-implementation are numerous and complex. Traditional strategies of practice change based on increasing awareness and knowledge (provider education, electronic alerts) are unlikely to be effective. Multi-faceted, multi-level strategies deployed to address consumer-, clinician-, and system-related barriers are required. Research-based development and evaluation of multi-level de-implementation strategies is critical and will help develop valuable insights and theories regarding the determinants of clinical practices and opportunities to influence them.

### S31 Integrating evidence-based practices for increasing cancer screenings in safety-net primary care systems: A multiple case study using the consolidated framework for implementation research

#### Shuting (Lily) Liang^1,2^, Michelle C. Kegler^1,3^, Megan Cotter^4^, Emily Phillips^4^, April Hermstad^4^, Rentonia Morton^5^, Derrick Beasley^4^, Jeremy Martinez^5^, Kara Riehman^5^

##### ^1^Emory Prevention Research Center, Emory University Rollins School of Public Health, Atlanta, GA, 30322, USA; ^2^Research Institute, Palo Alto Medical Foundation, Palo Alto, CA, 94301, USA; ^3^Department of Behavioral Sciences and Health Education, Emory University Rollins School of Public Health, Atlanta, GA, 30322, USA; ^4^Behavioral Sciences and Health Education, Rollins School of Public Health, Emory Prevention Research Center, Atlanta, GA, 30322, USA; ^5^Statistics and Evaluation Center, American Cancer Society, Atlanta, GA, 30303, USA

###### **Correspondence:** Shuting (Lily) Liang – Emory Prevention Research Center, Emory University Rollins School of Public Health, Atlanta, GA, 30322, USA

**Background:** Implementing evidence-based practices (EBPs) to increase cancer screenings in safety-net primary care systems has great potential for reducing cancer disparities. Yet there is a gap in understanding the factors and mechanisms that influence EBP implementation across such highly-variable systems. Guided by the Consolidated Framework for Implementation Research (CFIR), our study aims to fill this gap with a multiple case study approach to examine how CFIR constructs influence EBP implementation in safety-nets that were funded by an American Cancer Society initiative to increase breast and colorectal cancer screenings.

**Methods:** Nine systems participating in the initiative were categorized into high-, medium- and low- performing sites based on process and outcome evaluation data. Fifty-two interviews were conducted with project leaders and implementers. Two researchers independently coded each transcript using CFIR constructs and then met to resolve discrepancies. Within- and cross-site analyses with construct rating were performed to identify how CFIR constructs were related to EBP implementation.

**Preliminary findings:** Of 39 constructs examined, 13 constructs demonstrated salience in influencing implementation with frequent references by a majority of interviewees across all 9 systems. Five were from the inner setting: structural characteristics, network and communication, compatibility, leadership engagement and available resources. Four were from the process domain: planning, formally appointed internal implementation leaders, executing, and reflecting and evaluating; two were from outer setting (patient needs and resources and cosmopolitanism) and two were from individual characteristics domain (knowledge and beliefs about the intervention and personal attributes). However, only leadership engagement and formally appointed internal implementation leaders distinguished high-, medium- and low- performing sites. Other distinguishing but less salient constructs included adaptability, design quality and packaging, tension for change, and access to information and knowledge. Detailed descriptions of how each construct manifested across systems and recommendations for practices in safety-nets will be provided.

Implications for D&I Research: Our study identified a number of influential CFIR constructs and illustrated how they impact EBP implementation across a variety of safety-net systems. Findings may inform future dissemination efforts of EBPs for increasing cancer screenings in similar settings. Moreover, our analytic approach is similar to previous case studies using CFIR and hence could facilitate comparisons across studies.

Primary Funding Source: American Cancer Society.

### S32 Observations from implementing an mHealth intervention in an FQHC

#### David Gustafson^1^, Lisa Marsch^2^, Louise Mares^3^, Andrew Quanbeck^4^, Fiona McTavish^1^, Helene McDowell^1^, Randall Brown^5^, Chantelle Thomas^6^, Joseph Glass^7^, Joseph Isham^1^, Dhavan Shah^8^

##### ^1^Center for Health Enhancement Systems Studies, University of Wisconsin – Madison, Madison, WI, 53706, USA; ^2^Center for Technology and Behavioral Health, Dartmouth College, Dartmouth, NH, 03755, USA; ^3^Communication Arts, University of Wisconsin – Madison, Madison, WI, 53706, USA; ^4^Industrial and Systems Engineering, University of Wisconsin – Madison, Madison, WI, 53706, USA; ^5^Family Medicine, Population Health Sciences, University of Wisconsin School of Medicine & Public Health, Madison, WI, 53715, USA; ^6^Access Community Health Center, Access Community Health Center, Madison, WI, 53706, USA; ^7^School of Social Work, University of Wisconsin – Madison, Madison, WI, 54706, USA; ^8^Communication Sciences, University of Wisconsin – Madison, Madison, WI, 53706, USA

###### **Correspondence:** David Gustafson – Center for Health Enhancement Systems Studies, University of Wisconsin – Madison, Madison, WI, 53706, USA

**Background:** FQHCs are being encouraged to integrated addiction and other behavioral health into their services. Traditional approaches create cultural, workflow and staffing issues for primary care and behavioral health providers.

**Methods:** Wisconsin and Dartmouth developed, refined and built an evidence base for two technological systems (CHESS and TES). TES focuses on treatment; CHESS on relapse prevention. Combined, they create a seamless system benefiting from the strengths of each. We added a Clinician Report that helps clinicians use Seva data for patient and population management. We will: introduce Seva, describe the process we used to implement it in suburban, rural and urban FQHCs, the insights coming from implementation and its implications for theory and application of implementation science in this context. Three conceptual models were employed: Self Determination theory (for Seva development), the Organizational Change Model (to predict and explain implementation results) and RE-AIM to assess implementation success.

**Findings:** Our stepped wedge design limits our ability to generalize. However, our observations suggest that:

- While such models are useful at macro level, there is a substantial gap between what the models offer and what is needed for successful application.
- A significant contrast exists between how clinicians want to use Seva and what expectations patients should have for that use.
- There were surprises in who would use the system and what it takes to ensure continued use.
- We were surprised by staff, management and patient openness to Seva, but also by how important ongoing marketing is to continued success.

**Implications for D&I Research:** The value of theories and models became apparent in development, implementation and evaluation. We also noticed a gap between what those theories offer and what is needed to successfully implement an innovation. Those gaps (most apparent in efforts to apply rather than just understand) call for new layers of implementation research; e.g. there is general agreement that senior leader support improves chances of implementation support. The question is HOW to get that support, especially when there is wide variation in leader temperament, values, and style across organizations. Similar issues apply to all implementation model or conceptual framework. This presentation will illustrate those issues and suggest future research.

Primary Funding Source: National Institutes of Health - This study was funded by NIDA R01DA034279

### S33 A multicomponent intervention to improve primary care provider adherence to chronic opioid therapy guidelines and reduce opioid misuse: A cluster randomized controlled trial protocol

#### Jane Liebschutz^1^, Karen Lasser^1,2^

##### ^1^Clinical Addiction Research and Education (CARE) Unit, Boston Medical Center/Boston University School of Medicine, Boston, MA, 02118, USA; ^2^Boston University School of Public Health, Boston, MA, 02118, USA

###### **Correspondence:** Karen Lasser – Clinical Addiction Research and Education (CARE) Unit, Boston Medical Center/Boston University School of Medicine, Boston, MA, 02118, USA

**Background:** Despite the public health significance of prescription opioid misuse, few Primary Care Providers (PCPs) follow standard practice guidelines regarding assessment and monitoring. Based on the Chronic Care Model, we conducted a cluster randomized controlled trial to determine whether a package of implementation strategies will increase PCP adherence to chronic opioid therapy guidelines and reduce opioid misuse among patients, relative to electronic tools alone.

**Methods:** We included 53 PCPs and an estimated 1200 patients from three community health centers and one urban safety-net hospital-based primary care practice who have at least four patients on long-term opioid treatment for chronic non-cancer pain. We enrolled participants (PCPs) from December 2012 through March 2015. PCPs were randomized to receive the intervention for 12 months, which includes four components: 1) nurse care management, 2) use of a patient registry, 3) academic detailing, and 4) electronic tools, or a control condition, which includes only the use of the electronic tools. The intervention PCPs received the services of a nurse-managed registry for planning individual patient care and conducting population-based care for patients receiving chronic opioid therapy. In academic detailing visits, trained co-investigators provided intervention PCPs with individualized education to change prescribing practice. Electronic tools, available to PCPs in both study arm at www.mytopcare.org, include validated instruments to assess patient status, and management resources to facilitate PCP adherence to suggested monitoring. The primary outcomes are PCP adherence to chronic opioid therapy guidelines and patient opioid misuse. Secondary outcomes include measures of substance abuse, possible opioid diversion, and level of opioid risk among patients.

**Findings:** Thus far, the intervention has been successfully implemented in all sites. The main barriers to implementation included personnel changes, and electronic health record transitions. Two sites already decided to sustain the model before the completion of the study, with overwhelmingly positive receptivity to the intervention at all sites.

**Implications for D&I Research:** Delivering an intervention package based on the Chronic Care model, designed to decrease the workload and improve adherence to standard of practice has been well received in safety-net primary care settings. Analysis at study completion will measure whether it improved PCP adherence to guidelines and reduced opioid misuse among patients.

Primary Funding Source: National Institutes of Health - This study is funded by NIDA R01DA034252.

### S34 Implementing collaborative care for substance use disorders in primary care: Preliminary findings from the summit study

#### Katherine Watkins^1^, Allison Ober^1^, Sarah Hunter^1^, Karen Lamp^2^, Brett Ewing^1^

##### ^1^RAND Corporation, RAND Corporation, Santa Monica, CA, 90407, USA; ^2^Venice Family Clinic, Venice Family Clinic, Venice, CA, 90291, USA

###### **Correspondence:** Katherine Watkins – RAND Corporation, RAND Corporation, Santa Monica, CA, 90407, USA

**Background:** Despite the existence of effective treatments, few primary care providers identify and treat substance use disorders (SUDs). Research on introducing new practices suggests that intervention at both the organizational level (i.e., to increase organizational readiness to adopt new practices) and service delivery system level (i.e., reorganizing how care is provided to support the new practice) may be necessary to integrate and sustain the introduction of new treatments.

**Methods:** To address the hypothesized need for change at two levels, we designed and conducted a multi-level study at a large FQHC in Los Angeles County. To create organizational readiness, we employed tools and activities known to facilitate adoption of evidence based practices. At the service delivery system level, we used Wagner’s Chronic Care Model to reorganize and guide care. Organizational readiness was assessed by conducting focus groups, surveys, and one-on-one interviews. Treatments supported included medication-assisted therapy with either injectable naltrexone or buprenorphine-naloxone, and a 6-session brief psychotherapy. We report the effects of the organizational readiness intervention on provider measures of treatment acceptability, appropriateness, feasibility and intention to adopt—at five time points using a pre-post design and repeated measures analysis of variance as well as qualitative analysis.

**Findings:** Preliminary results indicate that perceptions of medication acceptability and effectiveness changed significantly from Year 1 to Year 2. With regard to appropriateness, staff agreed more after the intervention that SUDs could be treated in primary care settings, that they could be effectively treated at their clinic, and that providing medications fits the mission of the clinic. In the feasibility domain, medical providers felt more prepared to identify and treat patients with SUDs.

**Implications for D&I Research:** Organizational readiness refers to “the extent to which organizational members are psychologically and behaviourally prepared to implement organizational change.” We found that an organizational readiness intervention favorably increased primary care provider and non-provider perceptions about providing SUD treatment. Future work will examine whether the dual intervention leads to improved service delivery and patient outcomes.

Primary Funding Source: National Institutes of Health - Funded by NIDA R01DA034266, PI: Katherine Watkins.

## Global dissemination and implementation research

### S35 Sustaining a task-shifting strategy for blood pressure control in Ghana: A stakeholder analysis

#### Juliet Iwelunmor^1^, Joyce Gyamfi^2^, Sarah Blackstone^1^, Nana Kofi Quakyi^3^, Jacob Plange-Rhule^4^, Gbenga Ogedegbe^2,5^

##### ^1^Kinesiology and Community Health, University of Illinois at Urbana Champaign, Champaign, IL, 61822, USA; ^2^Center for Healthful Behavior Change, New York University, New York, NY, 10016, USA; ^3^Global Institute of Public Health, New York University, New York, NY, 10003, USA; ^4^Physiology, Kwame Nkrumah University of Science and Technology, Kumasi, 00000, Ghana; ^5^Population Health and Medicine, New York University, New York, NY, 10016, USA

###### **Correspondence:** Joyce Gyamfi – Center for Healthful Behavior Change, New York University, New York, NY, 10016, USA

**Background:** Ghana has a population of 23.5 million people, of which 3.5 million adults are hypertensive. Socioeconomic factors, weak healthcare systems, uncoordinated care, and shortage of healthcare providers limit the capacity to implement and sustain interventions for hypertension control at the primary care level. However, task-shifting of primary care duties from physicians to non-physician health care providers may mitigate systems-level barriers to optimal hypertension and cardiovascular risk control in SSA. The objective of this study was to assess stakeholders’ perceptions of factors likely to influence the long-term sustainability of an on-going cluster-randomized task-shifting strategy for blood pressure control in Ghana.

**Methods:** The study used free listing exercises and focus discussion sessions to explore stakeholder perceptions of the factors likely to facilitate or limit sustainment of a task-shifting strategy in community health centers and district hospitals beyond the initial implementation period. A total of 85 stakeholders (42 patients, 27 nurses, 12 site directors, and 4 ministry of health staff) participated in this study. The resulting data were analyzed using thematic analysis techniques to identify themes.

**Findings:** Factors likely to influence the sustainability of the task-shifting strategy include the provision of adequate medications to patients as well as the training more workers using the task-shifting strategy whether as part of the nursing curriculum in pre-service training or as refresher courses for in-service training for health systems managers and other staff. Other factors include the involvement of key leaders in Ghana Health Services, labor unions, and National Health Insurance Scheme as well as lay health workers such as community health workers. Potential challenges cited by participants include lack of leadership support, cost of medications provided and staff attitude and turnover rate.

**Implications for D&I Research**: In Ghana and throughout sub-Saharan Africa, there is paucity of empirical evidence on “what it takes” to sustain evidence-based interventions and this study is uniquely positioned to examine these factors using implementation data currently being collected. Our findings address an important gap in implementation science in low resource settings by examining stakeholder’s perceptions of the factors likely to facilitate or hinder sustainment of a task-shifting strategy for blood pressure control in Ghana over time.

Primary Funding Source: National Institutes of Health.

### S36 Contextual adaptation of the consolidated framework for implementation research (CFIR) in a tobacco cessation study in Vietnam

#### Pritika Kumar^1^, Nancy Van Devanter^2^, Nam Nguyen^3^, Linh Nguyen^3^, Trang Nguyen^3^, Nguyet Phuong^3^, Donna Shelley^1^

##### ^1^Department of Population Health, New York University School of Medicine, New York, NY, 10016, USA; ^2^College of Nursing, New York University, New York, NY, 10075, USA; ^3^Institute of Social and Medical Sciences, Institute of Social and Medical Sciences, Hanoi, Vietnam

###### **Correspondence:** Donna Shelley – Department of Population Health, New York University School of Medicine, New York, NY, 10016, USA

**Background:** VQUIT is a NIH-funded cluster randomized trial that is comparing the effectiveness of two system-level strategies for implementing tobacco treatment guidelines in community health centers (CHCs) in Vietnam. There is very little literature on how Implementation Science Frameworks apply to low-middle income countries (LMICs). As part of the baseline assessment we used the Consolidated Framework for Implementation Research (CFIR) to analyze organizational-level factors that may influence implementation effectiveness and sustainability and assessed the need for cultural and contextual adaptations to the framework when applying it in LMICs.

**Methods:** We analyzed forty in-depth interviews with health providers and village health workers (VHWs) from 8 CHCs. Data included transcriptions of interviews and notes from detailed discussions with field partners. Themes were identified, discussed and revised as part of an iterative process and mapped against CFIR.

**Findings:** Many of the CFIR constructs were identified in narratives of the CHC/VHW participants, however, certain constructs related to ***intervention characteristics*** seemed to have overlapping meaning while some constructs such as *intervention source*, *adaptability* and *trialability* were redundant in part due to the lack of autonomy of the CHCs in independently deciding to adapt or pilot interventions. ***Inner setting and outer setting*-** In a culture where the national goals and priorities are set top-down, the lines between the inner and outer setting are blurred. While it is important to tap into the health providers’ perception about the ***intervention characteristics***, CHCs have limited involvement in deciding what health campaigns are implemented. ***Individual characteristics*-** CFIR focuses on individual *self-efficacy.* However, in a setting with a socialist health insurance infrastructure and political culture like Vietnam’s the construct of collective efficacy appeared more relevant to participants’ experience in implementing new programs and policies. When using the CFIR in contexts like Vietnam, we may need to construct questions that are more directed towards implementers with less individual autonomy and working in a publically financed rural health care system.

Implications for D&I Research: This paper points to the importance of contextual and ecological adaptations of frameworks to account for the reciprocity between internal, relational and external contexts when understanding the processes of an intervention’s implementation.

Primary Funding Source: National Institutes of Health - NIH R01.

### S37 Evidence check: A knowledge brokering approach to systematic reviews for policy

#### Sian Rudge

##### Knowledge Exchange Division, Sax Institute, Ultimo, NSW 2007, Australia

The first presentation on this panel will focus on the experience of the Sax Institute in Australia, which responds to health policymakers’ requests for systematic reviews through its Evidence Check program. One key characteristic of Evidence Check is the role Sax Institute staff play in brokering an ongoing conversation between the policy client and the usually academia-based research team conducting the review. In addition to assuring the reviews are completed in time to inform policymaker decisions, Sax works with the policymaker to refine the question, identify and contract with the review tea, and facilitates ongoing contact between the two groups to assure the final product is useful. Sax has recently engaged in a mixed-methods evaluation to understand the ultimate impact of Evidence Check on policy and to understand the experiences of both the policy client and review team throughout the review process. These results will serve as the basis for this presentation.

Primary Funding Source: The Robert Wood Johnson Foundation.

### S38 Using Evidence Synthesis to Strengthen Complex Health Systems in Low- and Middle-Income Countries

#### Etienne Langlois

##### Alliance for Health Policy and Systems Research, World Health Organization, Geneva, 1211, Switzerland

**Background:** Use of systematic reviews and evidence syntheses play an essential role in strengthening health systems and improving service delivery and health outcomes. Health system research (HSR) synthesis supports policy- and decision-makers by providing state-of-the-art knowledge at every step in the decision-making process. Although there is growing recognition of the importance of HSR synthesis to both inform policy decisions and produce guidance for health systems, key challenges remain around the complexity and timeliness of reviews.

**Methods:** The WHO Alliance for Health Policy and Systems Research supported the establishment of systematic review centres in low- and middle-income countries, which focused on reviews to enhance the performance of health systems. Different approaches have been implemented to foster timely engagement of decision-makers, including priority-setting exercises, innovative models of embedding policymakers in the review process, and a tool for policy-makers to identify health systems research priorities to be reviewed. The Alliance also tested two multi-site evidence-to-policy models focusing on timely use of evidence syntheses to enhance the responsiveness of health systems.

**Findings:** Approaches to engage policy- and decision-makers in HSR synthesis achieved key successes in stimulating demand and use of review findings in health systems decision-making. In Lebanon, for instance, the Alliance supported a systematic review on coordination of health services in humanitarian crisis, which served as the basis of a policy dialogue on the Syrian refugee crisis, and informed the development of a refugee health information system. In addition, the Alliance developed recommendations to advance the field of HSR synthesis, including the expansion of range of study designs to be included in reviews of health systems intervention and reforms.

Implications for D&I Research: Early engagement with decision-makers is critical for prioritizing and conducting HSR reviews, as well as enhancing the relevance and uptake of review findings. More efforts are needed globally to take stock of impact stories and develop robust and valid metrics to measure the timely use of evidence syntheses in policy and practice. There is also a need to advance the science and application of reviews on complex implementation and health systems issues, including rapid response services.

Primary Funding Source: The Robert Wood Johnson Foundation

### S39 Does it matter: timeliness or accuracy of results? The choice of rapid reviews or systematic reviews to inform decision-making

#### Andrea Tricco

##### Dalla Lana School of Public Health/St Michael's Hospital, University of Toronto, Toronto, ON, M5B 1W8, Canada

**Background:** Rapid reviews are a form of systematic review in which components of the systematic review process are streamlined to produce information in a timely manner. It is unclear whether rapid reviews are susceptible to biased results as a consequence of the streamlined methods. Although numerous rapid review programs exist internationally, few studies have examined their methodology.

**Methods:** A research program on rapid reviews was conducted, including: 1) a scoping review of rapid reviews, 2) electronic survey, and 3) consensus-building exercise (Delphi). For the scoping review, MEDLINE, EMBASE, the Cochrane Library, internet websites of rapid review producers, and reference lists were searched to identify articles for inclusion. Two reviewers independently screened literature search results and abstracted data from included studies. Descriptive analysis was conducted. An international survey of rapid review producers was conducted, and a consensus-building exercise using a modified Delphi approach.

**Findings:** We included 100 articles in our scoping review; studies failed to report between 6 % and 73 % of the specific systematic review steps examined. Fifty unique rapid review methods were identified, and 16 methods occurred more than once. Forty rapid review authors responded to our survey (63 % response rate). Eighty rapid review products with 33 different rapid review names were reported. The commissioning organizations of rapid reviews were predominantly government agencies (77 %) and healthcare organizations (59 %). The consensus-building exercise included input from 113 stakeholders on the rapid review approaches from the survey. One approach was ranked the most feasible (72 %, 81/113 responses), with the lowest perceived risk of bias (12 %, 12/103), ranking 2nd in timeliness (37 %, 38/102) and 5th in comprehensiveness (5 %, 5/100). In addition to these results, research on rapid reviews conducted by other researchers will be presented, such as policy-makers’ perspectives, decision-makers use of rapid reviews, utility of rapid reviews, and a research agenda on rapid reviews, including a prospective study to compare the reliability of results obtained through rapid reviews and systematic reviews on the same topics.

**Implications for D&I Research:** Rapid reviews might be more feasible than systematic reviews to provide important information to decision-makers in a timely manner, increasing relevance, dissemination, and implementation.

Primary Funding Source: The Robert Wood Johnson Foundation

## Health policy dissemination and implementation

### S40 Evaluation of the veterans choice program using lean six sigma at a VA medical center to identify benefits and overcome obstacles

#### Sherry Ball^1^, Anne Lambert-Kerzner^2,3^, Christine Sulc^4^, Carol Simmons^4^, Jeneen Shell-Boyd^5^, Taryn Oestreich^4^, Ashley O'Connor^4^, Emily Neely^4^, Marina McCreight^2^, Amy Labebue^6^, Doreen DiFiore^7^, Diana Brostow^8^, P. Michael Ho^2,3^, David Aron^9,10^

##### ^1^Research, Department of Veterans Affairs, Cleveland, OH, 44106, USA; ^2^Research, Eastern Colorado Health Care System, Department of Veterans Affairs, Denver, CO, 80220, USA; ^3^University of Colorado, Denver, CO, 80204, USA; ^4^Center of Innovation for Veteran-Centered and Value-Driven Care, VA Puget Sound Health Care System, Seattle, WA, 98108, USA; ^5^Research Service, Cleveland VA Medical Center, Cleveland, OH, 44106, USA; ^6^Denver-Seattle Center of Innovation, Eastern Colorado Health Care System, Aurora, CO, 80045, USA; ^7^Research, Cleveland VA Medical Center, Cleveland, OH, 44106, USA; ^8^Denver-Seattle Center of Innovation, VA Eastern Colorado Health Care System, Denver, CO, 80220; ^9^Education Department, Louis Stokes Cleveland VA Medical Center, Cleveland, OH, 44106, USA; ^10^Medicine, Case Western Reserve University School of Medicine, Cleveland, OH, USA

###### **Correspondence:** Sherry Ball – Research, Department of Veterans Affairs, Cleveland, OH, 44106, USA

**Background:** In response to the enactment of the Veterans Access, Choice, and Accountability Act (VACA) of 2014, the Veterans Health Administration (VHA) released the Veterans Choice Program (VCP) for immediate implementation in November of 2014. VACA set guidelines for all VHA medical facilities to offer non-VA healthcare to any Veteran residing over 40 miles from a VA healthcare facility or with a VA healthcare appointment scheduled 30 days or more from the clinically indicated date. The goal of VACA and the VCP is to improve access to timely healthcare for all Veterans. A national multi-disciplinary team was tasked with the evaluation of these implementation efforts. The results of this rapid evaluation at one predominantly urban VA Medical Center are presented here.

**Methods:** Using the LEAN Six Sigma (LSS) approach to define, measure, and analyze components of the VCP, we assessed the efficiency, quality, and implementation processes of the program. Key informant interviews were used to obtain staff and providers values and opinions regarding the VCP. These interviews identified the VCP process components and barriers and facilitators of implementation process. We used a Rapid Analysis technique for qualitative data analyses.

**Findings:** Process maps were created to visualize the steps to obtaining non-VA care through the VCP. Maps included the roles and responsibilities of Veterans, key informants, a patient advocate, medical support staff (MSA), clinical and administrative CCs, VA and non-VA providers, and the third party administrators. Implementation improvements were recommended for all identified practice disparities of the over twenty identified implementation process steps.

**Implications for D&I Research:** The adoption of LSS in healthcare has been slow. Less than ten studies using LSS have been published per year since 2005 when first applied to this setting. This project illustrates how LSS can facilitate the identification of challenges and successful program implementation when timelines are short.

Primary Funding Source: Department of Veterans Affairs - VA HSRD QUERI.

### S41 The influence of local context on multi-stakeholder alliance quality improvement activities: A multiple case study

#### Jillian Harvey^1,2^, Megan McHugh^3^, Dennis Scanlon^4^

##### ^1^Health Professions, Medical University of South Carolina, Charleston, SC, 29425, USA; ^2^Healthcare Leadership and Management, Medical University of South Carolina, Charleston, SC, 29425, USA; ^3^Center for Healthcare Studies, Northwestern University, Chicago, IL, 60611, USA; ^4^Department of Health Policy and Administration, Pennsylvania State University, University Park, PA, 16802, USA

###### **Correspondence:** Megan McHugh – Center for Healthcare Studies, Northwestern University, Chicago, IL, 60611, USA

**Background:** Serious problems with the quality of healthcare across the U.S. are well documented. While some providers have improved the quality of care within their organizations, these efforts have failed to translate into improved population-level outcomes. Many researchers and policymakers maintain that to achieve meaningful and sustainable improvement, quality improvement (QI) efforts need to advance from organizational-level initiatives to broader, alliance-led efforts engaging providers, payers, purchasers, and consumers. Little is known about how to take QI principles from provider settings and scale them across a community. In addition, there are many contextual factors that make a community-level approach tenuous. The purpose of this study is to learn from the efforts of 16 alliances tasked with creating community-based QI. We examine how local context impacts the implementation of QI activities and identify facilitators and barriers to disseminating QI efforts.

**Methods:** Using a multiple case study approach, we examine the evolution of QI work in the Aligning Forces for Quality (AF4Q) program. AF4Q is a ten-year and $300 million Robert Wood Johnson Foundation effort that provided alliances with funding and technical assistance to raise the quality of healthcare. The constant comparison method was used to examine the commonalities and differences across alliances in how they approached QI and the outcomes of their efforts.

**Findings:** We identified 10 alliance activities that can facilitate spreading QI principles and developing a robust community-level effort. We report evidence on how alliances tailored these activities based on local context. We conclude that while success is not guaranteed, it is possible, under certain conditions and approaches, for alliances to facilitate the spread and dissemination of QI interventions across communities.

Implications for D&I Research: Given the emphasis on local approaches to healthcare QI, the ability of alliances to adapt QI activities to local context is critical. It is important for program funders and alliance leaders to remember the local context when planning community-level QI efforts. Some alliance adaptations to QI initiatives facilitated spread better than the program-level pilots. Non-AF4Q communities can learn from the lessons and challenges of the AF4Q alliances to better understand which QI activities work best under certain conditions.

Primary Funding Source: The Robert Wood Johnson Foundation

### S42 Increasing physical activity in early care and education: Sustainability via active garden education (SAGE)

#### Rebecca Lee^1^, Erica Soltero^2^, Nathan Parker^2^, Lorna McNeill^3^, Tracey Ledoux^2^

##### ^1^College of Nursing and Health Innovation, Arizona State University, Phoenix, AZ, 85004, USA; ^2^Health & Human Performance, University of Houston, Houston, TX, 77204, USA; ^3^Health Disparities Research, The Univ. of Texas MD Anderson Cancer Center, Houston, TX, 77030, USA

###### **Correspondence:** Rebecca Lee – College of Nursing and Health Innovation, Arizona State University, Phoenix, AZ, 85004, USA

**Background:** IOM guidelines suggest young children should achieve 15 minutes of physical activity (PA) each hour in early care and education (ECE) centers. Led by a community partnership, Sustainability via Active Garden Education (SAGE) was designed using IOM PA guidelines and tested in two pilot studies (SAGE1, SAGE2) in six ECE centers.

**Methods:** Curriculum development was guided by the Ecologic Model of Physical Activity and Social Cognitive Theory. IOM guidelines, existing ECE curricula and accreditation standards were reviewed by the partnership for inclusion in the curriculum. Twelve, 1-hour modularized lessons were created using the garden as a metaphor for human development featuring songs, simple games, pretend play, modeling and garden activities. Parents were engaged via weekly newsletters. Children (M = 4 years) in SAGE1 (N = 30) and SAGE2 (N = 31) wore accelerometers before the intervention (T1), during SAGE lessons and after the intervention (T2). ECE directors (N = 4) completed exit interviews in SAGE1; parents (N = 13) completed a survey about PA and parenting practices in SAGE2.

**Findings:** In both pilot tests PA increased (SAGE1 T1 M = 9 min, during lessons M = 14 min, T2 = 11 min; p = .016; SAGE2 T1 M = 19 min, during lessons M = 29 min, T2 = 39 min; p = .009). Sedentary time decreased (SAGE1 T1 M = 50 min, during lessons M = 46 min, T2 = 45 min; p = .016; SAGE2 T1 M = 41 min, during lessons M = 21 min, T2 = 39 min; p = .010). ECEC reported that children were better behaved, appropriately hungry at snack time, and ready for sleep at nap time after SAGE because of the increase in PA. Even children who did not like exercising moved much more with the added value of the garden-focused active learning games. Parents believed that participating in SAGE improved their child’s knowledge of PA (83 %) and their own knowledge of PA (69 %). 54 % of parents reported that their child asked to do SAGE activities at home, and parenting practices that encouraged PA increased from T1 (M = 57.8) to T2 (M = 69.4) in SAGE2 (t = -2.204, p = .052).

Implications for D&I Research: SAGE partnered scientific theory and rigor with community ingenuity to create a clear translation of policy guidelines into an innovative, nutrition and physical activity curriculum that was easily implemented and engaging.

Primary Funding Source: National Institutes of Health - This work was supported by a grant (R21HD073685) from the National Institute of Child Health and Human Development (NIH) awarded to Dr. Lee.

### S43 Marking a decade of policy implementation: The successes and continuing challenges of a provincial school food and nutrition policy in Canada

#### Jessie-Lee McIsaac, Kate MacLeod, Nicole Ata, Sherry Jarvis, Sara Kirk

##### School of Health and Human Performance, Dalhousie University, Halifax, NS, B3H4R2, Canada

###### **Correspondence:** Jessie-Lee McIsaac –School of Health and Human Performance, Dalhousie University, Halifax, NS, B3H4R2, Canada

**Background:** The Nova Scotia provincial government in Canada embarked on a review and update of the 2006 school food and nutrition policy (SFNP), with a revised policy expected in 2015. The purpose of this research was to comprehensively assess the implementation of the SFNP in Nova Scotia using a multi-method approach.

**Methods:** For the first phase of our research we conducted an online menu review and gathered insight from youth using an innovative knowledge sharing project. The second phase of our research includes an assessment of the “current-state” of the original policy through three activities: 1) An online survey completed by school principals across all grade levels to gather information on the types of foods served and sold in schools, their price and promotion and factors that play a role in decision making. 2) School food environment scans to assess food preparation practices and capture photos of physical features related to food in the school that included a brief interview with both the principal and the food service staff in each cafeteria. 3) Consultation with key stakeholders (regional school food champions and food service provider organizations) and a focus group with youth to gain preliminary insight into the perceived factors that may influence the implementation of the revised SFNP.

**Findings:** Our online menu review demonstrated variability in policy adherence and non-compliance by 12-45 % of schools depending on food preparation practices. Youth shared the importance of social influences, convenience, availability and choice in their food decisions and wanted to be more engaged in decisions related to school food. Based on findings from our program of research, it is clear that school leadership is fundamental to the adherence of the SFNP as it strongly influenced the culture of schools and provided direction for values and priorities. Absence of monitoring and enforcement of the SFNP may also be a barrier to policy implementation.

**Implications for D&I Research:** This research provides an understanding of the adherence to the current SFNP, helps to inform implementation of the revised policy and builds a strong foundation for subsequent monitoring and evaluation. Moving forward, the revised SFNP needs to address barriers to its implementation. Further research will be conducted to assess the implementation of the revised SFNP and its costs.

Primary Funding Source: Nova Scotia Government (Canada)

### S44 Use of research evidence among state legislators who prioritize mental health and substance abuse issues

#### Jonathan Purtle^1^, Elizabeth Dodson^2^, Ross Brownson^3^

##### ^1^ Health Management &Policy, Drexel University School of Public Health, Philadelphia, PA, 19130, USA; ^2^ Institute for Public Health, Washington University, St. Louis, MO, 63112, USA; ^3^ Brown School and Prevention Research Center in St. Louis, Washington University in St. Louis, Saint Louis, MO, 63130, USA

###### **Correspondence:** Jonathan Purtle – Health Management &Policy, Drexel University School of Public Health, Philadelphia, PA, 19130, USA

**Background:** The importance of translating mental health (MH) and substance abuse (SA) research into policy is widely acknowledged. Information about how research evidence is used by policymakers who actively address MH/SA issues can enhance the effectiveness of research dissemination strategies and advance this goal. Few studies have investigated questions in this domain. This study’s aims were to: 1) identify factors associated with state legislators’ prioritization of MH/SA issues, and 2) describe use of research evidence practices and preferences among state legislators who prioritize these issues.

**Methods:** Between October-January 2012, we conducted a telephone-based survey of a random sample of U.S. state legislators (N = 862, response rate 50 %). Survey questions explored legislators’ individual characteristics, policy priorities, information seeking practices, and information receiving preferences. We generated descriptive statistics and conducted bivariate analyses and multivariate logistic regression to achieve our aims.

**Findings:** MH/SA issues were identified as top legislative priorities by 14.9 % of legislators. These legislators were not significantly different from those who did not prioritize these issues in terms of political ideology (e.g., liberal/conservative) or demographic characteristics. Legislators who prioritized MH/SA issues were significantly more likely to identify research evidence as one of the two factors that determine the issues they work on (32.3 % vs. 20.0 %, *p* = .002). These legislators also reported a higher usefulness rating for 10 of 12 statements about research evidence, with these differences being significant for four statements (e.g., research being presented in a “brief, concise way” [*p* = .044] and research “telling a story” [*p* = .033]). Legislators who prioritized MH/SA issues also reported attending research presentations more frequently (*p* = .044). After adjusting for covariates, legislators who identified research evidence as one of the two factors that determine the issues they work on were nearly twice as likely to prioritize MH/SA issues (adjusted odds ratio: 1.91, 95 % CI: 1.25, 2.90).

**Implications for D&I Research:** Research evidence appears to have more influence on, and be used more frequently by, legislators who prioritize MH/SA issues than legislators who do not prioritize these issues. Opportunities exist for D&I researchers to develop and evaluate dissemination strategies that target legislators who have MA/SA issues atop their legislative agendas.

Primary Funding Source: National Institutes of Health - National Institutes of Health grant NCI #1R01CA124404-01

## Models, measures, and methods

### S45 PANEL: Effectiveness-Implementation Hybrid Designs: Clarifications, Refinements, and Additional Guidance Based on a Systematic Review and Reports from the Field - Hybrid type 1 designs

#### Brian Mittman^1^, Geoffrey Curran^2^

##### ^1^Research and Evaluation, Kaiser Permanente Southern Calif/US Dept of Veterans Affairs, Pasadena, CA, 91101, USA; ^2^Department of Pharmacy Practice, University of Arkansas for Medical Sciences, Little Rock, AR, 72205, USA

###### **Correspondence:** Brian Mittman – Research and Evaluation, Kaiser Permanente Southern Calif/US Dept of Veterans Affairs, Pasadena, CA, 91101, USA

**Background:** Hybrid Type 1 effectiveness-implementation study designs incorporate exploratory implementation and pre-implementation aims within clinical effectiveness studies. The primary goal of Type 1 studies is to facilitate subsequent research, policy and practice activities designed to accelerate appropriate implementation and population benefits of health interventions found to be effective and suitable for routine use. Recent experience using Hybrid Type 1 designs in funded studies, plus related advances in implementation science theory, methods and measures, offer valuable guidance to strengthen the design, conduct and reporting of Type 1 studies.

**Methods:** We reviewed over 40 published empirical articles and study protocols presenting hybrid studies and reflected upon a large collection of hybrid-related inquiries, grant reviews, and discussions with implementation science colleagues over the past 3+ years. We abstracted key challenges, insights and recommendations related to use of Type 1 designs from published articles and our discussions, and drew from publications presenting hybrid-related frameworks and methods for data collection and analysis and published guidance for research reporting.

**Findings:** Key challenges related to Type 1 designs include uncertainty regarding selection of implementation-related variables to measure, researcher role in guiding or otherwise influencing implementation activities (conducted to enhance fidelity of implementation and researcher ability to accurately measure clinical effectiveness), and the availability of validated measures and instruments for implementation-related measurement. Recommendations and additional guidance for strengthening Hybrid Type 1 designs include relevant frameworks (e.g., RE-AIM, CFIR and additional frameworks best suited to guide selection of key implementation outcomes and independent variables for measurement in implementation-related aims of Type 1 studies) and guidance for reporting findings to maximize beneficial use by subsequent researchers and practitioners.

Implications for D&I Research: Systematic assessment and description of published Hybrid Type 1 studies leading to enhanced guidance for research designs, methods, measures and analytic approaches for this category of study design will facilitate more efficient, higher quality data collection and analysis. Implementation research will proceed more rapidly and more effectively if researchers conducting clinical effectiveness and comparative effectiveness research include implementation-related elements as recommended by the Hybrid Type 1 design, ultimate contributing to better implementation and greater impact and benefits of health research.

### S46 PANEL: Effectiveness-Implementation Hybrid Designs: Clarifications, Refinements, and Additional Guidance Based on a Systematic Review and Reports from the Field - Hybrid type 2 designs

#### Geoffrey Curran

##### Department of Pharmacy Practice, University of Arkansas for Medical Sciences, Little Rock, AR, 72205, USA

**Background:** Hybrid Type 2 designs allow simultaneous assessment of clinical effectiveness of a clinical practice and implementation effectiveness of a practice change (or “uptake”) strategy. These designs facilitate rapid progress through the “research-implementation-impact” pipeline through concurrent research activity examining issues that have traditionally been explored separately. Published Type 2 studies/protocols and discussions with researchers have identified a series of challenges, solutions, and recommendations for improving validity, efficiency, and overall value of Type 2 designs.

**Methods:** We reviewed over 40 published empirical articles and study protocols presenting hybrid studies and reflected upon a large collection of hybrid-related inquiries, grant reviews, and discussions with implementation science colleagues over the past 3+ years. We abstracted key challenges, insights and recommendations related to use of Hybrid 2 designs from published articles and our discussions, and drew from publications presenting hybrid-related frameworks and methods for data collection and analysis and published guidance for research reporting.

**Findings:** Type 2 designs pose special challenges related to selection of outcomes, sample size, randomization, and reporting. Factorial designs offer a well-established solution permitting simultaneous randomization and comparison of clinical interventions alongside implementation strategies, but do not offer definitive guidance for decisions on unit of analysis, randomization, and clustering. Resource challenges are particularly problematic in Type 2 studies of complex clinical interventions (e.g., behavioral interventions) for which process evaluation and measurement of mediators, moderators, and mechanisms are required to answer both clinical and implementation questions. Recommendations and guidance to strengthen Type 2 designs include tables listing illustrative: sampling, randomization and unit-of-analysis decisions; study designs representing alternatives to factorial design; and analysis strategies, including recommendations for studies in which clinical effectiveness interacts with (is dependent on) implementation fidelity.

**Implications for D&I Research**: Review of recent Type 2 Hybrid effectiveness-implementation studies reveals a rich array of solutions to common challenges in design, methods, measures and analysis; however, substantial challenges remain to be considered. The goal of accelerating progress toward implementation and impact of innovative clinical interventions is more readily achieved if researchers interested in simultaneous pursuit of effectiveness and implementation follow “best practices” in the design, conduct, and reporting of Type 2 studies.

### S47 PANEL: Effectiveness-Implementation Hybrid Designs: Clarifications, Refinements, and Additional Guidance Based on a Systematic Review and Reports from the Field - Hybrid type 3 designs

#### Jeffrey Pyne

##### Psychiatry and Behavioral Sciences, University of Arkansas for Medical Sciences, Little Rock, AR, 72205, USA

**Background:** Hybrid Type 3 designs represent implementation-focused studies with the added measurement of variables assessing effectiveness of the underlying clinical intervention. These designs are indicated when evidence of clinical effectiveness is “incomplete” (or of limited relevance to the settings of interest in the new study) and when clinical effectiveness is likely highly dependent on implementation fidelity/quality. Additional guidance is required to determine how and when to measure clinical outcomes and how to analyze clinical effectiveness in the context of a study powered and designed to answer implementation questions.

**Methods:** We reviewed over 40 published empirical articles and study protocols presenting hybrid studies and reflected upon a large collection of hybrid-related inquiries, grant reviews, and discussions with implementation science colleagues over the past 3+ years. We abstracted key challenges, insights and recommendations related to use of Hybrid 3 designs from published articles and our discussions, and drew from publications presenting hybrid-related frameworks and methods for data collection and analysis and published guidance for research reporting.

**Findings:** Key Type 3 challenges include analytic problems related to the interplay between implementation outcomes and clinical effectiveness, particularly for complex clinical interventions for which contextual influences and implementation factors represent strong influences on clinical outcomes. Advances in observational study designs and methods to address selection bias, confounding, and other validity threats offer useful strategies to strengthen clinical effectiveness aims in these studies. Recently-published Type 3 studies offer useful models for selection of clinical outcome measures (including strategies for using increasingly prevalent secondary data) and other key design and methods decisions. Recent contributions in reporting guidelines offer useful guidance for reporting implementation and effectiveness design features and findings. Recent publications and discussions with researchers offer additional guidance in deciding when to conduct a Type 3 hybrid vs. a conventional implementation study.

Implications for D&I Research: Although resource limitations sometimes preclude inclusion of clinical effectiveness measures in all implementation studies, new guidance in design, methods, and measurement may help increase the number of implementation-focused studies including clinical effectiveness aims. Evidence suggesting that clinical effectiveness can vary with implementation success highlights the importance of measuring clinical effectiveness whenever possible.

### S48 Linking team level implementation leadership and implementation climate to individual level attitudes, behaviors, and implementation outcomes

#### Gregory Aarons^1^, Mark Ehrhart^2^, Elisa Torres^1^

##### ^1^Psychiatry, UC San Diego, La Jolla, CA, 92083-0812, USA; ^2^Department of Psychology, San Diego State University, San Diego, CA, 92182-4611, USA

###### **Correspondence:** Gregory Aarons – Psychiatry, UC San Diego, La Jolla, CA, 92083-0812, USA

**Background:** In healthcare and allied healthcare settings, leadership and climate that supports effective implementation of evidenced-based practices (EBPs) is critical. While recent research has developed measures to assess implementation leadership, climate, and citizenship behaviors (i.e., Implementation Leadership Scale [ILS]; Implementation Climate Scale [ICS]; Implementation Citizenship Behavior Scale [ICBS]), models have yet to examine the nature of their relationships with individual level provider attitudes, behaviors, and implementation outcomes. This study advances implementation science by testing a cross-level model examining the relationships between team level implementation leadership and climate, and how they relate to provider attitudes, implementation citizenship behaviors, and implementation success. Based on our cross-level conceptual model we hypothesized: a) a positive association between leadership and implementation climate, b) a positive significant cross-level association of implementation climate and provider attitudes toward EBP, c) implementation climate would mediate the relationship of leadership and provider attitudes toward EBP, and d) implementation citizenship behaviors would mediate the relationship of attitudes implementation outcomes.

**Methods:** Participants were 60 clinical supervisors and 371 providers from mental health service organizations. Providers completed the ILS, ICS, and Evidence Based Practice Attitude Scale (EBPAS). Supervisors completed the ICBS for each provider on their team, and rated each provider’s implementation success. Multilevel path analysis, accounting for the nested data structure (i.e., providers nested within teams [k = 79]) examined cross-level and mediation effects. Remediation assessing confidence intervals for mediated effects was used to examine indirect effects.

**Findings:** Results provided support for positive relationships in the multilevel path model linking implementation leadership to implementation success. Specifically, we found significant positive relationships between team level implementation leadership and implementation climate, cross-level effects between implementation climate and provider attitudes toward EBP, provider attitudes toward EBP and implementation citizenship behaviors, and implementation citizenship behaviors and implementation success. Results also supported the presence of several hypothesized mediational relationships.

**Implications for D&I Research:** This study advances our understanding of the multilevel nature and process of how leadership and organizational context may impact provider attitudes toward EBPs, subsequent provider behaviors to support EBP implementation, and implementation outcomes. Findings suggest several factors that organizations can address during implementation to support efforts to improve implementation process and success.

Primary Funding Source: National Institutes of Health - R21MH098124; R01MH072961.

### S49 Pinpointing the specific elements of local context that matter most to implementation outcomes: Findings from qualitative comparative analysis in the RE-inspire study of VA acute stroke care

#### Edward Miech

##### HSR&D, Richard L. Roudebush VA Medical Center, Indianapolis, IN, 46202, USA

**Background:** Qualitative Comparative Analysis (QCA) derives from applied set theory and allows researchers to pinpoint specific combinations of conditions that connect directly to outcomes. The RE-INSPIRE study – a longitudinal four-year, in-depth observational study of the organization of acute stroke care at 11 sites within the VA – applied QCA to generate new insights into which elements of local context mattered most to implementation outcomes.

**Methods:** The Consolidated Framework for Implementation Research (CFIR) served as the study’s conceptual framework. From 2012-2015 the RE-INSPIRE study conducted three in-person annual site visits at 11 participating VA medical centers to study the organization of acute stroke care at each facility, including changes over time. Semi-structured interviews with providers at each facility were audiotaped and professionally transcribed. An 8-person team met to score 22 different CFIR constructs for each of the 33 site visits, assigning valence and magnitude ranging from -2 to +2. Team members voted on individual CFIR scores via digital secret ballot; decisions had to be unanimous. A similar approach was used to assign Group Organization scores (i.e., GO Scores) ranging from 1 to 10 to determine the level at which VA staff organized themselves to provide or improve acute stroke care at their facility. Over 700 CFIR values and 60 GO Scores were developed using this approach. Crisp-set Qualitative Comparative Analysis (i.e., csQCA) was used to identify the specific components of local context that individually and in combination were uniquely associated with level of organization.

**Findings:** Solution coverage was 100 %; csQCA solutions accounted for all cases. The level (advanced, middle, low) at which local VA staff organized themselves to provide or improve acute stroke care at their facility could be explained with only 3 CFIR constructs (out of a total of 22 CFIR constructs). The influence of the CFIR construct Reflecting & Evaluating on the level at which acute stroke care was organized resembled a dose-response curve.

**Implications for D&I Research:** QCA findings in RE-INSPIRE indicate that engaging groups of providers in Reflecting & Evaluating is a key driver of implementation for any VA medical center trying to develop and improve its facility-wide acute stroke program.

Primary Funding Source: Department of Veterans Affairs - National VA QUERI Program.

### S50 The GO score: A new context-sensitive instrument to measure group organization level for providing and improving care

#### Edward Miech

##### HSR&D, Richard L. Roudebush VA Medical Center, Indianapolis, IN, 46202, USA

**Background:** Albeit complex, the ways in which groups and teams organize themselves to develop and implement local solutions to improve patient care is a key element of local context that can directly influence implementation success yet vary greatly across sites. The RE-INSPIRE study – a longitudinal four-year, in-depth observational study of the organization of acute stroke care at 11 sites within the VA – developed and refined a new measure of “group organization” level called the “GO Score” to reflect the high degree of observed variation in how VA staff organized themselves to provide or improve acute stroke care at their facility.

**Methods:** From 2012-2015 the RE-INSPIRE study conducted three in-person annual site visits at 11 participating VA medical centers to study the organization of acute stroke care at each facility, including changes over time. Semi-structured interviews with providers at each facility were audiotaped and professionally transcribed. RE-INSPIRE project team members met as a group to score each of the 11 VA medical centers twice at the time of each site visit, once for how local staff at the site organized themselves to provide acute stroke care and another for how they organized themselves to improve acute stroke care. Each score corresponded to a 10-point continuum ranging from 1 to 10 that spanned beginning, basic, intermediate and advanced group organization levels.

**Findings:** A total of 66 GO Scores were assigned in the RE-INSPIRE study. Wide variation in GO Scores was observed across sites, while at individual sites observable movement from one level to another occurred during the course of the RE-INSPIRE study. GO Scores provided valuable data for multiple analyses in RE-INSPIRE, including Qualitative Comparative Analysis.

**Implications for D&I Research:** The GO Score provides a new, standardized instrument to measure cross-site variation and within-site change over time in how local staff organize themselves to provide and improve care, a contextual element that is often important in implementation research but difficult to capture. This approach was developed with generalizability in mind and can be applied to other clinical areas and contexts both inside and outside the VA.

Primary Funding Source: Department of Veterans Affairs - National VA QUERI Program

### S51 A research network approach for boosting implementation and improvement

#### Kathleen Stevens, I.S.R.N. Steering Council

##### Improvement Science Research Network, University of Texas Health Science Center San Antonio, San Antonio, TX, 78229, USA

###### **Correspondence:** Kathleen Stevens – Improvement Science Research Network, University of Texas Health Science Center San Antonio, San Antonio, TX, 78229, USA

**Background:** Health professionals are responding to demands for implementing well-tested improvement strategies in healthcare. As scientists and clinicians deal with urgent issues, a multitude of improvement projects are being conducted. Yet this collective effort falls short of producing solid evidence about which improvement strategies work and how to spread the innovation in a systems context. The challenges are to connect local projects across multiple sites, align efforts with stakeholder priorities, ensure strong study designs, achieve timely completion to support effective implementation. This approach produces a rapid learning health research enterprise that contributes to the National Quality Strategy.

**Methods:** Creation of a national improvement research network took place in response to an NIH call for infrastructures to advance new fields of science. Rapid cycle studies of implementation and performance improvement strategies were conducted in settings that were eager to implement the results, boosting adoption. Effectiveness of the research network was evaluated through the conduct of 2 landmark studies. National projects were connected virtually through the network nexus to generate generalizable evidence-based solutions at an unprecedented speed.

**Findings:** Network demonstration projects involved 34 sites and over 70 affiliates in research collaboratives. Over 24,000 data points were gathered in 9 months. Relevance was assured through national consensus on stakeholder priorities. Rigor was effectively supported by the network coordinating center, using well-developed strategies and tools for collaborative research. Members in the virtual research collaboratives indicated high satisfaction including: Enthusiasm for engagement in rigorous research; broad national representation; clinical relevance; regulatory-IRB efficiency; rapid deployment and completion; and scale up and spread of the findings.

**Implications for D&I Research**: Research collaborative members confirmed that the research network is suitable for implementation studies, producing research that is rapid, relevant, and rigorous. The network produced high-impact evidence for transforming healthcare that was readily adopted into practice.

Primary Funding Source: National Institutes of Health - NIH ARRA Grant; CTSA Grant.

### S52 PANEL: Qualitative methods in D&I Research: Value, rigor and challenge - The value of qualitative methods in implementation research

#### Alison Hamilton^1,2^

##### ^1^HSR&D Center for the Study of Healthcare Innovation, Implementation and Policy, Department of Veterans Affairs, Los Angeles, CA, 90073, USA; ^2^UCLA Department of Psychiatry and Biobehavioral Sciences, UCLA, Los Angeles, CA, 90024, USA

**Background:** Implementation research offers unprecedented opportunities to capitalize on the value of qualitative methods to illuminate organizational processes, relationships, and contexts as well as individual knowledge, attitudes, and beliefs about the change dynamics inherent in implementation. This presentation considers instrumental, conceptual, and symbolic uses of qualitative research, providing examples from implementation research studies. Concepts of value and credibility will be exemplified with regard to study design, execution, and dissemination.

**Methods:** To illustrate the value of qualitative methods, we draw from two multisite implementation studies: a VA HSR&D-funded study in women Veterans’ primary care, and an NIMH-funded study in community-based organizations serving HIV-positive adults. Longitudinal qualitative methods in these ongoing studies include individual interviews, observation, and content analysis of study-related documents. Mixed methods study designs accommodate a substantial and consistent contribution from qualitative methods.

**Findings:** In both studies, qualitative findings play a critical role in informing the implementation strategies, with careful and systematic attention to local tailoring based on interim results that are delivered to stakeholders in efficient, user-friendly formats. For example, in the VA women’s health study, baseline key stakeholder interview (n = 89 across 12 sites) results informed intervention sites’ evidence-based quality improvement targets and change processes. In the community-based implementation study, baseline interviews (n = 15 across 4 sites) as well as observation at 10 sites and content analysis of field notes informed site-specific implementation strategies to promote uptake of the evidence-based intervention and execution of the “real-world” dynamic roll-out study design. In both studies, qualitative findings have to be produced in a rapid timeframe, they need to be “actionable,” and they have to be presented in formats that are accessible to stakeholders across multiple organizational levels.

Implications for D&I Research: Given the exponential growth of implementation research, and qualitative methods therein, guidance is needed on realizing and communicating the value of this methodological orientation. This includes how to synthesize, present, and share findings in order to maximize value in terms of accessibility, relevance, and impact.

Primary Funding Source: National Institutes of Health.

### S53 PANEL: Qualitative methods in D&I Research: Value, rigor and challenge - Learning evaluation: The role of qualitative methods in dissemination and implementation research

#### Deborah Cohen

##### Family Medicine, Oregon Health & Science University, Portland, OR, 97239, USA

**Background:** In healthcare change interventions, on-the-ground learning about the implementation process is often lost because of a primary focus on outcome improvements. This paper describes the Learning Evaluation approach, and the role that qualitative methods play is studying healthcare innovations.

**Methods:** We describe the Learning Evaluation is an approach to multi-organization assessment. This is an approach that blends qualitative and quantitative methods. There are five principles at the foundation of this approach: (1) gather data to describe changes made by healthcare organizations and how changes are implemented; (2) collect process and outcome data relevant to healthcare organizations and to the research team; (3) assess multi-level contextual factors that affect implementation, process, outcome, and transportability; (4) assist healthcare organizations in using data for continuous quality improvement; and (5) operationalize common measurement strategies to generate transportable results.

**Findings:** We use Learning Evaluation as a framework and data from Advancing Care Together (ACT) –a demonstration project funded by The Colorado Health Foundation to implement integrated care delivery in primary care and community mental health centers – to demonstrate the critical role the collection of qualitative plays in real-time assessment of implementation. We describe three types of qualitative data we collected in ACT: online diary, observation and interview data. We show how we weave the collection of these data into the evaluation of an implementation research study at baseline, to understand starting conditions and key intervention elements, during the implementation and change process to identify how changes are implemented, and the factors that affect change, and following implementation of the change to make sense outcomes. We highlight the critical role qualitative data play in assessment of context, facilitating quality improvement by combining qualitative feedback and data from run charts, and in generating transportable lessons, and demonstrate how qualitative data collection adds rigor to implementation research.

Implications for D&I Research: Qualitative methods can help researchers evaluating change initiatives and organizations implementing improvements generate systematic and rigorous cross-organizational findings about implementing healthcare innovations while also enhancing organizational capacity and accelerating translation of findings by facilitating continuous learning within individual sites.

Primary Funding Source: National Institutes of Health.

### S54 PANEL: Qualitative methods in D&I Research: Value, rigor and challenge - Qualitative methods in D&I research

#### Deborah Padgett

##### Silver School of Social Work, New York University, New York, NY, 10003, USA

**Background:** Qualitative methodology is widely used in dissemination and implementation (D&I) research, yet little attention has been given to specific challenges for its use in implementation, posing difficulties to achieving and maintaining rigor from conceptualization to data collection and analysis. This presentation will illustrate and discuss some of these issues and challenges in order to begin a conversation about strengthening the use of qualitative methods in D&I research.

**Methods:** We refer to the “My Own Health Report” (MOHR) project to illustrate these issues. The MOHR project was a one-year multi-site trial implementing an evidence based health risk assessment, (the MOHR tool), in primary care using a mixed methods approach. Employing the RE-AIM framework, the trial sought to describe the reach of the intervention (the assessment and consequent goal-setting) among other outcomes. This presentation focuses on the qualitative methods used to characterize the contexts of the nine practice sites and the processes involved in implementation. The qualitative inquiry was based on an approach described in a 2013 AHRQ publication on context in health research. Data were collected at the beginning, interim and end of the implementation. A coding scheme was developed and an analysis of data was conducted with the support of Atlas.ti software.

**Findings:** The qualitative assessment met multiple challenges, including data collection in busy clinical practices and the intensive and iterative nature of qualitative methods, compared to relatively rapid quantitative approaches. Taking the MOHR project as a starting point, we will discuss the effects of these and other challenges, on rigor, reproducibility and generalizability in D&I research as it extends its reach.

Implications for D&I Research: As D&I matures, systematic consideration needs to be given to the field’s capacity to employ qualitative methods with rigor and relevance. As well, international D&I research will need to draw on qualitative methods as optimal in cross-cultural settings. Without this broad discussion and methodological development, D&I growth can be impeded by the use of less than robust qualitative findings in its research.

Primary Funding Source: National Institutes of Health

### S55 PANEL: Maps & models: The promise of network science for clinical D&I - Hospital network of sharing patients with acute and chronic diseases in California

#### Alexandra Morshed

##### Brown School, Washington University in St. Louis, Saint Louis, MO, 63130, USA

**Background:** Patient-sharing between hospitals has implications for prioritization of resources and efforts to disseminate evidence-based practice, coordinate care, and control spread of infectious diseases. We used social network analysis to map the structure of hospital patient-sharing networks for diagnoses of acute and chronic diseases to explore potential implications for dissemination of evidence-based practice. We compared the differences in network structure of hospitals treating patients with obesity and those treating patients with acute stroke.

**Methods:** This study used the State Inpatient Database (SID) from the Healthcare Cost and Utilization Project (HCUP). The SID data contain discharge information from inpatient visits to hospitals from participating states. The 2010 California SID dataset was used to create a patient-sharing network, where the network nodes represent hospitals (419 treating obese and 339 treating stroke patients), and ties between nodes represent patients shared between two hospitals. Disease-based networks were created by using principal and secondary diagnosis codes.

**Findings:** Both networks reveal a geographic distribution of hospitals. The stroke network was less dense (0.016) with a shorter diameter (13) than the obesity network (0.027 and 96, respectively). The stroke network had nearly double the centralization score (0.22 versus 0.13), suggesting a greater core-periphery structure. The stroke network more closely demonstrated a power law distribution. Together these results suggest that the stroke patient-sharing network has a core-periphery structure, with few centralized cores connected to many hospitals in the periphery. In contrast, the obese patient-sharing network has a more distributed and less hierarchical structure.

**Implications for D&I Research:** Relative location of hospitals in the network (e.g., their isolation or central position) and the network cohesion may shape decisions about dissemination of evidence-based practice and patient care planning. Inclusion of these metrics in health care planning and administration has the potential to maximize scarce resources and improve quality of care. These maps suggest that dissemination strategies for both types of disease would be best prioritized in network hubs (nodes with high betweenness scores). Dissemination activities may be slower and costlier when targeting obese patients based on the greater number of hubs and more distributed structure of the obese patient network.

### S56 PANEL: Maps & models: The promise of network science for clinical D&I - The use of social network analysis to identify dissemination targets and enhance D&I research study recruitment for pre-exposure prophylaxis for HIV (PrEP) among men who have sex with men

#### Rupa Patel

##### Division of Infectious Diseases, Washington University School of Medicine, St. Louis, MO, 63110, USA

**Background:** The human immunodeficiency virus (HIV) can cause death and suffering. New HIV infections in the U.S. are preventable, but despite many efforts the rate has not declined over 10 years. As a result, pre-exposure prophylaxis for HIV (PrEP), the use of anti-HIV medications in high-risk persons to prevent infection, has been incorporated into the national Centers for Disease Control and Prevention HIV prevention guidelines. Little is known regarding successful PrEP implementation among young men who have sex with men (MSM), in whom HIV incidence is rising. We used social network analysis (SNA) to identify PrEP dissemination networks and enhance D&I research recruitment in order to guide PrEP implementation efforts in St. Louis, Missouri.

**Methods:** We surveyed 24 MSM who were 18-35 years old and had reported recent high-risk sexual behavior. We asked the names of venues (virtual or physical) that led to condomless sex over the prior 12 months and the frequency that they were visited. These data were organized into a two-mode affiliation network that allowed identification of central venues in the network. The study was approved by the Washington University in St. Louis Institutional Review Board.

**Findings:** Participant median age was 28 years, 41 % (n = 10) were non-white or multiracial and 79 %% (n = 19) were self-reported HIV negative. Grindr, a social smartphone app, had the highest centrality within the condomless sexual encounter network. There were no differences in venue affiliation network analysis among race. After placing study recruitment advertisements on Grindr, we increased study enrollment by 29 %.

**Implications for D&I Research:** In the context of HIV prevention dissemination and implementation research, an SNA approach allowed for identification of venues of high-risk behaviors. This translated into an effective study recruitment strategy and identification of PrEP dissemination program targets that will be efficacious and cost-effective. Our findings can aid D&I researchers facing similar study recruitment and implementation challenges.

### S57 PANEL: Maps & models: The promise of network science for clinical D&I - Network and organizational factors related to the adoption of patient navigation services among rural breast cancer care providers

#### Beth Prusaczyk

##### Brown School, Washington University in St. Louis, Saint Louis, MO, 63110, USA

**Background:** In rural areas, women face inadequate access to health services (e.g. low provider:patient ratios, long distances between providers, poor public transit), which results in clinically significant delays across the breast cancer continuum. Patient navigation (PN) services have the potential to address these barriers by providing social support and education to patients, coordinating services among providers, and linking patients with resources across organizations. Little is known about the organizational and network factors that influence adoption of PN services.

**Methods:** Social network analysis (SNA) and exponential random graph modeling (ERGM) were used to understand the breast cancer care provider network in rural Missouri. Organizational leaders were surveyed from 77 Federally-Qualified Health Centers (FQHC) and Rural Health Centers (RHC) in 10 counties. Information was obtained on the extent of PN services adoption for each Center, type of center (FQHC or RHC), the county-level location of the Center, and the providers they routinely refer patients to for diagnostic mammograms or biopsies.

**Findings:** Response rate was 61 % (N = 47). The network consisted of 47 Centers and 23 referral providers. An affiliation network was created in which Centers were connected if they referred patients to at least one of the same providers. ERGM was used to measure factors related to the likelihood of a tie between two Centers. Extent of adoption was not significantly associated with the likelihood of a tie between two Centers. Centers of the same type were significantly *less* likely to be connected (OR = 0.50, 95 % CI 0.38-0.63, *p* < .0001). Centers in the same county were significantly *more* likely to be connected (OR = 5.37, 95 % CI 3.42-8.43, *p* < .0001).

**Implications for D&I Research:** The extent to which Centers have adopted PN services is not associated with sharing referral providers. This suggests that referral patterns in rural Missouri do not influence the adoption of PN services. Centers in the same county are five times more likely than centers in different counties to refer patients to the same providers. Organizational networks can facilitate dissemination of health information through connections between organizations; therefore, future strategies to implement interventions to improve cancer control or disseminate guidelines should target organizations at the county-level rather than type of center.

### S58 A theory of de-implementation based on the theory of healthcare professionals’ behavior and intention (THPBI) and the becker model of unlearning

#### David C. Aron^1,2^, Divya Gupta^3^, Sherry Ball^4^

##### ^1^Case Western Reserve University, Cleveland, OH, USA; ^2^Medicine, Louis Stokes Cleveland Medical Center, Department of Veterans Affairs, Cleveland, OH, 44106, USA; ^3^Research, Cleveland VA Medical Center, Cleveland, OH, 44106, USA; ^4^Research, Department of Veterans Affairs, Cleveland, OH, 44106, USA

###### **Correspondence:** David C. Aron – Case Western Reserve University, Cleveland, OH, USA

**Background:** “Unlearning” occurs when an outmoded practice is abandoned in favor of learning and substituting a new practice, e.g., abandoning hormonal therapy for heart disease prevention. While sometimes abandoned quickly, outmoded practices tend to persist. Correction of underuse appears easier than overuse. New conceptual models are needed that promote unlearning. Diabetes overtreatment exemplifies this need. The importance of glycemic control in preventing complications has been stressed for decades in the academic literature and continuing medical education supported by professional societies, advocacy groups, and pharmaceutical manufacturers. Practice guidelines reflect the interests of these and other stakeholders. However, many patients receive intensive treatment with little potential for benefit, but increased risk for hypoglycemia. In contrast to implementation of a new practice which may involve addition to or simple substitution for an old practice, de-implementation involves practice reversal, and in addition to learning, requires deliberate “unlearning,” and a major change in one’s mental model (deep unlearning). Unlearning requires changes in one’s knowledge and beliefs about consequences to both patient and clinician. Although there are studies of individual unlearning by managers/administrators, there are few studies involving physicians.

**Methods:** A model based on the Theory of Healthcare Professionals’ Behavior and Intention (THPBI) that incorporates the Becker Model of Unlearning (from the organizational behavior literature) is proposed to guide research and intervention development.

**Findings:** The integrated THPBI/Becker model is illustrated using the example of VA’s efforts to de-intensify glycemic control to prevent hypoglycemia in vulnerable diabetes mellitus patients. The challenges in implementing the Choosing Wisely/Hypoglycemia Safety national campaign and results from semi-structured interviews with primary care providers are discussed.

**Implications for D&I Research:** More effective intervention targets and strategies are required to promote de-implementation as evidenced by the modest reduction in diabetes overtreatment in the past five years. Conceptual models based on mixed methods can be used to identify such targets and assist in intervention design. The individual, social, and organizational aspects of unlearning require elucidation; different problems will require different proportions of learning and unlearning. Finding the right balance will be a change for both research and practice.

Primary Funding Source: Department of Veterans Affairs - VA HSRD QUERI.

### S59 Observation of registered dietitian nutritionist-patient encounters by dietetic interns highlights low awareness and implementation of evidence-based nutrition practice guidelines

#### Rosa Hand^1^, Jenica Abram^1^, Taylor Wolfram^2^

##### ^1^Research, International, and Scientific Affairs, Academy of Nutrition and Dietetics, Chicago, IL, 60606, USA; ^2^Web Strategy, Academy of Nutrition and Dietetics, Chicago, IL, 60606, USA

###### **Correspondence:** Jenica Abram – Research, International, and Scientific Affairs, Academy of Nutrition and Dietetics, Chicago, IL, 60606, USA

**Background:** The Academy of Nutrition and Dietetics’ Evidence-Based Nutrition Practice Guidelines are the foundation of dietetics practice. However, for any profession, self-report is an inadequate measure of implementation in clinical practice. Observation has been used to obtain guideline implementation data, but requires the addition of a researcher/spectator to the encounter. Dietetic interns regularly observe their preceptors, presenting a natural opportunity to gather data on guideline use without the presence of an additional researcher.

**Methods:** Five dietetic internship programs participated in a pilot project. Interns observed registered dietitian nutritionist (RDN)-patient encounters using one of four checklists based on the Academy of Nutrition and Dietetics Evidence-Based Nutrition Practice Guidelines for Critical Illness (CI), Diabetes Mellitus (DM), Disorders of Lipid Metabolism (DLM), and Unintended Weight Loss (UWL). Interns interviewed the RDN after each encounter to determine whether the dietitian believed s/he had used an Academy guideline. Analysis was descriptive; the mean percent of checklist actions observed per encounter was calculated for each guideline. The percent of RDNs who believed they had used a guideline during each encounter (based on interview) was also calculated.

**Findings:** 166 observations were recorded at 25 facilities; 10 % on DLM, 28 % on DM, 28 % on CI and 33 % on UWL guidelines. 83 % of encounters were with inpatients and 66 % were initial visits. The guideline with the highest mean percent of actions observed was UWL (43.1 ± 13.8 %) and the lowest was DLM (22.2 ± 9.5 %). For DM, CI, and UWL, RDNs reported they had used an Academy guideline 55-58 % of the time. This was lower for DLM at 35.7 %. Interview themes included: confusion between clinical protocols and practice guidelines, confusion between guidelines released by different organizations, and low awareness of specific guideline content.

**Implications for D&I Research:** Health professionals in training can collect data during encounters that they are already observing and obtain information about decisions practitioners make about implementing guidelines. This is an opportunity to gather information about guideline implementation and familiarize new professionals with specific guideline recommendations. Much more work needs to be done in disseminating guidelines and differentiating evidence-based and non-evidence based guidelines.

### S60 Program sustainability action planning: Building capacity for program sustainability using the program sustainability assessment tool

#### Molly Hastings, Sarah Moreland-Russell

##### Center for Public Health Systems Science, Washington University in St. Louis, St. Louis, MO, 63112, USA

###### **Correspondence:** Molly Hastings – Center for Public Health Systems Science, Washington University in St. Louis, St. Louis, MO, 63112, USA

**Background:** While there is a growing interest in program sustainability (the ability to maintain programming and its benefits over time), relatively few practical training curricula have been developed to support programs to build sustainability capacity. The Program Sustainability Framework and Program Sustainability Assessment Tool, which were built through a comprehensive literature review, expert input and concept mapping and were designed to capture the wide-ranging and complicated factors that impact sustainability, were used in combination with Kolb’s Experiential Learning Model (ELM) to develop and implement a sustainability action planning curriculum.

**Methods:** The Program Sustainability Assessment Tool (PSAT), a 40-item reliability-tested assessment of a program’s capacity for sustainability, was administered to multiple staff within four state-level tobacco control programs. These four programs then participated in Program Sustainability Action Planning based on Kolb’s ELM which included a one-day in person training and follow up technical assistance designed to 1.) support the program in defining all necessary programmatic factors (i.e., mission/vision, target audience, and program elements to be sustained); 2.) review and understand PSAT results, and 3.) strategically translate the PSAT results into a Program Sustainability Action Plan. Programs were encouraged to implement the action plan, update/revise as needed, and re-assess capacity using the PSAT annually to evaluate progress.

**Findings:** Initial findings indicate improvement in program sustainability capacity 12 months following Program Sustainability Action Planning across all four programs assessed. Individual domain scores fluctuated. Follow-up qualitative interviews with state program leaders indicated that plan implementation was impacted by a number of internal and external factors.

Implications for D&I Research: Program sustainability capacity-building curricula are needed, and the Program Sustainability Assessment and Action Planning Curriculum represents one of the first methods showing some efficacy. Interest in the Curriculum has broadened beyond public health and is now used by hospitals, clinical care, education, and social service programs. Important next steps include determining the predictive validity of the PSAT and verifying generalizability of the Curriculum beyond traditional public health programs.

Primary Funding Source: Centers for Disease Control and Prevention - CDC Office on Smoking and Health provides support for the Center for Public Health Systems Science to research factors related to program sustainability and support state Tobacco Control programs to build their sustainability capacity over time.

**Table 2 (Abstract S60):** State TC Program PSAT Scores

State	2013	2015	Change
State A	4.5	5.0	+.5
State B	3.6	4.5	+.9
State C	3.5	3.9	+.4
State D	4.5	4.7	+.2

### S61 A review of D&I study designs in published study protocols

#### Rachel Tabak^1^, Alex Ramsey^2^, Ana Baumann^3^, Emily Kryzer^3^, Katherine Montgomery^3^, Ericka Lewis^3^, Margaret Padek^1^, Byron Powell^4^, Ross Brownson^1,2^

##### ^1^Brown School and Prevention Research Center in St. Louis, Washington University in St. Louis, Saint Louis, MO, 63130, USA; ^2^School of Medicine, Washington University in St. Louis, St. Louis, MO, 63110, USA; ^3^Brown School, Washington University in St. Louis, St. Louis, MO, 63110, USA; ^4^Health Policy & Management, The University of North Carolina at Chapel Hill, Chapel Hill, NC, 27566-7411, USA

###### **Correspondence:** Rachel Tabak – Brown School and Prevention Research Center in St. Louis, Washington University in St. Louis, Saint Louis, MO, 63130, USA

**Background:** The need for novel study designs in dissemination and implementation (D&I) research is increasingly recognized. Despite the wide range of study designs recommended for D&I research, we lack understanding of the types of designs and methodologies that are routinely used in the field. This review addresses this gap by assessing the designs and methodologies in recently proposed D&I studies.

**Methods:** We reviewed 224 study protocols published in the journal *Implementation Science* from 2/22/2006 to 03/07/2014. A data extraction form facilitated the coding of the following design elements: design category (e.g., randomized, observational); design type (e.g., cluster randomized controlled trial (RCT), interrupted time series); data type (e.g., quantitative, mixed methods); conceptual framework or theory; levels of randomization, intervention, and measurement; and country in which the research was conducted. Each protocol was double-coded, and the small number of discrepancies were resolved through discussion with the entire study team.

**Findings:** Of the 224 protocols reviewed, 112 (50 %) tested one or more implementation strategy, therefore meeting inclusion criteria. Of the 112 included, 92 (83 %) utilized randomized designs, primarily RCTs. Only 13 studies (12 %) were quasi-experimental, and fewer (6, 5 %) were observational. There was considerable variation in the way authors described study designs. Many study design categories (e.g., controlled pre-post, matched pair cluster design) were represented by only one or two studies. Fifty-two (46 %) articles proposed mixed methods research, with 60 (54 %) proposing only quantitative methods. Sixty-five (58 %) protocols reported using a model to guide the study. The three most frequently reported models were RE-AIM (n = 4), PARIHS (n = 3), and Diffusion of Innovations (n = 3). Most studies were in Canada (29 %) or the USA (22 %).

Implications for D&I Research: While several novel designs for D&I research have been proposed by leaders in the field (e.g., stepped-wedge, interrupted time-series), the majority of the studies in our sample used RCT designs. Alternative study designs may be underutilized for a variety of reasons. Promisingly, the prevalent use of mixed methods approaches reflects methodological innovation in newer D&I research. Training programs and resources for D&I researchers should consider incorporating additional resources on alternative study designs.

Primary Funding Source: National Institutes of Health - This project was supported by the National Institute of Health (UL1 TR000448) National Institute of Mental Health (T32MH019960, R25 MH080916, and F31MH098478); National Cancer Institute (NCI U54CA155496); National Institute of Diabetes and Digestive and Kidney Diseases.

## Prevention and public health

### S62 PANEL: Geographic variation in the implementation of public health services: Economic, organizational, and network determinants - Model simulation techniques to estimate the cost of implementing foundational public health services

#### Cezar Brian Mamaril^1^, Glen Mays^2^, Keith Branham^1^, Lava Timsina^3^

##### ^1^Health Management and Policy, University of Kentucky, College of Public Health, Lexington, KY, 40536, USA; ^2^Department of Health Services Management, University of Kentucky, College of Public Health, Lexington, KY, 40536, USA; ^3^Systems for Action National Program Office, University of Kentucky, College of Public Health, Lexington, KY, 40536, USA

###### **Correspondence:** Cezar Brian Mamaril – Health Management and Policy, University of Kentucky, College of Public Health, Lexington, KY, 40536, USA

**Background:** A 2012 Institute of Medicine Report on public health finance recommended research to identify the components and costs of a “minimum package of public health services” that should be available in every U.S. community. A national expert panel convened by the Robert Wood Johnson Foundation developed definitions and specifications for this minimum package in 2014, referenced as Foundational Public Health Services. This study uses a novel, simulation-based cost-estimation methodology to estimate current implementation costs and projected resource needs for these services, accounting for inherent variation and uncertainty in resources required for implementing these activities.

**Methods:** A self-administered cost estimation instrument was developed to allow public health agency administrators to estimate labor and non-labor resources used for implementing each service. The instrument was administered to both state and local public health officials in the state of Kentucky. Probability distributions of both input costs and output costs were generated using Latin Hypercube Sampling (LHS) for the 11 service domains. Total per capita costs were estimated for current levels of service and for projected service levels required to fully implement each service as defined by the expert panel. We performed sensitivity analyses to determine which inputs have the largest influence on FPHS costs.

**Findings:** Model simulation results indicate minimum, median, and maximum current per capita costs of implementing foundational public health services were equal to $39, $65, and $90 respectively (coefficient of variation = 12.17). The projected minimum, median, and maximum per capita cost to fully implement services consistent with expert panel recommendations were estimated at $54, $101, and $149 (coefficient of variation = 15.25). The estimated minimum, median, and maximum per capita values of the gap between current and projected costs were $-17, $36, and $ 92 respectively (coefficient of variation = 46.79). Sensitivity analyses suggest that labor costs associated with Maternal, Child and Family health and organizational competencies were the largest drivers of total service costs.

Implications for D&I Research: This study demonstrates the feasibility and value of a hybrid cost-estimation methodology that combines survey-based cost allocation approaches with model simulation techniques to estimate variation in the costs of implementing public health services.

Primary Funding Source: The Robert Wood Johnson Foundation.

### S63 PANEL: Geographic variation in the implementation of public health services: Economic, organizational, and network determinants - Inter-organizational network effects on the implementation of public health services

#### Glen Mays^1^, Rachel Hogg^2^

##### ^1^Department of Health Services Management, University of Kentucky, College of Public Health, Lexington, KY, 40536, USA; ^2^College of Health Sciences, University of Kentucky, Lexington, KY, 40536, USA

###### **Correspondence:** Glen Mays – Department of Health Services Management, University of Kentucky, College of Public Health, Lexington, KY, 40536, USA

**Background:** The Affordable Care Act created new incentives for hospitals, insurers, public health agencies, and others to contribute to disease prevention and health promotion activities, potentially changing inter-organizational relationships and expanding implementation of strategies that improve population health. This study uses data from the 1998-2014 National Longitudinal Survey of Public Health Systems to examine: (1) the extent and nature of change in inter-organizational contributions to public health activities; (2) whether network changes attenuate or exacerbate disparities in public health implementation across communities; and (3) how network changes affect preventable mortality and resource use.

**Methods:** We follow a cohort of more than 350 U.S. metropolitan communities over time using survey data collected initially in 1998 and again in 2006, 2012 and 2014. Local public health officials report on the availability of 20 guideline-recommended public health activities in the community, the organizations that contribute to each activity, and the perceived effectiveness of each activity. We construct network-analytic measures of inter-organizational connectedness in implementing activities (density, degree and betweenness centrality), with a focus on hospitals, public health agencies, and community-based organizations. We link survey data with outcome measures that include county-level cause-specific mortality rates and measures of public health agency expenditures. Fixed-effects models with instrumental-variables are used to estimate changes in preventable mortality and expenditures attributable to changes in network structure, controlling for observable and unmeasured confounders.

**Findings:** During 2012-14, hospitals increased their implementation of public health activities by 20.1 % in the average U.S. community, compared with an increase of 8.7 % by local public health agencies, an increase of 6.3 % by community-based nonprofit organizations, and a reduction of 6.6 % by state agencies. Disparities in implementation between the top and bottom 20 % of U.S. communities increased by 30.2 % during the full 1998-2014 period, with more than two-thirds of this disparity attributable to changes in inter-organizational network structure. Increases network density and centrality were associated with statistically-significant reductions in infant mortality and deaths due to cardiovascular disease, diabetes, and cancer, and also reductions in public health resource use.

Implications for D&I Research: Highly-connected and integrated inter-organizational structures support improved implementation of recommended public health services, improved outcomes, and lower resource use.

Primary Funding Source: The Robert Wood Johnson Foundation.

### S64 PANEL: Building capacity for implementation and dissemination of the communities that care prevention system at scale to promote evidence-based practices in behavioral health - Implementation fidelity, coalition functioning, and community prevention system transformation using communities that care

#### Abigail Fagan^1^, Valerie Shapiro^2^, Eric Brown^3^

##### ^1^Department of Sociology and Criminology & Law, University of Florida, Gainesville, FL, 32611, USA; ^2^School of Social Welfare, UC Berkeley, Berkeley, CA, 94720, USA; ^3^Department of Public Health Sciences, University of Miami, Miami, FL, 33136, USA

###### **Correspondence:** Abigail Fagan – Department of Sociology and Criminology & Law, University of Florida, Gainesville, FL, 32611, USA

**Background:** The success of community-based prevention rests on the ability to effectively mobilize community members to adopt and implement with quality evidence-based interventions. Yet relatively few models have been created, tested, and shown to achieve such outcomes. The Communities That Care (CTC) prevention system is designed to achieve these goals by helping communities form broad-based, diverse coalitions to guide prevention efforts, adopt effective programs and policies, and monitor coalition activities and preventive interventions to ensure coordinated, well-implemented prevention services which are sustained over time.

**Methods:** Results are based on data from a randomized trial of CTC in 24 communities. Implementation of the CTC prevention system in the 12 intervention communities, including completion of required goals and actions, was assessed periodically via completion of the CTC Milestones and Benchmarks survey by local coalition coordinators and research staff. Data on the implementation of prevention programs was provided by agency directors and service providers, school principals, and teachers in all communities. Surveys with 10-15 key leaders in each community assessed collaboration across community sectors and use of a science-based approach to prevention. Interviews with up to 20 coalition members assessed the functioning of prevention coalitions.

**Findings:** Intervention communities achieved and sustained high-fidelity implementation of the CTC system and tested, effective interventions. Compared to control communities, CTC communities implemented and sustained a greater number of preventive interventions and delivered them to a significantly greater number of youths and parents. CTC communities achieved greater collaboration across community sectors and were more likely to adopt a science-based approach to prevention than control communities. Better functioning CTC coalitions were better able to develop members’ prevention skills and establish linkages with other organizations which contributed to the successful adoption of science-based prevention. Level of adoption was related to greater community-level improvements in youth outcomes in CTC compared to control communities.

**Implications for D&I Research:** Achieving high quality implementation and widespread dissemination of tested and effective programs and policies are important goals of dissemination and implementation research. The CTC prevention system has been shown to assist local communities in achieving these goals in a randomized trial.

Primary Funding Source: National Institutes of Health.

### S65 PANEL: Building capacity for implementation and dissemination of the communities that care prevention system at scale to promote evidence-based practices in behavioral health - Expanding capacity for implementation of communities that care at scale using a web-based, video-assisted training system

#### Kevin Haggerty, David Hawkins

##### School of Social Work, University of Washington, Seattle, WA, 98115, USA

###### **Correspondence:** Kevin Haggerty – School of Social Work, University of Washington, Seattle, WA, 98115, USA

**Background:** Communities That Care (CTC) is a proven prevention system ready for broad dissemination. However, translating efficacious approaches into widespread practice in communities remains a challenge. CTC is traditionally implemented in communities by in-person certified trainers through eight community training events that require 9 days of training over the course of 12 to 18 months. The use of in-person trainers who visit communities to conduct training workshops limits the accessibility, flexibility, and scalability of CTC. A cost-effective mechanism to more easily disseminate trainings to communities is needed to facilitate widespread implementation.

**Methods:** The Center for Communities That Care at the University of Washington recently developed a web-based, video-assisted training system (“eCTC”) that addresses this need. The web-streamed CTC system includes 120 3-5 minute videos presenting all CTC training content. New eCTC workshops are led by a local community facilitator and do not require a certified CTC trainer on site.

**Findings:** This presentation will describe the new web-based video training system, share first experiences from using eCTC for training in several communities, and discuss implications for the dissemination of the CTC operating system.

Implications for D&I Research: Current work is the first step toward testing the efficacy of a web-based training. If it is no less efficacious than in-person training, and if the web training is found to be an improvement over current “prevention-as-usual” approaches, then eCTC has the potential to disseminate prevention science knowledge, promote the implementation of effective prevention programs in communities, and achieve improvements in the health and well-being of America’s youth at a broad scale with flexibility and potential cost savings.

Primary Funding Source: National Institutes of Health.

### S66 PANEL: Building capacity for implementation and dissemination of the communities that care prevention system at scale to promote evidence-based practices in behavioral health - Effects of communities that care on reducing youth behavioral health problems

#### Sabrina Oesterle, David Hawkins, Richard Catalano

##### School of Social Work, University of Washington, Seattle, WA, 98115, USA

###### **Correspondence:** Sabrina Oesterle – School of Social Work, University of Washington, Seattle, WA, 98115, USA

**Background:** Communities That Care (CTC) is a planning system that helps communities to prevent youth behavioral health problems. Through the selection and implementation of tested and effective policies and programs that are matched to the community’s needs, CTC aims to reduce elevated risk factors and improve suppressed protective factors, which are expected, in turn, to decrease youth behavioral health problems and improve youth development community-wide. CTC’s capacity to reduce youth violence, delinquency, and substance use was tested in a community-randomized trial of CTC in 24 small towns in 7 states, matched in 12 pairs within state and assigned randomly to the control or CTC condition in 2003.

**Methods:** The trial surveyed annually a panel of 4407 5th-graders attending public schools from 2004 until 2012, 1 year after on-time high school graduation. The survey was completed by at least 91 % of the still-living sample in all years. The sample is gender-balanced and primarily non-Hispanic White (64 %) with 20 % identifying as Hispanic or Latino youth. Data were analyzed using generalized mixed regression models to account for the nesting of the data.

**Findings:** Analyses showed significant reductions in targeted risk factors among youth living in CTC communities compared to youth in control communities and lower incidence and prevalence of adolescent delinquency, violence, alcohol use, and cigarette smoking. Reductions in risk were found as early as 7^th^ grade, 2 years after initial implementation of CTC. Reductions in problem behaviors were found starting in 8^th^ grade, some of which were sustained through high school and age 19, 9 years after initial implementation of CTC and 4 years after training, technical assistance, and financial support to CTC communities had ended.

**Implications for D&I research:** Public health can be promoted and health risk behaviors in early adolescence can be prevented by coalitions of community stakeholders trained to use the CTC system for translating the advances of prevention science into well-chosen and well-implemented prevention practices in communities. To achieve sustained effects, CTC communities may need to expand prevention efforts to include preventive programming targeting high school students as well as younger ages.

**Primary funding source:** National Institutes of Health - R01 DA015183.

### S68 When interventions end: the dynamics of intervention de-adoption and replacement

#### Virginia McKay^1^, M. Margaret Dolcini^2^, Lee Hoffer^3^

##### ^1^College of Public Health and Human Sciences, Oregon State University, Corvallis, OR, 97331, USA; ^2^School of Social and Behavioral Health Sciences, Oregon State University, Corvallis, OR, 97331, USA; ^3^Department of Anthropology, Case Western Reserve University, Cleveland, OH, 44106, USA

###### **Correspondence:** Virginia McKay – College of Public Health and Human Sciences, Oregon State University, Corvallis, OR, 97331, USA

**Background:** Implementation research in public health has emphasized the early stages of evidence-based intervention (EBI) implementation, leaving the impacts of intervention de-adoption largely unexplored. The shift in HIV prevention approaches from the Diffusion of Evidence-based Interventions (DEBIs) to High Impact Prevention (HIP) by the Centers for Disease Control and Prevention provides an opportunity to explore EBI de-adoption and replacement. Using complex adaptive systems (CAS) as a broad framework (i.e., multiple heterogeneous components, interaction, and the environmental context), which suggests EBIs influence the public health system in complex ways over time, the current mixed method study aims to examine the impacts of shifting from a DEBI to a HIP intervention for an HIV prevention agency and the community it serves.

**Methods:** We conducted a case study with a community-based organization dedicated entirely to HIV prevention. This agency implemented RESPECT, a DEBI, for four years (2010-2014), and then de-adopted and replaced RESPECT with ARTAS, a HIP intervention. We collected archival data documenting RESPECT implementation (e.g., agency reports, RESPECT participant records) and two semi-structured interviews with staff involved in RESPECT and ARTAS implementation (N = 5). Data were synthesized to develop a narrative of RESPECT implementation, de-adoption, and replacement with ARTAS and the impacts of these events over time.

**Findings:** In alignment with CAS, RESPECT de-adoption and replacement with ARTAS had wide-reaching influences on the agency and the community, the interaction among individuals, the resources available at the agency. Many resources developed to successfully implement RESPECT (e.g., staff positions, staff skill) were lost or irrelevant for ARTAS. Clients and other staff at the agency continued to request RESPECT, and ARTAS programming overlapped heavily with existing services. Together, these factors generated confused and negative interactions among staff and clients.

**Implications for D&I research:** Although it may be inevitable that existing EBIs are replaced with more efficient or effective EBIs, this early exploratory analysis suggests that EBI de-adoption may have important, unintended consequences (e.g., inefficient use of resources, poor agency interactions with the community). The field would benefit from greater attention to de-adoption in dissemination and implementation theoretical frameworks.

**Primary funding source:** Oregon State University

### S69 Results from next-d: can a disease specific health plan reduce incident diabetes development among a national sample of working-age adults with pre-diabetes?

#### Tannaz Moin^1,2^, Jinnan Li^1^, O. Kenrik Duru^1^, Susan Ettner^1,3^, Norman Turk^1^, Charles Chan^4^, Abigail Keckhafer^5^, Robert Luchs^4^, Sam Ho^6^, Carol Mangione^1^

##### ^1^Division of General Internal Medicine and Health Services Research, David Geffen School of Medicine at University of California, Los Angeles, CA, 90024, USA; ^2^HSR&D Center for the Study of Healthcare Innovation, Implementation, and Policy, VA Greater Los Angeles Healthcare System, Los Angeles, CA, 90073, USA; ^3^Department of Health Policy and Management, Fielding School of Public Health, University of California, Los Angeles, CA, 90095, USA; ^4^Actuarial Pricing, UnitedHealthcare, Minneapolis, MN, 55343, USA; ^5^Actuarial Pricing, UnitedHealthcare, Minneapolis, MN, 55369, USA; ^6^Innovations, UnitedHealthcare, Minneapolis, MN, 55343, USA

###### **Correspondence:** Tannaz Moin – Division of General Internal Medicine and Health Services Research, David Geffen School of Medicine at University of California, Los Angeles, CA, 90024, USA

**Background:** Prediabetes affects one third of adults in the United States and can significantly impact health outcomes and costs for large segments of the population who progress to diabetes. Population-wide approaches to diabetes prevention may include innovative health insurance benefit designs targeting working-age adults with prediabetes. Thus, our objective was to determine whether the Diabetes Health Plan (DHP), the first disease specific health plan designed with incentives to improve care for persons with prediabetes and diabetes, lowers rates of incident diabetes among adults with prediabetes.

**Methods:** Our analysis included data from a large, national private insurer offering health plans to public and private employers between 2009-2013. In this natural experiment, propensity score matching was conducted at the employer-level to find comparable control employer groups, and an adjusted logistic regression model at the individual-level was used to test the association between DHP employer group status and incident diabetes diagnosis during the 3-years of follow-up after baseline. We examined eligibility and claims data from continuously covered employees and dependents insured over a 4-year study window. Our primary outcome was incident diabetes over 3 years of follow-up after baseline.

**Findings:** Our analysis included data from 11,965 continuously enrolled adults with prediabetes (N = 1,538 from 9 employers offering the DHP; N = 10,427 from 105 control employers offering standard plans). DHP employees and covered dependents with prediabetes had a 7.6 % lower absolute predicted probability of incident diabetes compared to individuals from employer groups offering standard benefit plans (29 % predicted probability of incident diabetes for DHP vs. 37 % for controls, p < .001).

**Implications for D&I research:** Our findings indicate that health insurance benefit designs that specifically increase prediabetes awareness and provide incentives and/or reduce barriers to recommended care for persons with prediabetes may be a viable means of preventing or delaying incident diabetes for working-age adults. To our knowledge, this is one of the first studies to examine the impact of health insurance benefit design on outcomes for patients with prediabetes and an area of needed future study.

**Primary funding source:** Centers for Disease Control and Prevention - CDC and NIH/NIDDK.

### S70 Implementing smoking cessation interventions in primary care settings (STOP): using the interactive systems framework

#### Peter Selby^1,2,3^, Laurie Zawertailo^1,4^, Nadia Minian^5^, Dolly Balliunas^6^, Rosa Dragonetti^5^, Sarwar Hussain^6^, Julia Lecce^6^

##### ^1^Addictions Program, Centre for Addiction and Mental Health, Toronto, ON, M5T1P7, Canada; ^2^Family and Community Medicine, University of Toronto, Toronto, ON, M5T1P7, Canada; ^3^Psychiatry, Dalla Lana School of Public Health, University of Toronto, Toronto, ON, M5T1P7, Canada; ^4^Pharmacology and Toxicology, University of Toronto, Toronto, ON, M5T1P7, Canada; ^5^Addictions Medicine, Centre for Addiction and Mental Health, Toronto, ON, M5S 3E3, Canada; ^6^Nicotine Dependence Service, Centre for Addiction and Mental Health, Toronto, ON, M5T 1P7, Canada

###### **Correspondence:** Peter Selby – Addictions Program, Centre for Addiction and Mental Health, Toronto, ON, M5T1P7, Canada

**Background:** Implementing cancer prevention practices in primary care such as smoking cessation is challenging due to system, practitioner and patient factors. The Interactive Systems Framework (ISF) describes the smooth interactions between three systems to move research to practice. These are the Prevention Synthesis and Translation, Prevention Support, and the Prevention Delivery systems. The challenge is communication across these systems to ensure successful implementation and maintenance.

**Methods:** Using the ISF, we synthesized the existing guidelines for smoking cessation (www.canadaptt.net). Then with support from the provincial funder, a variety of primary care settings in Ontario such as Family Health Teams (FHTs), Community Health Centres (CHCs), and Nurse Practitioner-Led Clinics (NPLCs) were invited to complete a capacity assessment to implement a smoking cessation program for smokers motivated to quit with access to counselling and up to 26 weeks of nicotine replacement therapy. Once assessments were completed, organizations were clustered according to implementation readiness. We created tailored implementation plans, trainings and technical support codified in bidirectional binding agreements that allowed rapid implementation and immediate impact on practice. Ongoing coaching and communication by phone and the internet on a biweekly basis allows the central team to manage over 300 sites. The intervention and data are collected via a web-based portal with individual performance feedback to practices to engage them in quality improvement. Patient level outcomes are collected at 3, 6 and 12 months and used to design further interventions for other risk behaviours.

**Findings:** Over the last 4 years, Smoking Treatment for Ontario Patients (STOP) is implemented at 84 % FHTs, 90 % CHCs and 73 % NPLCs. Over 1,000 practitioners have received operations and technical training. Enrollment into STOP has exceeded 56,000 patients. Smoking cessation rates at 6 month follow-up are FHTs–36.9 %, CHCs–28.7 %, and NPLCs–31.1 %. Practice drop out is < 2 %.

**Implications for D&I research:** We have demonstrated the utility of the ISF model with indicators at the system and patient level. We also demonstrate that it is feasible and desirable for various systems to collaborate to ensure scale up of prevention interventions. Outcomes can be reported using the RE-AIM framework.

**Primary funding source:** Ontario Ministry of Health & Long-Term Care

### S71 Testing the Getting To Outcomes implementation support intervention in prevention-oriented, community-based settings

#### Matthew Chinman^1,2^, Joie Acosta^3^, Patricia Ebener^4^, Patrick S Malone^1^, Mary Slaughter^1^

##### ^1^Health, RAND Corporation, Pittsburgh, PA, 15213, USA; ^2^VISN 4 MIRECC, VA Pittsburgh Healthcare Center, Pittsburgh, PA, 15206, USA; ^3^Health, RAND Corporation, Arlington, VA, 22202-5050, USA; ^4^Health, RAND Corporation, Santa Monica, CA, 90407, USA

###### **Correspondence:** Matthew Chinman – Health, RAND Corporation, Pittsburgh, PA, 15213, USA

**Background:** Implementation research is sparse, yet sorely needed, in prevention-oriented, community-based settings, which often have limited resources that can undermine implementation quality and outcomes.

**Methods:** This presentation describes a Hybrid Type II, cluster-randomized controlled trial comparing two conditions: (1) 16 Boys & Girls Club (BGC) sites implementing an evidence-based, teen pregnancy prevention called Making Proud Choices (MPC) for two years; (2) 16 similar BGC sites implementing MPC augmented with a two-year implementation support intervention called Getting To Outcomes (MPC + GTO). All sites received training and manuals typical for MPC. GTO consists of its own manuals, training, and onsite technical assistance to help practitioners complete key programming tasks specified by the GTO 10 Step model. During the first year, TA providers helped MPC + GTO sites adopt, plan, and deliver MPC. Sites then received training on the evaluation and quality improvement steps of GTO, along with feedback reports summarizing their data, which were used in a TA-facilitated quality improvement process that yielded a revised plan for the second MPC implementation. The trial assessed whether GTO improves performance of key programming tasks (e.g., goal setting, planning, evaluation, quality improvement), fidelity to MPC, and youth sexual health outcomes (knowledge, attitudes, and behaviors around condoms and sex). Performance was measured using ratings made from a standardized, structured interview with participating staff at all 32 BGC sites after the first and second years of MPC implementation. Multiple elements of fidelity (adherence, classroom delivery, dosage) were assessed at all sites by observer ratings and attendance logs. Youth sexual health outcomes were assessed via surveys before, immediately following, and 6-months after MPC.

**Findings:** After the second year, MPC + GTO sites had significantly higher ratings of performance, classroom delivery, and adherence (e.g., 92 % vs. 55 % MPC activities fully implemented). Dosage remained similar. Youth in MPC + GTO sites showed improvement in more sexual health outcomes than MPC only youth.

**Implications for D&I research:** This study is the first that assesses an implementation support intervention’s impact on performance, implementation quality, and individual outcomes simultaneously and similarly in both study conditions. The findings suggest that GTO’s implementation support can help community-based settings achieve high levels of fidelity and outcomes.

**Primary funding source:** National Institutes of Health - A grant from National Institute on Child Health & Human Development (1R01HD069427-01).

### S72 Examining the reach of a multi-component farmers’ market implementation approach among low-income consumers in an urban context

#### Darcy Freedman^1,2^, Susan Flocke^1,3^, Eunlye Lee^4^, Kristen Matlack^2^, Erika Trapl^2^, Punam Ohri-Vachaspati^5^, Morgan Taggart^6^, Elaine Borawski^2^

##### ^1^Epidemiology and Biostatistics, Case Western Reserve University, Cleveland, OH, 44106, USA; ^2^Prevention Research Center for Healthy Neighborhoods, Case Western Reserve University, Cleveland, OH, 44106, USA; ^3^Family Medicine, Case Western Reserve University, Cleveland, OH, 44106, USA; ^4^Mandel School of Applied Social Sciences, Case Western Reserve University, Cleveland, OH, 44106, USA; ^5^School of Nutrition and Health Promotion, Arizona State University, Phoenix, AZ, 85004, USA; ^6^Ag|re|culture, St. Clair Superior Development Corporation, Cleveland, OH, 44103, USA

###### **Correspondence:** Darcy Freedman – Epidemiology and Biostatistics, Case Western Reserve University, Cleveland, OH, 44106, USA

**Background:** Implementation strategies designed to increase the reach of Farmers’ markets (FMs) among people receiving Supplemental Nutrition Assistance Program (SNAP) benefits include locating FM in low-income neighborhoods, accepting SNAP benefits at FM, and operating FM healthy food incentive programs. All of these strategies are being implemented at FMs throughout Cleveland, Ohio. We examine FM shopping frequency and FM awareness, social norms, and beliefs among SNAP recipients living within one mile of a FM.

**Methods:** Geographic-based sampling focused on areas in Cleveland within one mile of a FM including Census tracts with a SNAP participation rate of >30 % resulting in 16 FM centroids. Next, using community-based recruitment, adult SNAP recipients with children were invited to complete a cross-sectional survey. Data were collected from June-August 2015. Descriptive statistics, chi square and ANOVA were used to test the association of FM shopping frequency with social norms and beliefs.

**Findings:** A total of 238 SNAP recipients participated, most identified as African American (85.3 %), female (87.8 %) with household income of < $10,000 (66.7 %) and mean age of 38.6 years. FM utilization ranged from never (33.0 %); ever but not in last year (24.5 %); and within the last year, 1-2 times (15.9 %), 3-6 times (15.9 %), and 7+ (10.7 %). Greater frequency of FM utilization was associated with increased awareness of FM in their neighborhood (p < .001), awareness of the FM healthy food incentive program (p < .005), and use of SNAP benefits to purchase fruits and vegetables at FMs (p < .002). However, overall use of the incentive program was low (17.7 %). Those who had shopped at a FM in the past year were significantly more likely to report stronger social norms for FM use, greater frequency of being invited to a FM, and had more positive beliefs about FMs.

**Implications for D&I research:** FM use among the sample was relatively high, yet many were not aware of FMs in their neighborhood or healthy food incentive programming. Findings support dissemination research to increase awareness of the multicomponent FM intervention approach including social network-based diffusion. Given low levels of awareness and use of the incentive program, targeted research is needed to better understand roadblocks to implementation and uptake.

**Primary funding source:** Centers for Disease Control and Prevention - Prevention Research Center core research, grant number: 1U48DP005030-01.

### S73 Increasing implementation of evidence-based health promotion practices at large workplaces: The CEOs Challenge

#### Amanda Parrish^1^, Jeffrey Harris^2^, Marlana Kohn^1^, Kristen Hammerback^1^, Becca McMillan^3^, Peggy Hannon^4^

##### ^1^Department of Health Services, University of Washington, Health Promotion Research Center, Seattle, WA, 98105, USA; ^2^Health Promotion Research Center, University of Washington, Seattle, WA, 98105, USA; ^3^Partner Relationships, American Cancer Society, Seattle, WA, 98109, USA;^4^Health Services, University of Washington, Seattle, WA, 98105, USA

###### **Correspondence:** Amanda Parrish – Department of Health Services, University of Washington, Health Promotion Research Center, Seattle, WA, 98105, USA

**Background:** Implementing health-related evidence-based practices (EBPs) in the workplace can increase positive health behaviors like cancer screening, healthy eating, physical activity, and tobacco cessation. But EBPs are under-used, even in large workplaces. We developed and tested The CEOs Challenge to support large companies to implement and maintain health-promoting EBPs focused on programs, policy/environment, and communications.

**Methods:** We tested The CEOs Challenge with companies engaged in the Washington State chapter of the American Cancer Society’s “CEOs Against Cancer” network of local organizations. At kickoff of The CEOs Challenge, we assessed company implementation of 17 health-related EBPs via questionnaire and scored them on a scale from 0 to 100. Participating companies designated an Executive and an operations-level Manager to run the program; both received a written report of company baseline performance and aggregate chapter data for comparison with peer companies. Over the following year company Managers engaged in at-least-quarterly consultations with American Cancer Society staff trained to assist implementation of EBPs; they selected EBP focus areas and received customized tools and support. Company Executives gathered at thrice-yearly chapter meetings and discussed progress and barriers/facilitators. Follow-up performance was measured at 1 year. Companies received follow-up reports summarizing company progress, and aggregate chapter progress.

**Findings:** Seventeen companies participated in The CEOs Challenge. At baseline the mean implementation scores were 46 for healthy eating, 55 for cancer screening, 60 for physical activity, and 68 for tobacco cessation. One year later, these scores increased by 20, 19, 16, and 9 points respectively. Companies reported positive experiences with the program, valuing customized support and data tracking company and chapter progress.

**Implications for D&I research:** The CEOs Challenge is a promising approach to sustainable chronic disease prevention via the workplace. The program increased workplace adoption of EBPs after 1 year, and company engagement continues. This model could be implemented in other CEOs Against Cancer chapters across the US, with the potential to significantly improve evidence-based health promotion opportunities for millions of US employees. Current steps include developing and testing tools to ensure that low-SES employees are aware of and have access to the newly-implemented EBPs, and studying whether employers continue to sustain implementation of EBPs.

**Primary funding source:** American Cancer Society.

### S74 A qualitative assessment of barriers to nutrition promotion and obesity prevention in childcare

#### Taren Swindle^1^, Geoffrey Curran^2^, Leanne Whiteside-Mansell^1^, Wendy Ward^3^

##### ^1^Family and Preventive Medicine, University of Arkansas for Medical Sciences, Little Rock, AR, 72205, USA; ^2^Department of Pharmacy Practice, University of Arkansas for Medical Sciences, Little Rock, AR, 72205, USA; ^3^Pediatrics, University of Arkansas for Medical Sciences, Little Rock, AR, 72205, USA

###### **Correspondence:** Taren Swindle – Family and Preventive Medicine, Univesity of Arkansas for Medical Sciences, Little Rock, AR, 72205, USA

**Background:** Given that 59.3 % of low-income children between ages 3 and 4 are in childcare more than 30 hours per week, this setting provides a promising context for addressing and reducing the disparity in childhood obesity rates. However, observational studies and policy reviews suggest that childcare programs are not consistently adopting evidence-based practices in obesity prevention and nutrition promotion. For example, educators often pressure children to eat and fail to signal hunger cues. The individual (i.e. early childhood educator, ECE) and organizational (i.e., center, agency) factors that act as barriers to use of best practice are largely unexplored.

**Methods:** Qualitative interviews with 29 ECEs were conducted to examine perceptions of their role in child nutrition and barriers to positive mealtime practices. Belsky’s model of parenting was adapted to inform the initial interview guide that explored ECEs’ personal backgrounds, beliefs, and work context in the arena of child feeding and nutrition. Stakeholders provided input to refine the interview guide, and four pilot interviews were conducted to finalize the question content. Directed content analysis was employed to code interviews using theoretical constructs as sensitizing concepts. An abstract of each interview was created to assess individual fit with the theoretical model. Thereafter, coding across cases was completed to identify common themes. The principal investigator and one research assistant completed coding after reaching 85 % agreement on a random sample of 5 interviews.

**Findings:** A history of personal food insecurity, lack of supporting policy, and belief that nutrition is a parent's job were recurring themes. Themes from this study highlight how characteristics of individuals and perceived characteristics of their organizations may contribute to a failure to implement evidence-based practices, adhere to existing policies, and de-implement counterproductive behaviors. Findings illustrate that educator practices of educators are entrenched in their own backgrounds and beliefs and interact with their perceptions of the organizational context.

**Implications for D&I research:** The current study illustrates that barriers can be very context-specific. Given the inconsistent use of a driving theory in the field of implementation science, this study also illustrates how a theoretical framework can be used to provide a guide for exploration of barriers in a given context.

**Primary funding source:** National Institutes of Health.

## Promoting health equity and eliminating disparities

### S75 Documenting institutionalization of a health communication intervention in African American churches

#### Cheryl Holt^1^, Sheri Lou Santos^1^, Erin Tagai^1^, Mary Ann Scheirer^2^, Roxanne Carter^3^, Janice Bowie^4^, Muhiuddin Haider^5^, Jimmie Slade^3^, Min Qi Wang^1^

##### ^1^Behavioral and Community Health, University of Maryland, College Park, MD, 20742, USA; ^2^Program Evaluation, Scheirer Consulting, Princeton, NJ, 8540, USA; ^3^Community Ministry of Prince George's County, Upper Marlboro, MD, 20772, USA; ^4^Health Behavior and Society, Johns Hopkins University, Baltimore, MD, 21205, USA; ^5^Maryland Institute for Applied Environmental Health, University of Maryland, College Park, MD, 20742, USA

###### **Correspondence:** Cheryl Holt – Behavioral and Community Health, University of Maryland, College Park, MD, 20742, USA

**Background:** Institutionalization is defined as the extent to which an evidence-based intervention is integrated into the culture of the recipient setting or community through policies or practice. Research on successful institutionalization has to date been limited.

**Methods:** We examined the naturally occurring (e.g., unprompted) institutionalization of health promotion activities following a cancer educational workshop series delivered through trained and certified lay peer community health advisors (CHAs) in 14 African American churches. Twenty three CHAs completed interviews with project staff at 12 months follow-up. They reported on institutionalization of health promotion activities in their churches as expressed through a variety of indicators such as health policy implementation, health ministry development, subsequent CHA training in health promotion, and church leadership support for health promotion.

**Findings:** Evidence of institutionalization was provided though CHA reports of these indicators of institutionalization. One quarter (24 %) of the CHAs reported that their churches had formed a new health ministry, one CHA reported adoption of a new health policy, while 48 % said that their church updated its church health policy (e.g., healthier meals, no smoking). Many CHAs reported receiving additional training in cancer (55 %) or a different health topic (65 %). Many reported pastoral support of health promotion through sermons (70 %), announcements (87 %), church bulletins (83 %) that included health content, inviting church members to attend the health workshops (91 %), or attending the workshops themselves (65 %).

**Implications for D&I research:** We documented evidence of naturally occurring institutionalization of health promotion activities following a cancer education intervention trial conducted by lay CHAs. Institutionalization is an important vehicle for sustainability and should be considered in a purposive manner when developing interventions for implementation in organizations. This may also involve targeted capacity building efforts.

**Primary funding source:** National Institutes of Health - This research is funded by the National Cancer Institute (#R01CA147313). None of the authors have any commercial interests.

### S76 Reduction in hospital utilization by underserved patients through use of a community-medical home

#### Andrew Masica, Gerald Ogola, Candice Berryman, Kathleen Richter

##### Center for Clinical Effectiveness, Baylor Scott & White Health, Dallas, TX, 75206, USA

###### **Correspondence:** Andrew Masica – Center for Clinical Effectiveness, Baylor Scott & White Health, Dallas, TX, 75206, USA

**Background:** Our objective was to evaluate the impacts of a community-based medical home (CMH) program on hospital utilization following index admissions in an underserved population.

**Methods:** We applied two study designs simultaneously: prospective observational (to evaluate the effects of receiving care at the CMH), and for the patient subgroup establishing CMH follow-up, a randomized, controlled component (to assess for incremental benefit from engagement with a “care navigator [CN]” embedded in the CMH). From December 2012 through December 2013, hospitalized patients eligible (income of < 200 % poverty level, lack of insurance, no primary care physician, and ≥ 1 chronic disease) for care at the CMH were scheduled for post-discharge follow-up there. Patients successfully “connected” to the CMH (i.e. keeping their appointment) were randomized 1:3 to receive usual CMH care plus the supplemental CN intervention, or usual CMH care. CN support was administered for a 90-day period and focused on addressing patient-specific social needs (e.g., transportation, prescription assistance). Risk-adjusted hospital utilization was compared among the 3 groups (CN, usual CMH care, non-randomized) over a 1-year period following the index admission.

**Findings:** 418 patients were referred to the CMH; 341 (82 %) patients kept their post-discharge appointments and established CMH follow-up. Eighty-six (25 %) of these connected patients were randomized to the CN. Establishing care at the CMH was associated with significant reductions in hospitalizations vs. patients referred to, but “unconnected” to the CMH over a 1-year period (RR: 0.79, 0.65-0.96). In the group establishing care at the CMH, hospital utilization was decreased in the in patients receiving the CN intervention compared to usual CMH care at the 90-day time point, concurrent with the duration of the support (RR: 0.59, 0.36-0.94); this impact dissipated at 1-year (RR: 90; 73-1.12).

**Implications for D&I research**: In an underserved population with index hospital admissions, patients connected to a CMH had lower rates of subsequent hospital utilization over 1-year compared to patients who did not establish care at the CMH. Use of a CN within the CMH population had additional benefit in reducing hospital utilization, but only while the intervention was active. These models appear to hold promise as effective community-based interventions in high-risk patient groups.

**Primary funding source**: Baylor Irving Foundation.

### S77 Sustainability of evidence-based lay health advisor programs in African American communities: A mixed methods investigation of the National Witness Project

#### Rachel Shelton^1^, Lina Jandorf^2^, Deborah Erwin^3^

##### ^1^Department of Sociomedical Sciences, Columbia University Mailman School of Public Health, New York, NY, 10032, USA; ^2^Oncological Sciences, Mount Sinai School of Medicine, New York, NY, 10029, USA; ^3^Division of Cancer Prevention and Population Sciences, Roswell Park Cancer Institute, Buffalo, NY, 14263, USA

###### **Correspondence:** Rachel Shelton – Department of Sociomedical Sciences, Columbia University Mailman School of Public Health, New York, NY, 10032, USA

**Background:** Lay Health Advisor (LHA) programs hold tremendous promise for reducing health disparities. The National Witness Project (NWP) is one example of an evidence-based LHA program that effectively increases breast and cervical cancer screening among underserved African American women. Over the past twenty years, the program has been successfully disseminated, replicated, and implemented nationally in over 40 sites in 22 states, with over 400 volunteers, reaching over 10,000 women annually.

**Methods:** A longitudinal mixed-methods study (in-depth interviews and surveys) was conducted among 76 LHAs at 8 NWP sites across the northeast, midwest, and southeast between 2010 and 2013. The goal of this study was to better understand individual, social, and organizational factors that influence the motivation, involvement, and retention of LHAs, as well as factors that influence program sustainability overall.

**Findings:** Among LHAs, self-efficacy and role expectations were important predictors of continued involvement in the program a year later. The strongest predictor of having continued implementation and sustainability of LHAs a year after baseline was whether or not their site had a partnership with academic partners. LHAs at sites with academic partnerships were 80 % more likely to still be active at follow-up than LHAs at sites with no academic partnership. There was high variability in program implementation and sustainability, with some programs having no programs in a given year and other programs having as many as 35. Furthermore, there was high turnover of LHAs, with nearly 40 % of LHAs not being active at a year follow-up.

Qualitative data is used to help explain the quantitative findings and provide insight into factors related to organizational context that impact continued program implementation, adaptation, and sustainability.

**Implications for D&I research:** This research contributes to the limited research that has empirically tested factors that impact sustainability of interventions that address health disparities in community settings. In the context of dissemination and implementation science, findings can be used to inform theoretical frameworks and practical strategies focused on the sustainability of evidence-based interventions.

**Primary funding source:** National Institutes of Health - R03 study funded by The National Cancer Institute.

### S78 Predicting the long-term uninsured population and analyzing their gaps in physical access to healthcare in South Carolina

#### Khoa Truong

##### Public Health Sciences, Clemson University, Clemson, SC, 29634, USA

Background: South Carolina (SC) opted not to expand Medicaid under the Patient Protection and Affordable Care Act. The long-term uninsured (LTU) is a core portion of the uninsured population. We aim to predict areas with high concentration of the LTU and analyze LTU’s physical access to healthcare in terms of providers’ quantity, types, and location in SC.

**Methods:** The geographical unit of analysis is Zip Code Tabulation Area (ZCTA). Socioeconomic and demographic data are extracted from the American Community Survey. A statistical model is built to predict the LTU with 16 independent aggregate variables (age, gender, race, education, family status, employment, and poverty). Propensity scores are computed to categorize 424 ZCTAs into the lowest (Q1) and highest (Q5) quintiles of the LTU concentration. Socioeconomic characteristics are then compared across the quintiles. Care provider types, including free health clinics, federally qualified health centers, rural health clinics, and Welvista clinics, typically utilized by uninsured individuals are geocoded. Types of clinics and their availabilities are compared. A set of color-coded maps are created to provide geovisualization of analytical results.

**Findings:** First, ZCTAs with highest LTU concentration, on average have higher rates of minorities, lower socioeconomic status, lower education level, and higher unemployment than ZCTAs with less LTU concentration. Second, there is a significant number of ZCTAs that have high LTU concentration but do not have high Medicaid coverage. Third, about half of ZCTAs in each of quintiles Q3-Q5 do not have any health clinic within their boundaries. Fourth, free health clinics - the type of health care providers that is most likely to be used by the uninsured population is least available in Q5. Last but not least, the average number of clinics per 100,000 population is lowest in Q5.

**Implications for D&I research:** The LTU lack not only health insurance but also physical access to care. Free clinics are effective in reaching out to these populations but their scope is limited because professional services are provided almost entirely by volunteers and local hospitals. Expansion of health insurance does not automatically solve healthcare needs for the uninsured unless health care resources are more available in the surrounding areas.

**Primary funding source:** BlueCross BlueShield of South Carolina Foundation.

### S79 Using an evidence-based parenting intervention in churches to prevent behavioral problems among Filipino youth: A randomized pilot study

#### Joyce R. Javier^1^, Dean Coffey^1^, Sheree M. Schrager^2^, Lawrence Palinkas^3^, Jeanne Miranda^4^

##### ^1^Department of Pediatrics, Division of General Pediatrics, Chidren's Hospital Los Angeles/USC Keck School of Medicine, Los Angeles, CA, 90027, USA; ^2^Division of Hospital Medicine, Chidren's Hospital Los Angeles, Los Angeles, CA, 90027, USA; ^3^Social Work, University of Southern California, Los Angeles, CA, 90089-0411, USA; ^4^Semel Institute for Neuroscience and Human Behavior, UCLA Center for Health Services and Society, Los Angeles, CA, 90024, USA

###### **Correspondence:** Joyce R. Javier – Department of Pediatrics, Division of General Pediatrics, Chidren's Hospital Los Angeles/USC Keck School of Medicine, Los Angeles, CA, 90027, USA

**Background:** Filipino youth have significant behavioral health disparities compared to non-Hispanic whites and other Asian subgroups. Delivering evidence-based parenting interventions in faith settings could be an effective approach to engaging Filipino parents in behavioral health promotion because of their strong affiliation with religious institutions. The purpose of this study was to test an evidence-based parenting program offered in churches and estimate effect size for a fully powered trial.

**Methods:** Twenty-eight Filipino parents of children ages 6-12 years were enrolled in a pilot randomized trial in 2010-2011. They were randomly assigned to either an intervention (i.e., The Incredible Years School Age Basic Parent Program) or a waiting-list control group. Child behavior, parenting practices, and parenting stress were obtained at baseline. Parents in the experimental group attended a series of 12 weekly 2-hour sessions. A follow up assessment was performed after the intervention and 12 weeks later. The study was subsequently replicated with the control group. Satisfaction was assessed after completion of the program with a 40-item measure. ANCOVA was used to compare the intervention group post-intervention versus the control group. Paired t-tests compared mean parenting practices, parenting stress, and child behavior outcomes. Satisfaction was assessed descriptively.

**Findings:** Twenty-two parents (78 %) completed all assessments and the intervention. After completing the program, results showed a significant decrease in physical punishment and parenting stress when the experimental group was compared with the control group. Analyses of all participants comparing pre- and post-intervention revealed parents reported improvements in positive verbal discipline, reductions in child externalizing behaviors, total behavioral problems, parenting stress, and use of physical punishment following the parenting program. Families reported high satisfaction with the content and format of the intervention (means ranged from 5.73 to 6.95 out of 7).

**Implications for D&I research:** Results support the benefits and feasibility of providing an evidence-based parenting program to Filipino parents of school-age children in faith-based settings in order to prevent future behavioral health problems.

**Primary funding source:** National Institutes of Health - Funded by SC CTSI (NIH/NCRR/NCATS) Grant # KL2TR000131.

### S80 Sustainability of elementary school-based health centers in three health-disparate southern communities

#### Veda Johnson^1,2^, Valerie Hutcherson^3^, Ruth Ellis^4^

##### ^1^Pediatrics, Emory University, Atlanta, GA, 30303, USA; ^2^Pediatrics, PARTNERS for Equity in Child and Adolescent Health, Atlanta, GA, 30303, USA; ^3^Johns Creek, GA, 30022, USA; ^4^Pediatrics, Emory University, Atlanta, GA, 30303, USA

###### **Correspondence:** Veda Johnson – Pediatrics, Emory University, Atlanta, GA, 30303, USA

**Background:** In both the South and West, most public school students are from low-income families. Public schools present an opportunity to reach underserved populations on-site with quality healthcare services, yet less than 1 % of public elementary schools house school-based health centers (SBHCs) despite strong evidence of effectiveness from model programs. Four issues must be addressed in taking SBHCs to scale: evidence of community need and support, sustainability, evidence of health and health cost impact, and fidelity to exemplar models. We report on the test of a strategy to solve the first two issues.

**Methods:** Following an exemplar model (Whitefoord Elementary SBHC), the Georgia SBHC Project was created to expand SBHCs in Georgia with 3 phases – planning, implementation, and sustainability. One-year planning grants were awarded in 2010 to Georgia counties for local stakeholders to provide evidence of community need and support. In 2012, 3 counties with widely different racial/ethnic demographics received implementation grants administered by local federally qualified health centers (FQHCs) serving as health center sponsor. PARTNERS for Equity in Child and Adolescent Health at Emory University provided project oversight and technical assistance through workshops, webinars, and monthly conference calls. The SBHCs received an additional year of funding in 2014 to support sustainability efforts, including promoting strong partnerships, conducting clinic outreach and marketing, and establishing quality benchmarks and a strong business model. Evaluation included degree of sustainability and impact on enrollees.

**Findings:** SBHCs recruited and enrolled over 80 % of the student body into the respective centers and increased access to healthcare as demonstrated by increased overall patient encounters, increased encounters for health maintenance with 100 % of students receiving psychosocial assessments, and increased seat time for students accessing the SBHC at one site. Every site advanced toward sustainability, with one site becoming fully sustainable through patient revenue after 2 years of operation.

**Implications for D&I research:** These clinics solved two of the four issues needed to take SBHCs to scale. Evaluation is ongoing regarding health and health cost impact on the state’s Medicaid system and fidelity to exemplar SBHC models following the Implementation Fidelity model, funded by RO1 MD008966-01.

**Primary funding source:** Healthcare Georgia Foundation.

### S81 Childhood obesity prevention partnership in Louisville: creative opportunities to engage families in a multifaceted approach to obesity prevention

#### Anna Kharmats^1,2^, Sandra Marshall-King^3^, Monica LaPradd^3^, Fannie Fonseca-Becker^4^

##### ^1^International Health, Johns Hopkins Bloomberg School of Public Health, Baltimore, MD, 21205, USA; ^2^International Health, Global Obesity Prevention Center, Baltimore, MD, 21205, USA; ^3^Shawnee Christian Health Care Center, Louisville, KY, 40212, USA; ^4^Health, Behavior, & Society, Johns Hopkins Bloomberg School of Public Health, Baltimore, MD, 21202, USA

###### **Correspondence:** Anna Kharmats – International Health, Johns Hopkins Bloomberg School of Public Health, Baltimore, MD, 21205, USA

**Background:** To facilitate neighborhood transformation in a low-income, predominantly African-American community that was facing a substantial burden from obesity-related health disparities, a Federally Qualified Health Center (FQHC) developed the Reaching, Engaging and Promoting Healthy Lifestyles Program (REAP). It established partnerships with Louisville Health Department, the Johnson & Johnson Community Health Care Program, the Johns Hopkins Bloomberg School of Public Health (JHSPH), and with local experts and community organizations.

**Methods:** The REAP intervention engaged children ages 8 to 12 and their caregivers in weekly 2 to 3 hour sessions (for a total of 6 to 8 weeks). Each session included fitness, cooking, nutritional education, and health equity activities. Families also received fresh fruits and vegetables to take home each week. Pre and post-intervention questionnaires and anthropometric measurements were collected. JHSPH provided technical assistance to the FQHC’s in order to build program monitoring and evaluation capacity.

**Findings:** A total of 53 children (40 % boys) and 30 caregivers participated in one of the REAP implementation rounds. At pre-test, 64 % children were overweight or obese, less than 22 % met the recommendations for daily fruit and vegetable intake, 63 % consumed one or less sugar sweetened beverages per day, and only 42 % engaged in at least 60 minutes of daily physical activity. The REAP team encountered substantial challenges with participant retention. In order to accommodate participants’ schedules, session duration and the total number of sessions was reduced. In addition, new partnerships were formed with community organizations. Initial panel logistic and linear regression results suggest that participants who completed the program (n = 32) increased their fruits and vegetables intake by 1.3 servings per day (p = 0.046), and decreased consumption of sugar-sweetened beverages by 0.75 servings per day (p = 0.17).

Implications for D&I research: Preliminary results suggest that the REAP program might be associated with improvements in nutritional intake among low-income African American children. Additionally, key partnerships between the Louisville Health Department, community organizations, and academia were formed and enhanced program sustainability. The FQHC is already utilizing the partnerships formed through the REAP program and applying newly learned program monitoring and evaluation skills to their other initiatives.

**Primary funding source:** Johnson and Johnson Community Healthcare and Scholars Program.

### S82 Improvements in cervical cancer prevention found after implementation of evidence-based Latina prevention care management program

#### Deanna Kepka^1^, Julia Bodson^2^, Echo Warner^2^, Brynn Fowler^2^

##### ^1^Huntsman Cancer Institute, University of Utah, College of Nursing and Huntsman Cancer Institute, Salt Lake City, UT, 84112, USA; ^2^Cancer Control and Population Sciences, Huntsman Cancer Institute & University of Utah, Salt Lake City, UT, 84112, USA

###### **Correspondence:** Deanna Kepka – Huntsman Cancer Institute, University of Utah, College of Nursing and Huntsman Cancer Institute, Salt Lake City, UT, 84112, USA

**Background:** Latinas have the highest incidence of cervical cancer compared to other racial/ethnic groups. Latinas in Salt Lake City who are overdue for recommended cancer screenings are not receiving adequate cervical cancer prevention education and help with access to screening. We assessed correlates of Latina women’s Human Papillomavirus (HPV) related knowledge among a sample of Latina women who were overdue for breast, cervical, and/or colorectal cancer screening and assessed improvements in cervical cancer prevention following the implementation of an evidence-based intervention.

**Methods:** This study occurred in July 2013-June 2014 with N = 211 Latina women recruited by health educators from Latino community based organizations in Utah. Together, university and community organizations adapted the National Cancer Institute’s Prevention Care Management Program to improve cancer prevention and screening among Latinas. Participants completed a 43-item self-reported Spanish-language questionnaire to assess screening history, HPV vaccination knowledge, and demographic characteristics. Descriptive statistics and Fisher’s exact tests were conducted in R to assess correlates of HPV vaccination knowledge. McNemar’s Chi-Squared Test for Count Data was also used to assess the difference between pre- and post-intervention Pap test screening status and certain HPV-related knowledge.

**Findings:** Mean age of participants was 47.1 years (range: 21-70, SD: 11.3 years); most participants were uninsured (89.6 %). Approximately 70 % of study participants were overdue for cervical cancer screening (N = 148). Those who were unemployed were less likely to know that HPV causes cervical cancer (p < .05), that most people have HPV at some point (p < .01), and that the HPV vaccine is more than one dose (p < .01). There was a significant difference between pre- and post-intervention Pap test screening status, with 44.8 % (n = 78) of participants up-to date after the intervention compared to 21.8 % (n = 38) before the intervention (p < 0.01). Certain HPV vaccine related knowledge improved at post-intervention as well. Significantly more participants knew that HPV is able to cause cervical cancer (n = 116, 64.8 % vs. n = 65, 36.3 %; p < 0.01) and that most people have HPV at some point in their lives (n = 112, 62.6 % vs. n = 62, 34.6 %; p < 0.01).

**Implications for D&I research:** The implementation of an evidence-based intervention as a university-community partnership is feasible and can improve cervical cancer prevention practices and knowledge among Utah’s growing Latina population.

**Primary funding source:** University of Utah Internal Grant Award.

### S83 The OneFlorida data trust: Achieving health equity through research & training capacity building

#### Elizabeth Shenkman^1^, William Hogan^1^, Folakami Odedina^2^, Jessica De Leon^3^, Monica Hooper^4^, Olveen Carrasquillo^5^, Renee Reams^6^, Myra Hurt^7^, Steven Smith^8^, Jose Szapocznik^9^, David Nelson^10^, Prabir Mandal^11^

##### ^1^Department of Health Outcomes and Policy, University of Florida College of Medicine, Gainesville, FL, 32606, USA; ^2^Department of Pharmaceutical Outcomes and Policy, University of Florida, Gainesville, FL, 32606, USA; ^3^ Clinical and Translational Science Institute, Florida State University College of Medicine, Tallahassee, FL, 32308, USA; ^4^Psychology, University of Miami, Miami, FL, 33136, USA; ^5^Department of Medicine, University of Miami, Miami, FL, 33136, USA; ^6^Department of Pharmaceutical Sciences, Florida A & M University, Tallahassee, FL, 32308, USA; ^7^College of Medicine, Florida State University, Tallahassee, FL, 32308, USA; ^8^Translational Research Institute, Florida Hospital, Orlando, FL, 32804, USA; ^9^Clinical and Translational Science Institute, University of Miami, Miami, FL, 33136, USA; ^10^Clinical and Translational Science Institute, University of Florida, Gainesville, FL, 32606, USA; ^11^Biology, Edward Waters College, Jacksonville, FL, 32309, USA

###### **Correspondence:** Elizabeth Shenkman – Department of Health Outcomes and Policy, University of Florida College of Medicine, Gainesville, FL, 32606, USA

**Background:** To address health disparities, the IOM recommends focusing on social determinants of health to provide better patient care and enable more informative research. Specifically, sociodemographic and neighborhood variables linked to electronic health record data are needed to better understand the onset and progression of disease, particularly for racial and ethnic minorities. In addition, it is important to increase the representation of racial and ethnic minorities in biomedical research workforce who are trained to use such data in disparities research.

**Methods:** OneFlorida was formed in 2009 to create an infrastructure for implementation science research and pragmatic clinical trials, with a particular emphasis on reducing health disparities in the areas of hypertension, obesity, and tobacco-related cardiovascular diseases and cancer. It is a clinical research consortium of patients, community clinicians, academic health centers, Historically Black Colleges and Universities (HBCUs), health systems, state agencies, and two Clinical and Translational Science Institutes. Its coverage area includes 10 M patients, 1240 physician practices, 4000 physicians, and 22 hospitals. In 2015 it became a member of PCORnet, a national network for conducting clinical comparative effectiveness and patient-centered research.

**Findings:** A key component of OneFlorida is its Data Trust, which integrates Medicaid health care claims, EHR, sociodemographic, and neighborhood data as part of its infrastructure. Currently data are linked for 640,000 enrollees. The claims, EHR, and sociodemographic data are organized in the PCORnet common data model, which includes age, gender, race/ethnicity, diagnoses, BMI, blood pressure, laboratory, and other variables. Patient zip code is linked to census tract information including percent living in poverty, racial segregation scores, area income inequality, neighborhood built environment, and access to healthy foods. OneFlorida collaborators use these data for cohort discovery, randomized trials, and observational studies in conjunction with the OneFlorida Minority Education Program (MEP) for under-represented minority investigators, including those from two Florida HBCUs. OneFlorida is currently the site for four NIH funded trials, a PCORI CDRN, and Florida Department of Health Tobacco funds which includes MEP mentees using the Data Trust.

**Implications for D&I research:** Using linked health and social determinants data combined with the MEP for implementation science research is a critical component for addressing health disparities.

**Primary funding source:** Patient-Centered Outcomes Research Institute.

### S84 Disseminating and sustaining medical-legal partnerships: Shared value and social return on investment

#### James Teufel

##### Public Health, Mercyhurst University, Erie, PA, 16504, USA

**Background:** The availability of civil legal aid predicts health outcomes, and point estimates support that the majority of low income households have at least one unmet civil legal need. These legal needs typically align with social determinants of health such as income, insurance, education, housing, immigration, disability, and food security. Unmet legal needs have a negative impact on individuals, communities, and populations. However, most civil legal needs of low income households go unaddressed. For example, half of low income people who seek legal aid services are turned away due to the low supply of civil legal aid attorney time. To improve health systems and access to justice, the medical-legal partnership (MLP) model was developed. There are approximately 100 MLPs across 38 U.S. states and territories. More than 500 legal and healthcare organizations have partnered to form MLPs. MLPs serve more than 55,000 low-income people each year. The National Center for MLP aims to reach one million participants by 2023, which will require sustainable dissemination of the MLP model.

**Methods:** The 40-item Sustain Tool v2.0, developed and validated by Washington University in Saint Louis, was tailored to nationally evaluate MLP sustainability across eight dimensions: environmental support, funding stability, partnerships, organizational capacity, program evaluation, program adaptation, communications, and strategic planning. Additionally, a case study approach was used to exemplify the social return on investment of MLP, and findings were linked to Schedule H, which is relevant to non-profit hospital community benefit requirements.

**Findings:** Evaluation results support a social return on investment to communities of greater than 3000 %, including a direct financial return on investment of greater than 200 % to healthcare providers. Findings also identified key areas of strength and weakness with regard to sustainability of the national MLP network across eight dimensions.

**Implications for D&I research:** Unmet legal needs adversely influence health. However, MLP improves access to civil legal aid, which in turn remediates health-harming legal issues of patients and improves the effectiveness of health systems. Business case methods can be used to support program dissemination and sustainability, and existing sustainability tools can be adapted for process improvement of innovative interdisciplinary approaches to address social determinants of health.

**Primary funding source:** Internal university funding.

